# Understanding the immunosuppressive microenvironment of glioma: mechanistic insights and clinical perspectives

**DOI:** 10.1186/s13045-024-01544-7

**Published:** 2024-05-08

**Authors:** Hao Lin, Chaxian Liu, Ankang Hu, Duanwu Zhang, Hui Yang, Ying Mao

**Affiliations:** 1 Department of Neurosurgery, Huashan Hospital, Fudan University, Shanghai, People’s Republic of China; 2 Institute for Translational Brain Research, Shanghai Medical College, Fudan University, Shanghai, People’s Republic of China; 3 National Center for Neurological Disorders, Huashan Hospital, Shanghai Medical College, Fudan University, Shanghai, People’s Republic of China; 4 Shanghai Key Laboratory of Brain Function Restoration and Neural Regeneration, Shanghai Clinical Medical Center of Neurosurgery, Neurosurgical Institute of Fudan University, Huashan Hospital, Shanghai Medical College, Fudan University, Shanghai, People’s Republic of China; 5 Children’s Hospital of Fudan University, and Shanghai Key Laboratory of Medical Epigenetics, International Co-Laboratory of Medical Epigenetics and Metabolism, Ministry of Science and Technology, Institutes of Biomedical Sciences, Fudan University, Shanghai, 200032 People’s Republic of China; 6 State Key Laboratory of Medical Neurobiology and MOE Frontiers Center for Brain Science, Shanghai Medical College, Fudan University, Shanghai, People’s Republic of China

## Abstract

Glioblastoma (GBM), the predominant and primary malignant intracranial tumor, poses a formidable challenge due to its immunosuppressive microenvironment, thereby confounding conventional therapeutic interventions. Despite the established treatment regimen comprising surgical intervention, radiotherapy, temozolomide administration, and the exploration of emerging modalities such as immunotherapy and integration of medicine and engineering technology therapy, the efficacy of these approaches remains constrained, resulting in suboptimal prognostic outcomes. In recent years, intensive scrutiny of the inhibitory and immunosuppressive milieu within GBM has underscored the significance of cellular constituents of the GBM microenvironment and their interactions with malignant cells and neurons. Novel immune and targeted therapy strategies have emerged, offering promising avenues for advancing GBM treatment. One pivotal mechanism orchestrating immunosuppression in GBM involves the aggregation of myeloid-derived suppressor cells (MDSCs), glioma-associated macrophage/microglia (GAM), and regulatory T cells (Tregs). Among these, MDSCs, though constituting a minority (4–8%) of CD45^+^ cells in GBM, play a central component in fostering immune evasion and propelling tumor progression, angiogenesis, invasion, and metastasis. MDSCs deploy intricate immunosuppressive mechanisms that adapt to the dynamic tumor microenvironment (TME). Understanding the interplay between GBM and MDSCs provides a compelling basis for therapeutic interventions. This review seeks to elucidate the immune regulatory mechanisms inherent in the GBM microenvironment, explore existing therapeutic targets, and consolidate recent insights into MDSC induction and their contribution to GBM immunosuppression. Additionally, the review comprehensively surveys ongoing clinical trials and potential treatment strategies, envisioning a future where targeting MDSCs could reshape the immune landscape of GBM. Through the synergistic integration of immunotherapy with other therapeutic modalities, this approach can establish a multidisciplinary, multi-target paradigm, ultimately improving the prognosis and quality of life in patients with GBM.

## Introduction

Glioblastoma (GBM) is categorized as a WHO grade IV glioma [1], representing the most prevalent, primary, and malignant tumor in the brain, and is recognized for its crazy invasiveness. The median survival time of GBM cases is roughly 12.5–15 months, with 2-year and 5-year survival rates of merely 25% and 10%, respectively [2]. The standard therapeutic approach for GBM typically involves surgical intervention complemented by chemotherapy, radiotherapy (RT), or targeted therapy [3]. Nevertheless, the treatment efficacy for GBM remains suboptimal due to the considerable genetic variability and intratumoral heterogeneity inherent to GBM [4]. Recently, the impact of the tumor microenvironment (TME), particularly the immunosuppressive milieu, on the heterogeneity of GBM and its immune "cold" environment has been increasingly recognized [5, 6].

The onset of GBM can be conceptualized through the 'Swiss cheese model', which represents a culmination of successive failures in various host defense mechanisms [7]. Notably, the immune system serves as the ultimate bulwark against GBM initiation and progression. Vigilantly surveilling within the body, the immune system engages with cancer throughout its developmental stages. An imbalance in this intricate interaction underscores that cancer, beyond uncontrolled cellular proliferation, also represents a manifestation of immune dysfunction. From this vantage point forward, immunotherapy has become an inherent approach to cancer treatment [8]. Although immunotherapies targeting programmed cell death protein 1 (PD-1) or cytotoxic T-lymphocyte-associated protein 4 (CTLA-4) have shown efficiency in certain tumors [9], their consistent failures in the case of GBM are attributed to its classification as an “immunologically cold” tumor. GBM typically manifests minimal expression of neoantigens, exacerbating the immunosuppressive milieu through numerous immune checkpoints and immune-inhibitory cytokines [10]. Moreover, owing to its significant intratumoral heterogeneity, the positive responses observed in a small cluster of patients to immunotherapies or other treatment modalities cannot be extrapolated to represent the overall treatment sensitivity of GBM. Consequently, patients’ responses to GBM treatments are frequently transient, and tumor recurrence is nearly universal. These challenges underscore the imperative necessity of enhancing existing GBM treatment strategies.

**Fig. 1:**
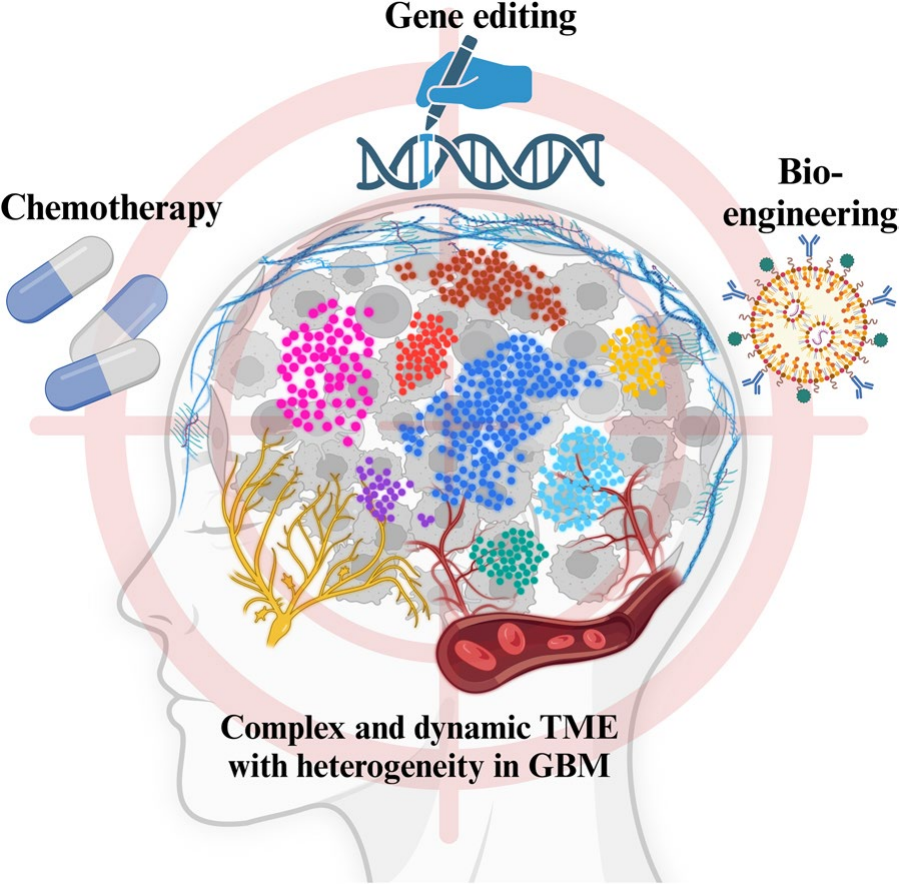
The current challenges of treatment in GBM. Due to its highly dynamic and complex microenvironment components and unique intratumoral heterogeneity, GBM is in urgent need of one or more combination therapies for precise target attacks. These therapies can be drugs, exogenous editing methods, new bioengineering, and so on

Hence, investigating the interplay between the TME, with particular emphasis on some specific components, and tumors and intervening in this interaction holds significant therapeutic promise for regulating tumor immunosuppression [11]. This review encapsulates the immunomodulatory processes and associated molecular characteristics within the immunosuppressive milieu of GBM. The latest research concentrates on delineating the component of TME within these processes, intending to selectively modulate the immunosuppressive microenvironment of GBM, thereby offering potential therapeutic avenues. Figure 1 shows the current challenges of treatment in GBM.

## The immune regulation in glioblastoma

Two cell types can be simply described the central nervous system (CNS), which are glia and neurons, and glioma originate from glia, which include ependymal cells, microglia, astrocytes, and oligodendrocytes [12]. The heterogeneity of TME in GBM shows considerable variability, and the crosstalk between malignant cells and microenvironment is critical for tumor cell proliferation and migration, contributing to the suppression of infiltration and activation of T cells. The major infiltrating cells in the glioma TME are immune cell populations like tumor-associated myeloid cells (TAMCs), which include tumor-associated macrophages (TAMs) and microglias, myeloid-derived suppressor cells (MDSCs), dendritic cells (DCs), and neutrophils [13]. Microglias are distributed throughout the CNS and play a crucial component in regulating immunity homeostasis in the brain. It is the resident TAMs of the CNS and secretes immunosuppressive factors like interleukin-10 (IL-10) and transforming growth factor-β (TGF-β) or other anti-tumor stimulating factors like IL-12 and tumor necrosis factor-α (TNF-α) based on the “heat” or “cold” status of the TME [14]. It has been shown that in GBM, TAMs lack the costimulatory molecules that are essential for the activation of lymphocytes, like CD40, CD86, and CD80, and secreting IL-6, IL-1β, and TNF-α, which are important for the response of innate immune [15]. At the same time, their ability to make the leukocyte antigen (HLA) class II molecules upregulation is impaired but showed increased expression in immunosuppressive ligands like B7-H1 and Fas ligand [16, 17]. MDSCs are heterogeneous and come from immature bone marrow cells that are recruited during tumorigenesis and then infiltrated into tumors, promoting vascularization and becoming major mechanisms of immune surveillance, including polarization of M1 macrophages, antigen presentation of DC, cytotoxicity of natural killer cells (NK cells), and activation of T cells [18]. They have substantial overlap with TAM in the GBM mouse model: They have the phenotypic characteristics of M1 and M2 macrophages and exhibit important functional and phenotypic plasticity based on their local TME [19]. Moreover, CD33^+^ MDSC have been discovered at higher levels in the peripheral blood (PB) of GBM patients than in healthy persons, and healthy persons-derived CD14^+^ monocytes (MONs) exposed to GBM cells may gain MDSC-like features, like upregulating the production of immunosuppressive factors like B7-H1, IL-10, and TGF-β, and inducing apoptosis in activated lymphocytes [20].

The blood–brain barrier (BBB) is one of the key components of the adaptive changes in TME. The BBB, which, like a semipermeable membrane, consists of endothelial cells (ECs), foot processes from astrocytes, and pericytes, separates the CNS from the peripheral immune system so that naive T cells cannot cross the BBB, but activated T cells can [21]. Thus, it rigidly regulates the lymphocytes infiltrating the CNS, and therefore, there is an overall decrease in immune surveillance in GBM compared to other tumors. As the GBM progresses, it can disrupt the BBB and induce inflammation, which leads to leakage and damage of peripheral blood vessels, resulting in inadequate oxygen delivery, and insufficient blood flow creates hypoxic regions within the tumor, which subsequently attract macrophages and further enhance tumor tumorigenicity [22].

**Fig. 2:**
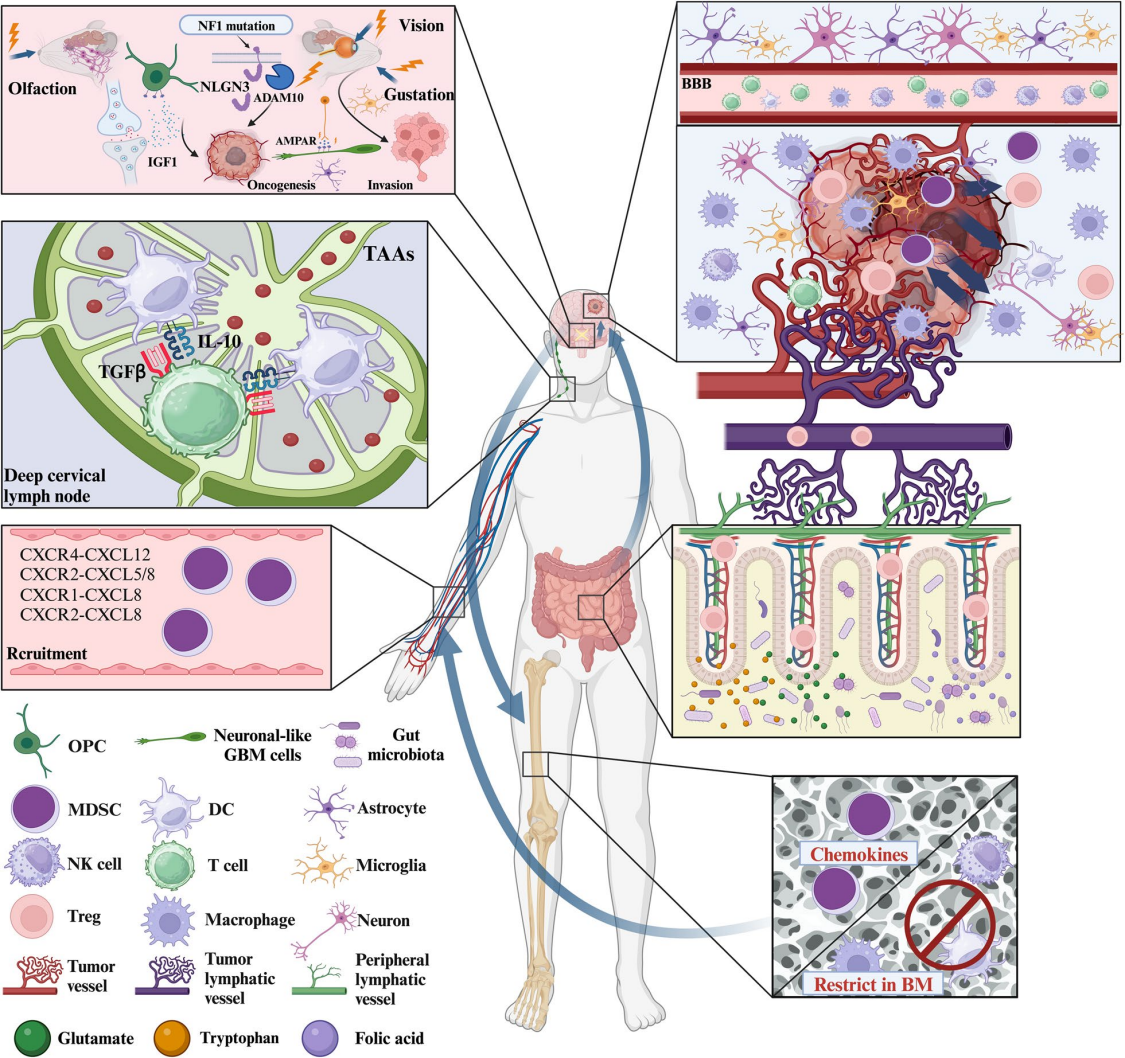
Molecular mechanism of crosstalk between GBM and systemic immunity. GBM is the most common and lethal brain malignancy in adults. It not only leads to the reprogramming of local immunity in the brain but also affects peripheral immunity to some extent. The microenvironment of GBM is complex, and immune cells are heterogeneous and are mainly composed of MDSCs, microglia, astrocytes, Tregs, blood vessels, and the ECM. The secretion of numerous cytokines, chemokines, and metabolites by GBM can affect the systemic immune system through the blood, lymphatic vessels, and paracrine pathways. Similarly, these channels can also affect the occurrence and development of GBM. *OPCs* oligodendrocyte progenitor cells; *AMPAR* α-amino-3-hydroxy-5-methyl-4-isoxazole-propionica

Based on the molecular characteristics encompassing gene expression profiles, DNA methylation profiles, and transcription profiles in GBM, GBM can be classified into three distinct subtypes: mesenchymal, proneural, and classical, each marked by specific molecular features. The gene expression of the proneural subtype, including the receptor tyrosine kinase (RTK) I/LGm6 DNA methylation group, exhibiting molecular alterations such as cell cycle-dependent kinase 4 (CDK4) and platelet-derived growth factor receptor alpha (PDGFRA) amplification, predominates among younger adults. The gene expression of the classical subtype, including the RTK II DNA methylation group, is distinguished by frequent epidermal growth factor receptor (EGFR) amplification and deficiency of cyclin-dependent kinase inhibitor 2A/B (CDKN2A/B). The gene expression of the mesenchymal subtype is defined by the deficiency of neurofibromin 1 (NF1) and heightened infiltration of TAMs. While most GBM manifests these three molecular subtypes, the coexistence of multiple molecular phenotypes is commonplace, all of which are intricately linked to telomerase reverse transcriptase (TERT) promoter mutations [1, 23, 24]. Another classification method, leveraging single-cell sequencing technology, focuses on the sub-cellular subtyping of GBM. This approach categorizes internal tumor cells into distinct subclones, revealing the internal heterogeneity of GBM. The identified tumor cell subtypes include mesenchymal-like (MES-like), neural progenitor-like (NPC-like), oligodendrocyte progenitor-like (OPC-like), and astrocyte-like (AC-like) subclones. This refined classification offers a comprehensive insight into the diverse cellular composition within GBM [25]. Each subtype corresponds to a unique immunosuppressive microenvironment, with inherent heterogeneity within each subtype. The immunosuppressive processes in GBM primarily involve intricate crosstalk among genetic alterations, epigenetic changes, metabolite regulation, and various microenvironmental components. These influencing factors encompass glioma-associated macrophages/microglias (GAMs), MDSCs, and T cells. Signaling factors such as TNF-α [26], NF1 [24], and IL-33 [2] are employed, impacting pathways such as TGF-β/Smad and nuclear factor kappa-B (NF-κB) pathways [27, 28]. This intricate interplay with immune cells further fortifies the immunosuppressive microenvironment through CTLA-4, PD-1, and T-cell immunoglobulin and mucin-domain containing-3 (TIM-3) among other targets [29–33]. Moreover, individuals with GBM frequently manifest systemic immunosuppression, characterized by the inhibition of activation of T cells through the IL-10-TGF-β pathway following DCs activation at the deep cervical lymph nodes [5]. This activation is triggered by tumor-associated antigens (TAAs) drained from the GBM. Additionally, peripheral components such as gut microbiota can undergo metabolic changes influenced by GBM, leading to the activation of more regulatory T cells (Tregs). These Tregs are then recruited to the GBM microenvironment, where they exert immunosuppressive effects [34]. Sometimes, the older age of onset [35] and glucocorticoids [36, 37] can also lead to systemic immunosuppression. In both the blood pool and bone marrow pool, chemokines secreted by GBM play a pivotal role in activating and recruiting MDSCs to enter the GBM microenvironment. Simultaneously, they can prohibit the activation of normal immune cells in the bone marrow pool, mediating immunosuppression [38, 39]. This process can be elucidated in more detail in subsequent sections. Notably, within the local microenvironment of GBM, the BBB undergoes modifications induced by GBM, rendering it selectively permeable for immune cells [40, 41]. This selective permeability allows TME to reject normal immune cells while facilitating the entry of immunosuppressive cells. The intricacies of immunosuppression within the GBM microenvironment will be expounded upon in the following sections. Figure 2 illustrates the systemic immune response in the presence of GBM.

### The status of epigenetic mechanisms in glioblastoma regarding immune regulation

In GBM, immune attacks can instigate epigenetic changes in tumor cells, subsequently influencing their immune responsiveness. However, the impact of immune attacks varies among different tumor subtypes. These epigenetic alterations encompass not only histone modifications [42], chromatin remodeling [43], and DNA methylation [44], but also specific non-coding RNA molecules (such as miRNAs and lncRNAs) [45] and metabolites that exert post-transcriptional modifying effects. Current research suggests that in GBM, epigenetics pertains to the regulation of various pathways, including the Notch [46], Hedgehog [47], and WNT pathways [48].

In spontaneous GBM mouse models, activating colony-stimulating factor 1 receptor (CSF-1R) signaling can induce increased methylation in the interferon regulatory factor 8 (IRF8) promoter region. This methylation reduces GBM sensitivity to interferon-gamma (IFN-γ) and responsiveness to TAMs, ultimately promoting immune evasion [49]. The core area of GBM is characterized by extreme hypoxia, which induces the m6A demethylase alkB homolog 5 (ALKBH5). Inactivation of ALKBH5 significantly inhibits the recruitment of hypoxia-induced TAMs and immunosuppression. However, hypoxia-induced ALKBH5 also reduces m6A deposition in the lncRNA nuclear enriched abundant transcript 1 (NEAT1), promoting the repositioning of splicing factor proline and glutamine-rich (SFPQ) near the promoter of C-X-C motif chemokine ligand 8 (CXCL8). This leads to the re-expression of CXCL8/IL-8, partially restoring TAM recruitment and tumor progression [50]. Hence, this process is bidirectional, underscoring the complexity of epigenetic regulation in developing GBM and its role in intratumoral heterogeneity. In another context involving m6A-related epigenetic regulation, the YY1-CDK9 transcription complex increases the programmatic expression of m6A, subsequently downregulating MHC-related genes and interferon-related genes. Notably, the dataset in Cancer Genome Atlas (TCGA) about GBM reveals a correlation between the transcription complex and low CD8^+^ T cell infiltration. Targeting the YY1-CDK9 transcription complex can enhance GBM's responsiveness to PD-1 therapy [51].

Furthermore, lysine demethylase 6B (KDM6B) exhibits high expression in MDSCs within the GBM microenvironment. Specific knockdown of KDM6B in MDSCs enhances proinflammatory pathway activity and improves the prognosis of mice with GBM. KDM6B deficiency inhibits secretion of immunosuppressive mediators such as MAF BZIP transcription factor B (MAFB), suppressor of cytokine signaling 3 (SOCS3), and signal regulatory protein alpha (SIRPA), thereby enhancing the efficacy of anti-PD-1/programmed cell death 1 ligand 1 (PD-L1) therapy [52]. In humans, presence of X chromosome inactivation escape gene KDM6A [53] results in lower CD8^+^ T cell levels in male GBM microenvironments than in female GBM microenvironments [54]. Moreover, T cells in the male GBM microenvironment are more prone to exhaustion. Another transcription factor (TF), zinc finger protein 148 (ZNF148), promptly binds to pentraxin 3 (PTX3) promoter region and upregulates PTX3 expression. In GBM, downregulating the expression of ZNF148 could diminish PTX3 expression, consequently reducing the proliferation and migration of transformed DCs (t-DCs) and restraining the expression of costimulatory, thereby diminishing the tumor-promoting ability of t-DCs in vivo [55].

Regarding metabolic regulation, acetylation has emerged as a prevalent epigenetic modification in GBM. Fatty acids and acetate act as regulators of acetylation. Fatty acids undergo oxidation to generate acetyl-CoA, inducing the acetylation of NF-κB/RelA, which upregulates CD47 transcription, thereby enhancing the phagocytic resistance of GBM cells [56]. Acetate indirectly activates pyruvate dehydrogenase (PDH) by facilitating the conversion of pyruvate to acetyl-CoA, resulting in increased histone acetylation and modulating the stemness of glioblastoma stem cells (GSCs) [57]. Acetate salts inhibit the expression of histone deacetylase (HDAC), promote multiple miRNA expression, and hinder GBM cell proliferation, invasion, migration, and angiogenesis. Additionally, these acetate salt molecules regulate genes associated with mammalian targets of rapamycin complex 2 (mTORC2), thereby impeding GBM development [58]. At the same time, lactate is traditionally viewed as a metabolic byproduct of tumor metabolism. Recent research [59] highlights its role in enhancing chromatin accessibility and histone acetylation through aerobic metabolism and ATP-citrate lyase (ACLY) dependency. This protective mechanism shields malignant cells from death caused by nutrient deprivation [60]. Moreover, lactate accumulation induces the lactylation of histone lysine [59]. In GSCs with enhanced glycolysis, lactate induces the lactylation of H3K18, promoting the expression of the lncRNA LINC01127. This, in turn, activates the MAP4K4/JNK pathway, enabling GSCs to sustain self-renewal [61]. Palmitoylation, a post-translational modification (PTM) crucial for regulating protein transport, stability, and cellular localization, is catalyzed by palmitoyl transferases, such as Asp-His-His-Cys 9 (DHHC9). In GBM cells, DHHC9 palmitoylates glucose transporters 1 (GLUT1), enhancing its membrane localization and promoting glycolysis and tumor progression. Knocking out DHHC9 inhibits this process, offering potential improvements in patient outcomes [62].

In some specific cases, EGFR-chimeric antigen receptor T cell (CAR-T) therapy (EGFR-CAR-T) effectively prohibits the progress of GBM cells in vitro and of those derived from malignant cells and patient-derived xenografts in mice [63, 64]. However, mice quickly resist EGFR-CAR-T therapy, limiting its potential clinical application. Genomic and transcriptomic analyses of GBM cells co-cultured with EGFR-CAR-T reveal increased immunosuppressive gene activity and enhancer activity. Bromodomain-containing protein 4 (BRD4), another epigenetic factor acting on promoter and enhancer regions, is important for the activation of these immunosuppressive genes [65–67]. Inhibiting BRD4 with the inhibitor JQ1 disrupts the activation of these immunosuppressive genes. The treatment combining JQ1 and EGFR-CAR-T suppresses the metastasis and development of GBM cells, extending the survival time of mice [63]. The mutation of H3.3-G34R/V is common in diffuse midline gliomas (DMG) [1], whereas the mutation in G34R of pediatric high-grade gliomas (pHGGs) can lead to functional loss of DNA repair, resulting in genomic instability and the accumulation of extrachromosomal DNA. Leaked DNA can activate the cGAS/STING (cyclic GMP-AMP synthase/stimulator of interferon genes) pathway, inducing the release of immunostimulatory cytokines. Combination therapy involving DNA damage response inhibitors (DDRi) and RT in H3.3-G34R pHGG mice can significantly increase median survival [68]. Table 1 shows the epigenetic alterations associated with immune regulation in GBM [49–52, 54, 63, 66, 68–84].

**Table 1:** Epigenetic alterations in glioblastoma associted with immunity

Target	Modifying in epigenetic	Impact on the immune microenvironment of GBM	Reference
IRF8	DNA Methylation	Promote immune evasion and transformation of GBM into mesenchymal types	[49]
OLFML3	CLOCK mediated transcriptional upregulation	Promote self-renewal of GSC and recruit TMAs	[50]
YY1	m6A modifying	Promote Treg infiltration	[51]
KDM4A	Demethylation of H3K9me3	Inhibit cell autophage	[52]
KDM6A	Demethylation of H3K27me3	Promote CD8^+^ T cell exhaustion	[54]
KDM6B	Demethylation of H3K27me3	Promote the immunosuppressive function of myeloid cells	[63]
IDH	DNA Methylation	Suppress CD3^+^ & CD8^+^ T cell infiltration	[66]
IFN-α	BET & HDAC modifying	Regulate the expression of ISG and PD-L1	[68]
BRD4	Promote H3K27ac modifying	Maintain immunosuppressive microenvironment	[69]
ALKBH5	m6A demethylation	Recruite TAM	[70]
Integrin β1	Increased chromatin accessibility	Recruite MDSC	[71]
EZH2	Promote H3K27me3 in the promoter of iNOS and TNFα	Promote the formation of M2 type macrophage	[72]
IGFBP1	m6A modifying	Sustain immunosuppressive microenvironment	[73]
IL-7	Methylation	Promote immune evasion	[74]
CXCL9/10	H3K27me3	Suppress T cell recruitment	[75]
GPX7	DNA Methylation	Inhibit innate immunity and adaptive immunity	[76]
MTAP	DNA Methylation	Suppress macrophage recruitment	[77]
MIR155HG	Reduce methylation levels in promoter	Suppress immunocell infiltration	[78]
FOXP3	Demethylation	Affect the generation of Treg and CD4^+^ T cell	[79, 80]
LSD1	Histone demethylase	Inhibit p53 pathway	[81]
JMJD3	Histone demethylase	Inhibit p53 pathway	[82]
KAT8	H4K16ac	Promote the production of tumor-associated microglia	[83]
H3.3	G34R/V	Regulate the cGAS/STING pathway	[84]

*GBM* Glioblastoma; *GSC* Glioma stem cell; *TAM* Tumor associated macrophage; *Treg* regulatory T cells; *BET* Bromodomain and extraterminal domain; *HDAC* Histone deacetylase; *ISG* Interferon-stimulated genes; *MDSC* Myeloid-derived suppressor cells

### Role of the transcriptome in the TME of glioblastoma

The transcriptome generally refers to the collection of all transcription products within cells under physiological conditions [85]. GBM is defined as a kind of tumor with great changes in the transcriptome that are dysregulated transcriptome. Current findings from multitranscriptomic analyses indicate that, in comparison to those in other tumors, infiltrating lymphocytes in GBM TME express various co-inhibitory immune checkpoints and demonstrate significant functional impairments, resembling a phenotype consistent with T cell exhaustion [86]. This exhaustion phenotype is characterized by the expression of HLA-DR^+^, TIM-3^+^, PD-1^+^, CD39^+^, and CD45RO^+^[87]. Through techniques such as spatial transcriptomics (ST) and single-cell RNA sequencing (scRNA-seq), it becomes evident that GBM cells could induce local environmental changes through signaling and structural alterations. These changes contribute to chemotherapy resistance and immune escape. Notably, the subtypes of GBM cells present in different microenvironment locations vary, and this situation may evolve due to species changes and tumor recurrence. The ability to observe and verify these changes at the single-cell level [28] explains why certain treatment strategies, effective in cell and animal models, may be less effective in patients. Moreover, the responsiveness of GBM to specific treatments may vary among patients and could be diminished by recurrence.

EZH2-92aa, encoded by the circular form of enhancer of zeste 2 (EZH2), overexpresses within GBM as well as contributing to the immune evasion of GSCs against NK cells [88]. Moreover, fibroleukin 2 (FGL2) exhibits heightened expression in GSCs and GBM cells. FGL2 suppresses CD103^+^ DC polarization induced by granulocyte–macrophage colony-stimulating factor (GM-CSF) by inhibiting NF-κB, p38, and signal transduction and transcription factor 1/5 (STAT1/5) activation. Low FGL2 and high GM-CSF expression correlate with CD8^+^ T cell infiltration and improve prognosis [89]. 67% of GBM samples highly expresse chondroitin sulfate proteoglycan 4 (CSPG4), and targeting CSPG4 by CAR-T effectively controls GBM growth in a mouse model [90]. Under normoxic conditions, GBM cells inhibit T cell proliferation by expressing indoleamine 2,3-dioxygenase-2 (IDO2). However, IDO2 is downregulated in GBM cells under hypoxic conditions, restoring T cell proliferation possibly through the reduction of kynurenine, a metabolite produced by GBM cells [91]. Moreover, GBM cells, especially those in the GBM mesenchymal subtype, highly express guanylate-binding protein 5 (GBP5). Increased GBP5 expression is positively related to poor outcomes in patients with GBM. High expression of GBP5 promotes the proliferation, migration, and invasion of GBM both in vitro and in vivo, while RNA interference-mediated silencing of GBP5 yields adverse consequences. Targeting GBP5 in GBM impedes the development of GBM and extends the mice's survival, and the Src/ERK1/2/MMP3 axis is crucial for GBP5-mediated malignant cell invasiveness [92].

STAT3 plays a crucial role in GBM development, contributing to early GSC formation and the mesenchymal transformation (MET) of GBM upon activation. As a key driver of stem cell transcription factors, STAT3 has become a significant target for GBM treatment. The STAT3 inhibitor BZA reduces the self-renewal capacity and expression of stemness markers in GSCs [93]. In the mesenchymal subtype or isocitrate dehydrogenase 1 (IDH1) wild-type (WT) subtype of GBM, elevated levels of herpes virus entry mediator (HVEM) have been observed using multiple omics technologies [94]. HVEM is implicated in various immune regulatory processes, including promoting Treg differentiation, inhibiting antigen processing, and presenting major histocompatibility complexes I (MHC I) molecules and αβT. Furthermore, the expression of PD-1, CTLA-4, TIM-3, V-domain Ig suppressor of T cell activation (VISTA), and lymphocyte activating 3 (LAG3) positively correlates with HVEM, suggesting its potential role in immune suppression within the GBM microenvironment [94, 95]. High levels of lysosomal-associated membrane protein 2A (LAMP2A) in GBM and the TME are associated with temozolomide (TMZ) resistance and tumor progression. Its elevated expression is associated with poor overall survival (OS) in patients with GBM. Highly expressed LAMP2A in GSCs facilitates their acquisition of stemness while decreasing the release of IFN-γ in the TME. Loss of LAMP2A weakens GSC-mediated tumorigenic activity [96].

Identifying various distributed genes in GBM establishes a valuable reference database for researchers, offering insights into potential therapeutic targets. Table 2 presents the current GBM genes, biological targets, and immune-related targets [17, 47, 56, 60, 69, 97–240]. So, characterizing the transcriptome of GBM has yielded profound insights into the highly variable transcriptomic features of GBM and its microenvironmental cell components. This has transformed our comprehension of GBM, enabling the prediction and customization of treatment strategies. Nevertheless, the functional roles of many gene changes in the GBM transcriptome remain enigmatic [241]. Therefore, the development of methods to predict GBM gene functions using multi-omics techniques and leveraging these predictions for potential targeted therapies represents an innovative predictive framework. This approach holds promise for expanding the repertoire of GBM targets and creating new opportunities for clinical translation.

**Table 2:** Relevant targets in GBM

Target	Relevant mechanism of function	Cell interactions	Impact on GBM and its microenvironment	References
CPT1A/CPT2/ACAD9*	Promote the expression of CD47	Macrophage	Promote anti-phagocytic function of GBM cells and tumor recurrence	[17]
FASN	Increase FA synthesis and prevent ERS	None	Promote GBM progression and inhibit apoptosis	[47, 56]
CDKN2A	Involved in the lipid peroxidation process	None	Induce GBM cell ferroptosis	[60]
SLIT2*	Promote cell migration	Macrophage and microglia	Promote TAM and tumor angiogenesis	[69]
CCL2*	Involved in the TP53 mutation activating pathway	Macrophage and microglia	Promote macrophage and microglia migration	[97]
CSF2*	Regulate bone marrow cell recruitment	Macrophage	Promote microglia migration	[98]
COX2	Stimulate the growth of GSC	None	Promote EMT and GSC proliferation	[99]
LOX/SPP1*	Regulate macrophage recruitment	Macrophage	Promote TAM and tumor angiogenesis	[100]
GPX4	Inhibit lipid accumulation	Neutrophil	Inhibit ferroptosis in GBM	[101]
ACC1/2	Biotinize the protein	None	Maintain the stemness of GSC	[102]
TNFα*	Involved in the NF-κB activating pathway	Macrophage and microglia	Recruite macrophage and microglia migration	[103]
DGAT1	Maintain lipid homeostasis	None	Inhibit the lipid peroxidation process	[104]
CD36	Regulate the expression of apoptosis-related receptors	MSC	Inhibit GBM apoptosis	[105]
CCL5/CX3CL1*	Involved in the NF1 deficiency process	Macrophage and microglia	Promote macrophage and microglia migration	[106]
CX3CR1*	Regulate microglia recruitment	Microglia	Promote microglia migration	[107]
MIF/CD74/CXCR2*	Regulate bone marrow cell recruitment	MDSC and microglia	Sustain immune suppression of TME	[108]
SCD	Maintain lipid homeostasis	None	Inhibit the lipid peroxidation process	[109]
Lactate*	Participate in TCA cycle regulation	Macrophage and microglia	Promote the chromatin accessibility of ARG1	[110]
LXR	Regulate the cellular response to cholesterol	None	Promote GBM cell death	[111]
Cholesterol	Regulate cellular lipid metabolism homeostasis	None	Inhibit GBM cell death	[112]
CHI3L1*	Involved in the PI3K/AKT/mTOR pathway	Macrophage	Promote macrophage and microglia migration	[113]
CSF1/CSF-1R/IFN-γ*	Regulate bone marrow cell recruitment	Macrophage	Promote the recruitment and activation of TAM	[114]
P-selection*	Mediated leukocyte adhesion	Microglia	Promote the polarization of microglia to immunosuppressive phenotype	[115]
ICOSLG*	Involved in the TNFα/NF-κB pathway	Treg	Increase Treg and GSC generation and IL-10 production	[116]
PGE2*	Involved in the ARS2/MAGL pathway	Macrophage	Stimulate β-catenin activation of GSC and TAM polarization	[117]
Kyn*	Activate the AHR and CD39	Macrophage	Promote TAM migration	[118]
CXCL1/2*	Regulate bone marrow cell recruitment	MDSC and microglia	Disrupt CD8 + T cell accumulation in GBM	[119]
OLFML3/CLOCK*	Regulate gene transcription	Macrophage	Recruite TAM	[120]
LGMN*	Interact with HIF-1α	Microglia	Promote TAM polarization	[121]
CXCL8*	Activate PI3K/AKT and NF-κB signaling	Neutrophil	Sustain an M2-like TAM phenotype	[122]
PD-L1*	Activated by Wnt ligand and EGFR	T cell	Inhibit T-cell function	[123]
APOBEC	Catalyze mRNA cytosine to uracil (C-to-U) base modification	None	Predict the prognosis of GBM patients	[124]
POLE/POLD1	Unknown	None	Predict the prognosis of GBM in Children	[125]
PTEN*	Increase the expression of immunosuppressive cytokines	T cell	Inhibit anti-PD-L1 treatment response	[126]
p-ERK*	Involved in the ERK pathway	Microglia	Induce microglial M2 polarization	[127]
MHC II	Down-regulated expression in GBM	DC	Suppressive immunoreaction	[128]
CTLA-4*	Tregs mediated by anti–CTLA-4 coengaging activating Fc-γ receptors	T cell	Inhibit T-cell function	[129]
TIM-3*	Involved in the AKT-GSK3β-IRF1 pathway	T cell	Induce microglial M2 polarization	[130]
IL-13Rα2	Bind to EGFRvIII and activite the RAS/RAF/MEK/ERK and STAT3 pathways	None	Promote GBM cell proliferation	[131]
HER2/ErbB2	Activate multiple kinases	T cell	Promote GBM cell proliferation	[132]
EGFRvIII	Constitutively active RTK	None	Resistance of GBM to radiotherapy and chemotherapy	[133]
IDO*	Convert tryptophan into Kyn	Microglia	Promote immunosuppressive microenvironment	[134]
ARG1*	Catalyze the hydrolysis of L-arginine to urea and L-ornithine	Microglia	Block T cell proliferation	[135]
LAG-3*	Immune checkpoint regulator	T cell	Inhibit T cell function	[136]
CD47*	Immune checkpoint regulator	MDSC and microglia	Promote the anti-phagocytosis function of GBM	[137]
CD73*	Catalyze the synthesis of adenosine	T cell	Inhibit T cell proliferation	[138]
MAGE1*	Tumor-associated antigens	T cell	Tumor-associated antigens	[139]
AIM-2	Tumor-associated antigens	DC	Tumor-associated antigens	[140]
CD133	Neural stem cell and GSC marker	None	Facilitate the formation of tumor sphere	[141]
MGMT	Repair DNA damage	None	Assist GBM cells in defending against radiation therapy	[142]
IDH	Participate in TCA cycle regulation associated with NADPH	None	Assist GBM cells in defending against radio- and chemotherapy	[143]
dCK	Phosphorylation of chemotherapeutics	None	Modulate GBM cell chemotherapy resistance	[144]
OPN*	Potent chemokine for macrophages	Macrophage and microglia	Maintain the M2 macrophage gene signature and phenotype	[145]
MARCO*	Transcriptional regulatory networks component	Macrophage	Induce a phenotypic shift towards mesenchymal cellular	[146]
TERT	Component of telomerase	None	Affect GBM cell recurrence and chemotherapy resistance	[147]
CXCL12	Key mediator of GBM mesenchymal activation	None	Mediate GBM resistance to radiotherapy in the SVZ	[148]
PDGFRA/EPHA2	RTK family	None	Promote proliferation, survival, and invasion of GBM	[149]
PDGFRB	RTK family	None	Promote proliferation, survival, and invasion of GBM	[150]
OLIG2/SOX2/SALL2/POU3F2/NES	Transcription factors associated with the maintenance of GSC stemness	None	GSC-related markers	[151]
ATRX	Histone chaperone protein	None	Modulate GBM cell response to DNA damage	[152]
TP53	Cancer suppressor gene	None	Regulation of GBM cell proliferation	[153]
NG2	Developmentally important transmembrane proteoglycan	None	Enhence the proliferative ability of GBM cells	[154]
NR4A1/NF1	Pro-apoptotic molecule in cytoplasm	None	Pro-oncogenic molecule in cerebellar GBM	[155]
NOTCH1*	Notch pathway	Macrophage	Regulate the reactivity of TAM	[156]
ASCL1	A proneural transcription factor involved in normal neurogenesis	Neuron	Modulate tumorigenicity of GSC	[157]
AKT	A serine-threonine kinase	None	Promote development of GBM	[158]
MET/HGFR	Receptor tyrosine kinase	None	Promote the mesenchymal transition in GBM cell	[159]
CD99	A transmembrane glycoprotein	None	Regulation of cuproptosis in GBM cell	[160]
HK2	Involved in the glycolysis process	None	Promote GBM growth	[161]
MYC	Regulate the tumorigenic ability of TP53 and PTEN	None	Increase the generation of GSC	[162]
α-KG	An intermediate metabolite in the TAC	None	Promote GBM growth	[163]
Glutamate	Excitatory neurotransmitter in the central nervous system	None	Inhibit GBM cell apoptosis	[164]
Citrate	An intermediate metabolite in the TAC	None	Enhence the effect of glutamate	[165]
SLC7A11	A key ferroptosis marker	None	Modulate the GBM cell ferroptosis	[166]
2-HG	Affect epigenetic regulators	None	Promote GBM progression	[167]
ANXA2	A calcium-dependent phospholipid-binding protein	None	Oncogenic functions in GBM	[168]
PHIP	Involved in GBM motility through focal adhesion	None	Promote migratory potential of GBM cell	[169]
GLUT3	Glucose transporters	None	Promote invasion potential of GBM cell	[170]
IL-10*	Cytokines from monocytes	T cell	Induce T cell apoptosis	[171]
CD27*	Tumor necrosis factor receptor superfamily	T cell	Regulate T cell function	[172]
WT1	Tumor-associated antigens and oncogenous	None	Promote GBM development	[173]
CDK4/6	Regulate G1 to S phase progression	None	Modulate GBM cell proliferation	[174]
MDM2/4	Induce TP53 proteasome-mediated degradation	None	Promote GBM development	[175]
FGFR1/3	Fibroblast growth factor receptor	None	Promote GBM development	[176]
RB1	Cancer suppressor gene	None	Regulation of GBM cell proliferation	[177]
TRADD	Activate NF-κB pathway	None	Endow GBM cells with chemotherapy resistance	[178]
NEFL	Regulate the activation of the mTOR pathway	None	Promote GBM proliferation and invasion	[179]
GABRA1	Gamma-aminobutyric acid receptor	None	Associated with GBM prognosis	[180]
SLC12A5	Involved in ion transport, synapse and neurotransmitter	None	Regulate proliferation of GBM	[181]
SYT1	Calcium-binding protein	None	Promote GBM development	[182]
MKI67	Cellular proliferation regulator	None	Promote GBM development	[183]
HIF	Induce the transcription of numerous downstream target genes	None	Promote GBM or GSC migration and invasion	[184]
B4GALT3	Regulate cell proliferation and invasion via β-catenin and vimentin	None	Promote GBM cell proliferation and invasion	[185]
YKL40	An extracellular matrix glycoprotein	None	As predictor of survival in patients	[186]
GBP2	An interferon-inducible large GTPase	None	Promote GBM cell migration and invasion	[187]
STAT3	Modulate the expression of numerous downstream pathways	None	Promote GBM development and progression	[188]
EGFR	Intracellular tyrosine kinase	None	Promote GBM development and progression	[189]
VIM	The biomarkers of EMT	None	Promote GBM development	[190]
GNB2	Activate the canonical G protein signaling	None	Associated with GBM recurrence	[191]
IGFBP2	Modulator of IGF signaling	None	Promote GBM progression	[192]
PDPN	Type I transmembrane mucin-like glycoprotein	None	Promote the EMT	[193]
DECR1/POLR2F	Unknown	None	As predictor of survival in patients	[194]
NKG2D*	Increased in bone marrow cel mediated by LDH	NK cell	Reduce the NK cell	[195]
HDAC	Mediate histone deacetylation	None	Modulate the cell proliferation, and drug resistance of GBM cell	[196]
PDIA3	Participate in protein folding through its protein disulfide isomerase function	None	As predictor of survival in patients	[197]
H3F3A mutation	Involving H3.3K27M and H3.3G34R/V	None	Promote GBM development in children	[198]
AHNAK2	Unknown	None	Predict the effect of target BRAF V600E therapy	[199]
SOX1	Neural development and neural progenitor pool maintenance	None	Promote self-renewal and proliferation in GSC	[200]
SUSD2	Modulate circRNAs	None	Promote GBM proliferation and aggressiveness	[201]
PIK3CA	Regulate the interaction between GBM cell and neuron	None	Promote GBM development	[202]
CCNB1	Oncogene that regulate the cell cycle	None	Promote GBM development	[203]
CDC6	Regulation of S-phase and M-phase of meiosis	None	Marker of GBM development	[204]
KIF20A/23	Unknown	None	As predictor of survival in patients	[205]
RTK I/II	Receptor tyrosine kinase family	None	As predictor of survival in recurrent patients	[206]
ANKRD10/BMP2/LOXL1/RPL39L/TMEM52/VILL	Unknown	None	As predictor of survival in patients	[207]
ANXA7	Multigene annexin superfamily of Ca2 + regulated and phospholipid-binding protein	None	As predictor of survival in patients	[208]
MARK4	Regulation of microtubule dynamics by phosphorylation of tau protein	None	Promote GBM development	[209]
Delta Max	Enhancer of Myc-dependent transformation	None	Promote GBM cell proliferation	[210]
USP5	Ubiquitin specific protease	None	Promote GBM development and progression	[211, 212]
WWOX	WW domain-containing oxidoreductase	None	Increase proliferation and growth in GBM	[213]
RON	Tyrosine kinase receptor	None	Promote migration and invasion in GBM	[214]
USP10/CCND1	Key factor in cell cycle control	None	Inhibit GBM cell apoptosis	[215]
CELF2	RNA binding protein	None	Maintain the proliferative with clonal and tumorigenic properties	[216]
miR-4763-3p/miR-1915-3p/miR-3679-5p	Unknown	None	As predictor of survival in patients through serum	[217]
SNHG12	Upregulation of MAPK1 and E2F7	None	Promote TMZ resistance in GBM	[218]
NDRG1/GSK-3β	Modulate the cell growth and G0–G1	None	Modulate GBM cell proliferation	[219]
tGLI1/CD44	A tumor-specific transcription factor	None	Promote GBM growth and mesenchymal GSC	[220]
CD41	Blood-borne microvesicle	None	Biomarker for recurrence and survival in GBM patients	[221]
VEGF	A proangiogenic cytokine	None	Promote GBM angiogenesis	[222]
c-Kit	The specific binding of stem cell factors and regulate the activity of RTK	None	Modulate GSC ability in GBM through cell differentiation	[223]
PLK1	Involve polarity regulators and mitotic kinase	None	Endow GBM cells with chemotherapy resistance	[224]
αvβ3*	Promote cell migration and extracellular matrix assembly and remodeling	Macrophage	Recruitment of M2-macrophage	[225]
αvβ5	Promote cell migration and extracellular matrix assembly and remodeling	None	As a functional GSC marker essential for GBM maintenance	[226]
PRMT5	Regulate transcription by targeting histones, nucleosome remodeling and co-repressor complexes, and numerous transcription factors	None	Modulate GBM development	[227]
IGF-1R	Macrophage-derived insulin-like growth factor-1	None	Endow GBM cells with chemotherapy resistance	[228]
mTOR	Atypical serine/threonine protein kinase	None	Modulate GBM development, progression and immunocell infiltration	[229]
Ras	Proto-oncogenes and small GTP-binding proteins	None	Promote GBM development	[230]
PKC	Protein kinase C	None	Promote GBM development	[231]
TGFβ	Initiate an intracellular signaling cascade	None	Promote GBM development	[232]
ROR1/IGFBP5	Facilitates ROR1/HER2 heterodimer formation	None	Promote GSC invasion	[233]
CD155/TIGIT*	Interaction with TIGIT	NK cell	Inhibit the function in NK cell	[234, 235]
ETV2	Activate vascular genes and represses proneural genes to direct endo-transdifferentiation	None	Mediate endothelial transdifferentiation of glioblastoma	[236]
GLUD2	Catalyze glutamate oxidative deamination	None	Modulate GBM cell proliferation	[237]
CD70*	Tumor necrosis factor receptor family	T cell	Selective induction of CD8 + T cell death	[238]
MP31	Compete with LDH to regulate lactic acid metabolism	None	Modulate GBM development	[239]
TMEM131L	Associated with oxidative stress	None	Regulation of GBM cell proliferation	[240]

^*^Indicate this marker is related to immune response in GBM

*GBM* Glioblastoma; *FA* Fatty acid; *ERS* Endoplasmic reticulum stress; *TAM* Tumor associated macrophage; *GSC* Glioma stem cell; *EMT* Epithelial-mesenchymal transition; *MSC* Mesenchymal stem cell; *MDSC* Myeloid-derived suppressor cells; *TME* Tumor microenvironment; *TCA cycle* Tricarboxylic acid cycle; *Treg* T regulatory cells; *Kyn* Kynurenine; *AHR* Aryl hydrocarbon receptor; *DC* Dendritic cell; *RTK* Tyrosine kinase; *dCK* deoxycytidine kinase; *SVZ* Subventricular zone; *EMT* Epithelial-mesenchymal transition; *LDH* Lactate dehydrogenase; *NK* Natural killer; *HDAC* Histone deacetylase; *circRNA* Dysregulated circular RNA; *TMZ* Temozolomide

One of the predominant methods for predicting targets based on the transcriptome involves utilizing databases, patient-derived samples for cell interaction and prognosis analysis, and scRNA-seq. Krishna et al. used scRNA-seq datasets from patient-derived samples [242] and identified that integrin subunit beta 2 (ITGB2) was highly enriched in immune and stromal environments, including T cells, fat cells, microglias, macrophages, and newly formed oligodendrocytes through scRNA-seq datasets from patient-derived samples. Unique genes within these cell populations include collagen type VI alpha 3 chain (COL6A3), TNF superfamily member 9 (TNFSF9), and serpin family E member 1 (SERPINE1) (microglia); thrombospondin 1 (THBS1, in newly formed oligodendrocytes); and integrin subunit alpha M (ITGAM) and THBS1 (OPC) in patients with stromal infiltration [243]. B7-H3 is upregulated in IDH1-WT gliomas within the immune checkpoint family, particularly in the mesenchymal subtype. Fusion gene analysis reveals strong positive correlations between B7-H3 and inducible T cell costimulator (ICOS), PD-1, TIM-3, LAG3, and IDO [244]. PTX3, another highly expressed protein in GBM, is also correlated with poorer survival in Zhang et al.'s list and is closely related to TIM-3, PD-1/PD-L1, and B7-H3 expression in the GBM TME [245]. According to the results of Gene Ontology (GO) analysis, Kaplan–Meier (K-M) survival analysis, and Pearson correlation analysis, CD163 expression is positively correlated with the malignancy of gliomas, especially in IDH1-WT GBM and mesenchymal subtypes. It is closely related to immune checkpoint markers (B7-H4, B7-H3, LAG3, TIM-3, and PD-1/PD-L1) and other macrophage markers arginase 1 (ARG1), TGF-β, IL-10, and IL-6 [246].

Recently, using single-cell sequencing results for classifying cell components in the GBM microenvironment and predicting patient prognosis and treatment responsiveness through immune scoring based on bioinformatics analysis has gained prominence [247]. Diverse classification results provide researchers and clinicians with a range of evaluation criteria to address the high heterogeneity of GBM treatment. In a study by Yang et al.[248], scoring small nucleolar RNA host genes (SNHGs) revealed that GBMs with high SNHG scores are connected with a poorer prognosis, a greater incidence of the mesenchymal subtype, and increased infiltration of immunosuppressive cells. Further analysis indicated that high SNHG scores correlate with a weakened reaction to anti-PD-1/PD-L1 immunotherapy. High SNHG scores were observed to be more sensitive to targeting EGFR or ERK-MAPK pathways in tumors. MyD88 is a critical adaptor protein in the Toll-like receptor (TLR)/MyD88/NF-κB pathway [249]. In GBM, especially in the mesenchymal subtype, MyD88 is most highly expressed and negatively correlated with PD-1 expression. Patients with high MyD88 expression exhibit an increased immune phenotype score (IPS) [250], and similar results are observed in subsets of PD-1^+^/CTLA-4^−^ treatment and PD-1^+^/CTLA-4^+^ treatment [251]. The mRNA stemness index (mRNAsi) reflects the gene expression characteristics of cancer stem cells (CSCs) [252]. Moreover, TNF alpha-induced protein 8 like 2 (TNFAIP8L2) is an emerging immune checkpoint biomarker that may be a potential target for immunotherapy. Immune cell infiltration and stemness feature analysis showed a significant correlation between TNFAIP8L2 and the CSC index in GSCs, and high TNFAIP8L2 expression decreases macrophage and DC infiltration by promoting M2 macrophage and Treg approach [253]. The Tumor-Infiltrating Immune Cells-related lncRNA screening framework (TIIClnc), developed based on machine learning principles, can predict the response to immunotherapy by assessing immune cell infiltration levels. Moreover, TIIClnc positively relates to the expression of PD-1/PD-L1 and CD8 while providing better predictive accuracy [254]. Patients with a pathological diagnosis of GBM were exclusively considered. The results depicted in the heatmap also illustrate the heterogeneity of gene expression within GBM to a certain extent, showcasing differences in expression among different patients [255].

Indeed, while omics technologies offer a wealth of information for target prediction, the sheer volume of data can be overwhelming. It is essential to recognize that genes exhibiting differences in the transcriptome may experience altered expression in response to changes in the TME. A lack of consistency and the presence of numerous prediction scoring systems can impact the accuracy of clinical applications. Consequently, the validation of these prediction insights through multiomics technologies and fundamental experimental research becomes imperative. This ensures a full-scale comprehension of the function of genes and enhances the reliability of predictions made from transcriptomic variances in diverse contexts.

### Metabolism regulates the immune response in glioblastoma

Based on existing studies on GBM, it has been demonstrated that metabolites play a crucial role in the onset and progression of GBM. Particularly, previous treatment approaches that categorize GBM based on IDH mutation status have shown promising outcomes for patients. Various types of metabolites serve as a double-edged sword in the pathogenesis of GBM. Therefore, this section will provide a brief overview of three key metabolites: glucose, fat, and proteins (amino acids). Metabolites implicated in distinct cellular processes and functions will be delineated separately in the subsequent discussion.

#### Classical glucose metabolism states in glioblastoma

The Warburg effect is a key metabolic aberration in cancer, including GBM [256]. The Warburg effect denotes the phenomenon wherein tumor cells predominantly depend on aerobic glycolysis for their metabolic needs in the presence of ample nutrients. This deviation from normal physiological processes assists tumor cells in acquiring a swift energy supply, facilitating their rapid proliferation and invasive capabilities [257]. There has been significant interest in the metabolic products of the glycolytic pathway, and therapeutic strategies have primarily targeted these products. However, recent research has indicated that, in addition to the glycolytic pathway, other metabolites, including fatty acids and amino acids, also play regulatory roles in the onset and progression of GBM through existing pathways [258].

In GBM, the influence of IDH1-mutant on epigenetics has gained recognition. D-2HG [259] is one of the earliest known metabolites, and its role in tumor cells is well understood. Recent findings indicate that D-2HG in the microenvironment of GBM can be absorbed by CD8^+^ T cells and target lactate dehydrogenase (LDH), reducing the NAD^+^/NADH ratio in CD8^+^ T cells and resulting in diminished cytotoxicity and impaired interferon-gamma signaling. These characteristics have been validated in clinical samples from IDH1-mutant glioma patients [260]. Another glycolytic metabolite, lactate, functions as an upstream regulator and can be modulated by a micropeptide called MP31, which is encoded in the 5' UTR region of protein tyrosine phosphatase (PTEN). MP31 binds to LDH in mitochondria, inhibiting the conversion of lactate to pyruvate, inducing lysosomal alkalization, inhibiting lysosomal function, and impeding the fusion of lysosomes with mitochondria [239]. Additionally, MP31 enhances GBM cell sensitivity to TMZ by inhibiting the protective mechanism of mitochondria [261].

#### Classical fat and amino acid metabolism states in glioblastoma

Fatty acid (FA) metabolism, primarily mediated by fatty acid oxidation (FAO), contributes to immune suppression in GBM [239]. Various FA transport proteins in Tregs are notably elevated in GBM [262]. Inhibiting FA transport or FAO processes, particularly through the FA transport protein CD36, can reduce Treg-mediated immune suppression, resulting in a significant survival benefit in tumor-bearing mice [263]. Additionally, DHHC9, a key transferase involved in S-acylation and lipidation [264], promotes GBM onset, development, and glycolysis by palmitoylating GLUT1. Elevated DHHC9 levels are connected with poor prognosis in GBM patients [62]. Amino acid metabolism, particularly tryptophan metabolism, regulated by aryl hydrocarbon receptor (AHR), influences the immunosuppressive microenvironment in GBM [265]. The tryptophan metabolite kynurenine promotes MDSCs infiltration by binding to AHR and acting as a transcription factor [266], resulting in decreased CD8^+^ T cell infiltration [267]. Kynurenine binding to AHR induces Treg differentiation and inhibits CD8^+^ T cell function in coculture with dendritic cells and naïve T cells [268]. Furthermore, kynurenine stimulates AHR in TAMs, promoting the expression levels of the chemokine receptor C–C motif chemokine receptor 2 (CCR2) and increasing MDSCs recruitment via the CCR2-CCL2 (C–C motif chemokine ligand 2) axis [121]. Consequently, kynurenine primarily modulates the functions of various immune cells through AHR signaling, inducing an immunosuppressive microenvironment and ultimately promoting GBM progression.

These findings underscore the intricate interplay of metabolic regulations in the functional reprogramming of GBM. The dynamic and complex nature of this interaction enhances our understanding of GBM's high heterogeneity and opens avenues for discovering new therapeutic targets. Indeed, it is essential to acknowledge that metabolites exert effects not only on tumor cells but also on normal tissues. Consequently, selecting appropriate metabolite targets to specifically target tumor cells while sparing normal cells is a critical consideration. This necessitates thorough deliberation to minimize potential off-target effects and maximize therapeutic efficacy.

### GBM-TME crosstalk

TME of GBM encompasses elements from both the tumor niche and the tumor bioenvironment, exhibiting high dynamism and complexity. It comprises a diverse array of immune cells, primarily myeloid cells and microglias, along with blood vessels, extracellular matrix (ECM), and components of the CNS, including neurons and glial cells. This composition varies across different regions of the tumor [269, 270]. Notably, GSCs represent a prominent component with distinctive characteristics [271]. Recent ST and scRNA-seq analyses affirm the pervasive presence of GSCs [272], highlighting their status as a cellular functional state rather than a discrete cell cluster [273, 274]. GSCs exhibit a dynamic interplay with GBM cells, contributing to the development of therapeutic resistance. They secrete chemokines and pro-angiogenic factors that foster ECs proliferation and recruit immunosuppressive cells, particularly macrophages, forming immunosuppressive phenotypes [275–277]. Another critical feature is the GBM-associated vascular niche, which facilitates oxygen and nutrient supply to the highly vascularized tumor [278, 279]. Together with the BBB, it constitutes a protective physical microenvironment in GBM, influencing drug resistance, recurrence, and invasion [40, 41]. The collaborative actions of tumor cells, stromal cells, and proinflammatory cells act a pivotal role in formatting the new vessels in GBM, leading to vessel distortion or leakage. This phenomenon contributes to tumor cell growth, invasion, and the release of chemokines [280]. Another crucial set of microenvironmental components contributing to the formation of the microenvironment in GBM is the GBM-associated matrix microenvironment. This component encompasses GBM-associated stromal cells (GASCs), which exhibit similar phenotype and function to mesenchymal stem cells (MSCs) and cancer-associated fibroblasts (CAFs) [281]. GASCs may originate from the reverse differentiation from some brain cells (such as ECs, astrocytes, perivascular cells, or vascular smooth muscle cells) or bone marrow-derived MSCs [282]. GASCs play a component in promoting angiogenesis and tumor development within the GBM microenvironment [283], showing a negative correlation with GBM prognosis [284]. Another matrix microenvironment component is the ECM, which undergoes dynamic changes and manifests spatial heterogeneity during GBM development [285], thereby facilitating GBM invasion and influencing the plasticity of local microenvironment components [286]. Recent reports have highlighted the interaction between GBM and neurons [287]. GBM growth driven by neuronal activity can be regulated by some factors such as synaptic adhesion molecule neuroligin-3, brain-derived neurotrophic factor (BDNF) [288] or through neurotransmitter receptors like glutamatergic excitatory synapses (interacting with astrocytes) [287, 289, 290], dopaminergic receptors (D2 and D4 subtypes) [291], and γ-aminobutyric acid (GABA) receptors [292, 293]. In summary, TME is a pivotal participant and target for therapy in tumor development. A comprehensive understanding of the diverse components involved in cells and molecules in the GBM microenvironment and their crosstalk is essential for developing a more effective treatment strategy. Within the immune components, this fraction significantly contributes to the distinctive immunosuppressive milieu of GBM. Therefore, a brief description is given above, and a detailed exploration of the immune components will be provided in the subsequent discussion.

**Fig. 3:**
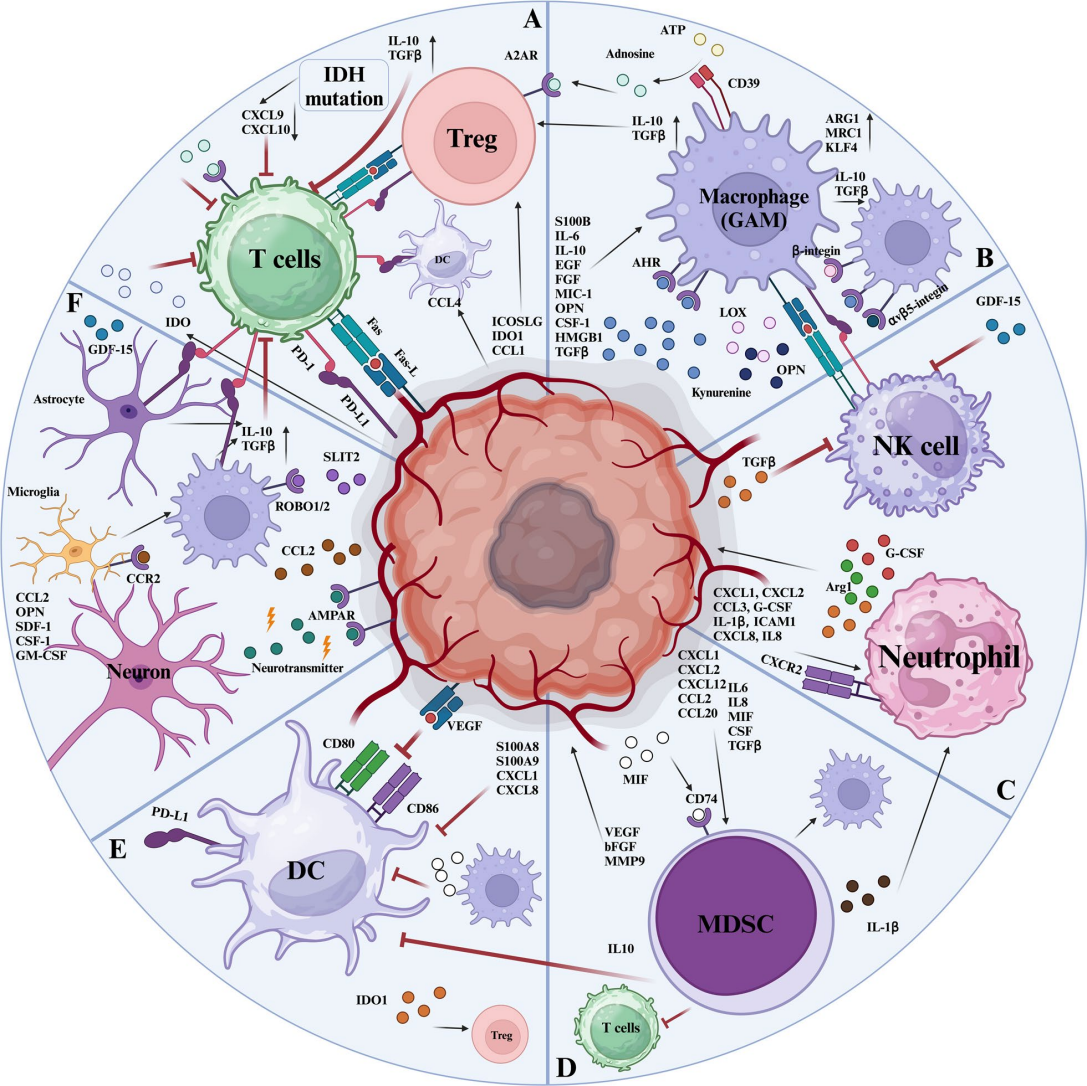
Interactions between GBM and cellular components of the TME. The TME, which includes cells and the ECM, is essential for the initiation and progression of GBM. The formation of a GBM immunosuppressive microenvironment mainly depends on the connection between GBM cells and multifarious stromal cells through different metabolites, cytokines, and signaling pathways, forming a huge hybrid immunosuppressive network. The mechanism of immunosuppression is extremely intricate, so eliminating tumors by a single targeted therapy is incredibly difficult. *GAM* Glioma-associated macrophage/microglia; *AHR* Aryl hydrocarbon receptor

GBM is susceptible to high infiltration by immune cells in the TME [294]. Predominant among these immune populations are myeloid cells, encompassing TAMs (this section refers to GAMs), MDSCs, and neutrophils. Additionally, nonimmune-associated cells, such as neurons, assume a crucial component in GBM progression [295]. There is mounting evidence suggesting that these stromal cells infiltrating into TME foster the growth of GBM and orchestrate the immunosuppressive microenvironment, conferring resistance to immune therapies, including immune checkpoint inhibitors (ICIs) [296]. Following infiltration into the TME, tumor cells manipulate these stromal cells, promoting tumor progression, suppressing anti-tumor immunity, and instigating resistance to immunotherapy [297, 298]. In summary, these discoveries significantly enhance our comprehension of the intricate interplay between cancer cells and stromal cells in the GBM microenvironment (Fig. 3).

#### Crosstalk between glioblastoma and myeloid lineage cells

The interaction between GAMs and GBM represents a prevalent phenomenon within the TME, given that GAMs occupy the largest proportion of all cells [299]. GAMs within GBM comprise brain-resident microglia and bone marrow-derived macrophages, originating from embryonic yolk sac and bone marrow progenitor cells, respectively [300]. Morphologically, microglia are characterized as highly branched quiescent cells with a larger size, whereas macrophages exhibit superior migratory ability, reduced branching, and smaller size [301]. The distribution of these cell types varies dynamically among different tumors. For instance, in GBM, microglia are more infiltrated and widespread, while the core of metastatic brain tumors lacks microglia and is instead populated by macrophages [294, 302]. scRNA-seq analysis provides further insights into this heterogeneity. Moreover, the composition ratio of GAM differs between primary GBM (pGBM) and recurrent GBM (rGBM), with microglia predominant in pGBM and macrophages more prevalent in rGBM [303]. Genetic mutations, such as the classical IDH1-mutant, can alter this ratio, resulting in an abundance of microglia and fewer macrophages in the early stages of IDH1-mutant GBM compared to IDH-WT tumors. However, during tumor progression, macrophage infiltration increases in the IDH-mutant mouse model compared with the IDH-WT mouse model [304]. Additionally, the functional characterization of GAM is a rapidly advancing field. The conventional classification of pro-inflammatory M1 and anti-inflammatory M2 proves overly simplistic for the intricate GBM microenvironment. Current classifications, informed by scRNA-seq, reveal that GAM may exist in a continuous or poorly differentiated state, co-expressing genes characteristic of both M1 and M2 phenotypes, exhibiting high plasticity with dynamic changes [298, 305].

GAMs can induce the transformation of GBM cells into a MES-like status. Oncostatin M (OSM), which originates from GAMs, activates STAT3 through its interaction with oncostatin M receptor (OSMR) or leukemia inhibitory factor (LIF) receptor (LIFR) subunit alpha and with GP130 on GBM cells, prompting the transformation of GBM cells into mesenchymal subtypes in vitro and in vivo [306, 307]. In recent years, in GBM, the significance of circadian locomotor output cycles kaput (CLOCK) transcriptomics has been acknowledged [308]. Elevated CLOCK expression in GBM facilitates the recruitment of GAMs, shaping an immunosuppressive TME through the up-regulation of olfactomedin-like 3 (OLFML3) [69]. CLOCK regulates the legumain (LGMN) signal by forming a complex with brain and muscle ARNT-like 1 (BMAL1), promoting immunosuppressive microglias infiltration and resulting in a poor prognosis. Inhibiting the CLOCK-OLFML3-HIF-1α-LGMN-CD162 axis demonstrates the potential to reduce microglial infiltration, enhance the infiltration, activation, and cytotoxicity of CD8^+^ T cells, and exhibit synergistic effects with anti-PD-1 therapy [309]. GAMs strategically position themselves close to GBM-associated ECs and participate in vascular endothelial growth factor (VEGF)-induced GAMs polarization [310, 311]. Within the microenvironment of GBM, ECs have been identified as a primary source of IL-6. Both IL-6 and CSF-1 induce elevated expression of ARG1 and selective activation of GAMs [312], mediated by hypoxia-inducible factor 2α (HIF-2α) transcription, which induced by peroxisome proliferator-activated receptor γ (PPARγ) [313]. So, targeting EC-derived IL-6 is an effective and potential treatment in GBM [310]. M2 macrophages exhibit high expression of integrin αvβ5 (ITGαvβ5), which supports their phenotypic maintenance and contributes to the immunosuppressive microenvironment. Osteopontin (OPN), secreted by GBM cells, acts as the primary ligand for ITGαvβ5. Deleting OPN reduces M2 macrophage infiltration, enhances GBM cell sensitivity to CD8^+^ T cell cytotoxicity, and improves survival in mouse models [147]. ITGαvβ3 drives M2 macrophage polarization and abnormal angiogenesis in GBM through the Src-PI3K-YAP signaling pathway [314]. Slit guidance ligand 2 (SLIT2) activates and promotes the chemoattraction and polarization of GAMs via the phosphoinositide-3 kinase-γ (PI3K-γ) pathway, mediating GBM immune suppression and abnormal angiogenesis [100]. EZH2 inhibition results in increased M1 marker expression and reduced M2 markers in microglia, decreasing the number of CD206^+^ PB MON-derived macrophages and enhancing microglial phagocytic ability [73]. TIM-3, a common co-inhibitory immune checkpoint in GBM, regulates GBM cell malignancy and induces macrophage migration and polarization toward an anti-inflammatory or pro-tumor phenotype through the IL-6 pathway [33]. In GBM metabolism associated with GAMs, the metabolite lactate from GBM can regulate GAM polarization [59], and exposure to lactate promotes an up-regulation in M2 phenotype markers and decreasing inducible nitric oxide synthase (iNOS) levels, inducing GBM immune escape. High levels of lactate in the GBM TME upregulate the sonic hedgehog (SHH) signaling pathway and facilitate the insulin-like growth factor-binding protein 6 (IGFBP6) expression in microglia, influencing microglial polarization [315]. C-X-C motif chemokine receptor 4 (CXCR4) signaling promotes MET within GBM and shortens survival. DExH-box helicase 9 (DHX9) can enhance macrophage infiltration and polarize them into M2 GAMs in GBM [316]. Silencing DHX9 reduces CSF-1 expression, restoring the inhibitory effect of targeting transcription factor 12 (TCF12) on malignant progression and TAM infiltration in GBM [317]. Overexpression of bradykinin receptor 1 (B1R) and IL-1β promotes vascular cell adhesion molecule 1 (VCAM-1) and cell adhesion molecules intercellular adhesion molecule 1 (ICAM-1) expression, enhancing migratory and adhesive abilities of GBM cells [318]. B1R also contributes to the pro-tumor chemokines and cytokines secretion, like CCL5, IL-6, CXCL11, and IL-8, in GBM, promoting MON infiltration into the TME [319].

In addition to interactions with GAMs, GBM engages with various immune cells, including neutrophils, DCs, NK cells, and MDSCs. Neutrophil infiltration in GBM begins early and persists throughout tumor progression. In vivo experiments suggest that early-infiltrating neutrophils may initially inhibit tumor progression, but this function is lost as tumors progress, leading to a pro-tumor functional phenotype, particularly in tumor protein P53 (TP53)-induced GBM [320]. Ligands of galectin 9 (LGALS9) can bind to TIM-3 receptors on DCs in the cerebrospinal fluid (CSF), inhibiting antigen recognition and presentation. This results in anti-tumor immune response failure mediated through T cells. Blocking exosomal LGALS9 allows sustained tumor antigen presentation and durable anti-tumor immune activity in GBM [321]. Annexin A1 (ANXA1) is implicated in DC maturation and is related to worse outcomes in patients with GBM [322]. Silencing cytokine-inducible SH2 (CIS) containing protein in NK cells increases production levels of IFN-γ and TNF-α, enhancing cancer cells apoptosis mediated by allogeneic NK cells and improving overall survival in mice with GBM [323]. GBM cells can secrete LDH5, which induces natural-killer group 2 member D (NKG2D) ligands upregulation, leading to NKG2D downregulation in NK cells [196]. Leukocyte immunoglobulin-like receptor subfamily B member 2 (LILRB2) promotes MDSCs formation and expansion, prohibiting CD8^+^ T cells from normal function through exosomes, creating an immunosuppressive TME [324]. CXCL1/2/3 secreted by GBM cells and CXCR2 expressed by polymorphonuclear myeloid-derived suppressor cells (PMN-MDSCs) create an axis that regulates PMN-MDSCs output from the bone marrow, resulting in a significant up-regulation in PMN-MDSCs in GBM-draining lymph nodes and spleen [122, 325]. Further details about these interactions are available in Table 2 for the involved cell types.

#### Interaction between glioblastoma cells and T cells

Exhaustion of CD8^+^ T cells and Tregs infiltration act as key components in the immunosuppressive TME within GBM [326]. Transcriptome changes, epigenetic alterations, and the inhibition of certain stromal cells in GBM often contribute to functional impairments in CD8^+^ T cells, leading to a decline in their anti-tumor capabilities. Within the tumor immunosuppressive microenvironment of GBM, T cell function is adversely affected by cytokines and metabolites and is directly inhibited by tumor cells, Tregs, GAMs, and MDSCs. These inhibitory effects are primarily mediated through the surface receptors of these immune cells [327].

scRNA-seq results have highlighted that S100A4 is important in regulating Tregs and bone marrow-derived cells in GBM. Increased expression of S100A4 in Treg cells is related to worse outcomes in patients with GBM [328]. GPNMB is predominantly expressed on macrophages in GBM. Macrophages with high levels of GPNMB induce MET in tumor cells and inhibit T-cell activation, fostering an immunosuppressive microenvironment. Targeting glycoprotein nonmetastatic melanoma protein B (GPNMB) could enhance tumor sensitivity to molecularly targeted therapies and create a more favorable environment for immune responses from T cells [329]. Moreover, the immune checkpoint TIM-3 has been identified as an inhibitor of microglia and CD8^+^ T cell function, playing a critical role in GBM cell proliferation and tumorigenesis. Targeting TIM-3 upregulates the presence of NK cells, DCs, CD8^+^ T cells, and microglias characterized by proliferative and active phenotypes. An upregulation of the secretion of immune-stimulating factors such as IFN-γ, CLL2, IL-1β, CCL5, and CXCL10 into the TME of GBM accompanies this. Ultimately, TIM-3 blockade could induce profound pro-inflammatory changes in the TME, inducing T-cell activation and generating immune memory, thereby inhibiting the recurrence of tumor [32]. The overexpression of common immune checkpoint molecules in the GBM microenvironment can also impact T cell function (Table 2 and Fig. 3).

#### Interaction between glioblastoma cells and neurons

Recent research has underscored the growing recognition of the nervous system as a crucial regulator of cancer, as it plays a role in various stages, from tumorigenesis to malignant growth and metastatic spread. In the context of GBM, this relationship is bidirectional. Not only does the nervous system regulate GBM progression, but GBM also can remodel and hijack the nervous system, affecting its structure and function [330]. Interactions between the nervous system and GBM extend beyond the local TME, influencing systemic processes. Neurons and glial cells, which support the CNS, impact the function and infiltration of immune cells by releasing paracrine factors. This intricate interplay between the nervous system and GBM adds an extra aspect of complicity to understanding the TME and its impact on cancer progression [331].

The relationship between sensory stimuli and the development or progression of brain tumors, including GBM, is an intriguing area of research [332]. Reports suggest that sensory signals, such as visual or olfactory stimulation, may influence the development and behavior of brain tumors, potentially through signaling pathways such as mammalian target of rapamycin (mTOR) signal [333]. For instance, visual stimulation has been linked to the development of optic nerve gliomas in mice with specific gene mutations. Similarly, olfactory stimulation has been associated with promoting GBM, and this effect has been attributed to mTOR signal. The mTOR signal is a crucial regulator of cellular processes, including cell growth and proliferation. The mTOR signal in the context of GBM may also impact the immune microenvironment. Activation of mTOR signal promotes the immunosuppressive microglial formation by regulating the activity of the transcription factors STAT3 and NF-κB. This, in turn, hinders the T-cell proliferation and immune response, allowing GBM cells to escape from the anti-tumor immunity as well as facilitating the growth of tumors in experimental models [334]. Susan et al. [335] explored the potential therapeutic implications of targeting mTOR in the context of GBM. Inhibition of the mTOR pathway, such as rapamycin (RAPA), has been investigated to reinduce anti-tumor immune activity. Using RAPA in a training method related to taste-immune association learning demonstrated the ability to reinstate a proinflammatory, anti-tumor TME. This approach has shown promising outcomes in animal models, suggesting that modulating mTOR signal is a potential method to enhance anti-tumor immunity in GBM.

The intricate interplay of GBM and its microenvironment adds another layer of complexity to understanding and treating this aggressive brain tumor. The high degree of intratumor heterogeneity in GBM, coupled with rapid lineage switching, is rooted in its permissive epigenetic and transcriptomic landscape. One fascinating aspect is GBM's ability to mimic the transcriptomic state of normal neuronal populations, a strategy employed to evade immune attacks by imitating the developmental trajectory of normal neurons [25, 336, 337]. Efforts to limit GBM plasticity within these neural-like pathways are advanced to enhance the validity in targeting tumor heterogeneity [338]. Despite genetic mutations, the transcriptional signature of GBM cells tends to converge on similar neural-like states. However, significant differences exist between the core and edge of GBM, highlighting distinct biological properties. Notably, immune infiltration-related injury programs dominate this phenomenon, leading to the generation of hyperproliferative injured neural progenitor cells (iNPCs). iNPCs constitute a substantial proportion of resting GBM cells and can be activated by interferon within the T cell niche [339]. The microenvironment at the immuno-cold edge of the tumor appears to influence GBM's trajectory, resembling normal neuronal development. This environment prompts the differentiation of tumor cells into aggressive AC-like cells [340]. These findings underscore the crucial role of local components within the TME in shaping the fate of GBM cells. Understanding and potentially manipulating these interactions could offer new avenues for therapeutic interventions aimed at targeting specific cellular states and enhancing treatment outcomes in GBM patients.

The complex interactions among CNS, GBM, and the immune system highlight the complex nature of this disease. The regulatory crosstalk between these systems influences the delicate balance between pro-tumor inflammation and anticancer immunity. Understanding these interactions necessitates an interdisciplinary approach, bringing together expertise from neuroscience, developmental biology, immunology, and cancer biology. Collaboration across these diverse fields is crucial for unraveling the complexities of GBM and developing targeted therapeutic strategies. Insights gained from this interdisciplinary collaboration could pave the way for innovative approaches that disrupt the regulatory pathways exploited by GBM. By leveraging knowledge from multiple disciplines, researchers and clinicians may identify new therapeutic targets, enhance treatment efficacy, and ultimately improve outcomes for individuals affected by GBM.

## The role of MDSCs in the initiation and development of glioblastoma

In this section, we focus exclusively on MDSCs, as their relatively limited representation belies their essential component in initiating and progressing the comprehensive immunosuppressive microenvironment in GBM. This significance extends beyond their direct immunosuppressive functions, encompassing intricate interactions with other stromal cells. Specifically, MDSCs are involved in priming or modulating the functions of additional immunosuppressive cells while concurrently impeding the functions of normal immune components.

MDSCs constitute the significant role in the immunosuppressive TME of GBM and cancer cells' response to immunity. In the GBM microenvironment, GAM emerges as the predominant immunosuppressive component, accounting for up to 50% of all living cells in GBM [341]. However, it is noteworthy that MDSCs (accounting for 4%-8% of all CD45^+^ cells in GBM) [342] primarily mediate the formation of GAMs, and their inhibitory effect surpasses that of GAMs and Tregs. Within the TME, enhanced infiltration of B cells, cytotoxic T cells (CTLs), T cells, and NK cells correlates with a more favorable prognosis. Conversely, heightened infiltration of MDSCs is associated with a poorer prognosis [343–345]. Under pathological conditions, MDSCs function as immunosuppressive regulatory cells originating from the bone marrow [346]. For instance, following infection or in the context of tumors, they accumulate in the PB and tissues [344, 345, 347], a phenomenon not observed under physiological conditions [342]. This accumulation signifies the pathological activation of neutrophils and MONs. MDSCs exert their immunosuppressive effects by inhibiting the release of inflammatory factors and activating immunosuppressive cells, thereby mediating the suppression of the body's anti-tumor immunity [348]. They can be categorized into two types: monocytic myeloid-derived suppressor cells (M-MDSCs) and PMN-MDSCs. These subtypes exhibit distinct phenotypes with unique gene expression profiles yet share certain similarities. PMN-MDSCs, resembling the morphology of neutrophils, predominantly induce long-term immune tolerance. Conversely, M-MDSCs, resembling MONs, tend to polarize into GAMs, playing a rapid immunosuppressive role thereafter [348]. MDSCs are recognized as pivotal components implicated in the immune evasion of tumors. Escalation during the induction and activation of MDSCs can enhance tumor immunosuppression, thereby contributing to tumor progression, encompassing angiogenesis, invasion, and metastasis [349]. Therefore, in the following section, we will elaborate on how MDSCs mediate these processes in GBM and the possible mechanisms.

### Regulatory mechanisms of MDSC origin

**Fig. 4:**
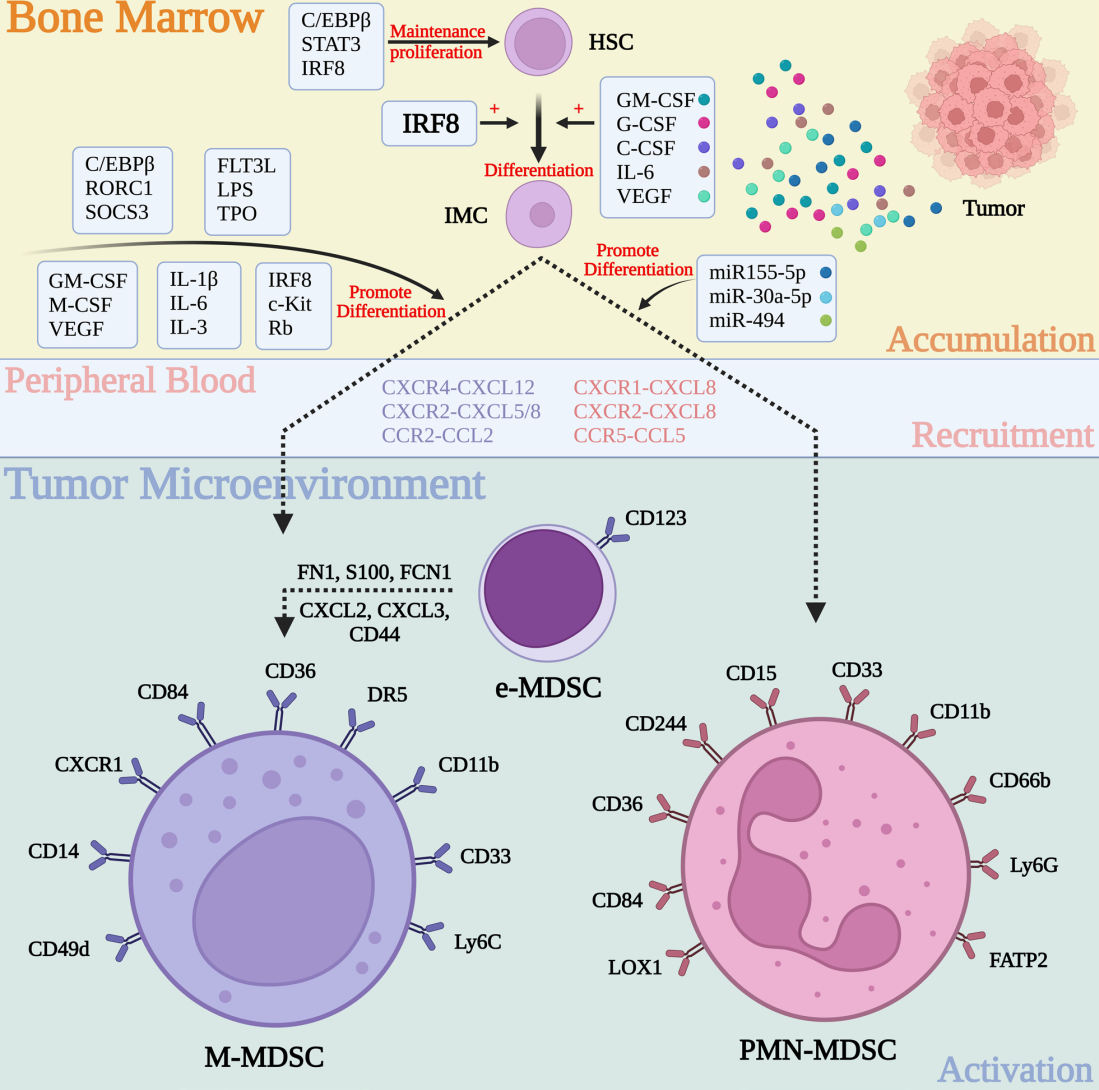
Mechanisms of MDSC generation, recruitment, and activation. HSCs in the BM proliferate and differentiate into IMCs under the stimulation of various signaling pathways, such as the IRF8 signaling pathway. Subsequently, IMCs are recruited and differentiated into MDSCs, including M-MDSCs and PMN-MDSCs, via a variety of chemokines in the PB. Then, MDSCs are activated by a variety of cellular mediators released by tumor cells, thereby exerting immunosuppressive effects and maintaining an immunosuppressive microenvironment. *c-Kit* Receptor tyrosine kinase; *C/EBPβ* CCAAT/enhancer binding protein β; *CSF-1/M-CSF* Macrophage colony-stimulating factor-1; *e-MDSCs* early-stage myeloid-derived suppressor cells; *FATP2* Fatty acid transport protein 2; *FCN1* Ficolin 1; *FLT3L* Fms-related tyrosine kinase 3 ligand; *FN1* Fibronectin 1; *G-CSF* Granulocyte colony-stimulating factor; *GM-CSF* Granulocyte–macrophage colony-stimulating factor; *HSC* Hematopoietic stem cell; *IL* Interleukin; *IMC* Immature myeloid cells; *IRF* Interferon regulatory factor; *LPS* Lipopolysaccharide; *M-MDSCs* Monocytic myeloid-derived suppressor cells; *miRNA* Micro RNA; *PMN-MDSCs* Polymorphonuclear myeloid-derived suppressor cells; *Rb* Retinoblastoma; *RORC1* Receptor-related orphan receptor γ; *SOCS3* Suppressor of cytokine signaling 3; *STAT* Signal transduction and transcription factor; *TPO* Thrombopoietin; *VEGF* Vascular endothelial growth factor

MDSCs predominantly originate from the bone marrow, although their presence is not limited to this site, and they can extend to peripheral lymphoid organs like the liver, spleen, and other tissues [350]. The prevailing theory supporting MDSC genesis is the double signal theory. This theory involves the orchestration of signals through GM-CSF, granulocyte colony-stimulating factor (G-CSF), and CSF-1. These signals activate transcription factors such as STAT3, IRF8, and CCAAT/enhancer binding protein β (C/EBPβ), thereby promoting proliferation within the BM. Pathologically, a downregulation of IRF8 signaling occurs, resulting in immature myeloid cells (IMCs) accumulating in spleen and bone marrow. These IMCs subsequently differentiate into PMN-MDSCs or M-MDSCs upon peripheral activation. Under physiological conditions, PMN-MDSCs or M-MDSCs can further differentiate into DCs, polymorphonuclear neutrophils (PMNs), and MONs [351–355]. Notably, this differentiation lasts longer than normal and exhibits specific expression profiles and characteristics that support tissue angiogenesis and immune cell suppression under pathological conditions [356]. Physiologically, various signals, including endoplasmic reticulum stress (ERS), VEGF, IL-6, macrophage colony-stimulating factor (M-CSF), IL-3, IFN-γ, thrombopoietin (TPO), GM-CSF, receptor tyrosine kinase (c-Kit) ligands, lipopolysaccharide (LPS), FMS-like tyrosine kinase 3 ligands (FLT3L), and IL-1β, with GM-CSF upregulate and mediate the differentiation of MDSCs [299, 346]. A pivotal role in the generation of PMN-MDSCs is ascribed to the downregulation of IRF8 in hematopoietic progenitor cells, as it induces PMN-MDSC generation and participates in STAT3/STAT5-mediated anti-tumor processes (Fig. 4) [299, 357–362].

In non-IRF8-regulated cell populations, granulocyte-monocyte progenitors (MLPGs) can undergo differentiation into PMN-MDSCs through the downregulation of the retinoblastoma gene (Rb) [363]. The crucial transcription factors C/EBPα and C/EBPβ, generated by bone marrow cells, play opposing roles in MDSC generation, where C/EBPβ promotes MDSC generation and C/EBPα inhibits MDSC generation [364], C/EBPβ regulates MDSC generation by controlling GM-CSF and G-CSF, and it also modulates the expression of iNOS, NADPH oxidase 2 (NOX2), and ARG1, influencing the essential functions of MDSCs, particularly M-MDSCs. Additionally, retinoic acid receptor-related orphan receptor γ (RORC1) enhances the expression of C/EBP-β through the SOCS3 and B cell lymphoma 3 (Bcl3), promoting MDSC generation. Furthermore, C/EBPβ can facilitate the differentiation of MDSCs into TAMs [365]. CD33^+^ MDSC-like cells and CD14^+^ PMN-MDSCs promote the aggregation and differentiation of PMN-MDSCs in peripheral blood mononuclear cells (PBMCs) [347, 359, 366].

MDSCs infiltrate the TME under the influence of cytokines or some signaling molecules, promoting the growth and progression of tumors through suppressing the normal anti-tumor immunity [367]. M-CSF, GM-CSF, G-CSF, and other cytokines are important in maintaining metabolic reprogramming, proliferation, and epigenetic modifications in MDSCs. Soluble cell factors, including IL-6, TNF, IL-4, IL-1 family cytokines, and IL-13 [367], not only facilitate the metastasis and invasion of cancer cells but also control MDSCs accumulating and activating in the TME [368, 369]. Consequently, a strong correlation has been established between the aggregation of MDSCs and the invasion of tumor cells in the TME. Among the earliest transcription factors implicated in MDSC generation is the STAT family, including STAT3, STAT5, and STAT6. Notably, STAT3 and its downstream pathways, involving the upregulation of c-Myc, Bcl-xL, Cyclin D, S100A8/A9, and NOX2, along with cooperation with cytokines like IL-6, GM-CSF, and G-CSF, are implicated in MDSC accumulation and immunosuppressive mechanisms [347, 370–372]. Specifically, S100A8/A9 can directly bind to membrane receptors, promoting MDSC migration. Moreover, STAT3 is able to bind with the promoter of ARG1, participating in immunosuppression [373].

Recently, microRNAs (miRNAs) have garnered increasing attention in MDSC development; these molecules play pivotal roles in regulating MDSC proliferation, maturation, and immunosuppressive functions. For instance, miR155-5p, which is induced by TGF-β, inhibits phosphatidylinositol-3,4,5-trisphosphate 5-phosphatase 1 (SHIP-1) and promotes STAT3 activation, thereby supporting MDSC proliferation and differentiation [374]. Similarly, miR-30a-5p facilitates the activation of MDSC by targeting SOCS3 downstream of the JAK/STAT3 pathway, encouraging the production of IL-10, ARG1, and reactive oxygen species (ROS) [375]. Furthermore, miR-494 downregulates the expression of PTEN, promotes the PI3K/Akt signal, and modulates the accumulation of MDSC [376]. Additionally, miR-21-5p, miR-223-3p, and others have been implicated in MDSC development [347, 377].

### The classification of MDSCs in glioblastoma

#### The general classification of MDSCs

As previously mentioned, MDSCs are broadly categorized as two main clusters: PMN-MDSCs and M-MDSCs. PMN-MDSCs emerge early in the PB or peripheral lymphoid organs of individuals with tumors, potentially representing an early stage of MDSC development. Notably, they possess migratory capabilities and constitute over 75% of MDSCs, playing a crucial role in the expansion of MDSC populations and their migration to and residence within tumor tissues [352, 361]. Conversely, TAMs can differentiate from M-MDSCs within the microenvironment and exhibit more pronounced immunosuppressive effects than PMN-MDSCs [359, 378].

Early-stage MDSCs (e-MDSCs) represent a newly recognized third subtype of suppressive MDSCs. These cells have been identified as bone marrow cells lacking markers for mature MONs or neutrophils in both the PB and the TME. Classified as immature MDSCs due to the absence of mature lineage markers, it remains to be established whether they serve as precursors for other MDSC subsets [379]. In vitro experiments have indicated that e-MDSCs may exhibit the lowest suppressive capacity in the TME [380], demonstrating the weakest ability to restrain T cell proliferation. Unlike other MDSCs, the accumulation of e-MDSCs does not appear to be correlated with overall survival in cancer patients [380, 381]. Ongoing research explores markers associated with eosinophilic granulocytes, such as the high expression of CD123, as potential identifiers for e-MDSCs [382]. Recent findings from scRNA-seq suggest that markers such as CD14, CD15, and CD16 may also be useful for identifying e-MDSCs [383]. For GBM, e-MDSC is a unique subset of MDSCs present in it, which is hardly observed in other grades of glioma [383].

#### The molecular classification of MDSC

In the early stages of molecular studies in mice, CD11b and Gr1 were utilized for labeling MDSCs, with different Ly6G and Ly6C expressions used to classify PMN-MDSCs and M-MDSCs: Ly6G^+^/Ly6C^lo^/CD11b^+^ for PMN-MDSCs and Ly6G^−^/Ly6C^hi^/CD11b^+^ for M-MDSCs [343]. Currently, CD49d is considered a specific marker for M-MDSCs [384], while lectin-like oxidized low-density lipoprotein receptor 1 (LOX1) is becoming a novel specific marker for PMN-MDSCs [299]. In humans, PMN-MDSCs are markered with CD14^−^/CD11b^+^/CD66b^+^/CD15^+^, while M-MDSCs are markered with HLA^−^/DR^−/low^/CD11b^+^/CD15^−^/CD14^+^ (Fig. 4) [343]. In the context of GBM, vascular noninflammatory molecule 2 (VNN2^+^) may serve as a unique marker for MDSCs [385].

In recent scRNA-seq studies of GBM, the role of e-MDSCs has gained gradual recognition. e-MDSCs interact with GSCs and contribute significantly to the transformation of tumors into more malignant mesenchymal types, correlating with a poor prognosis [383]. scRNA-seq has identified two distinct types of GBM: e-MDSCs and M-MDSCs. e-MDSCs primarily participate in the immunosuppression process in GBM. Simultaneously, M-MDSCs primarily function as recruits, attracting PMN-MDSCs, TAMs, and Tregs in GBM. Additionally, M-MDSCs are capable of transforming into each other. Under the influence of the extracellular matrix and inflammatory factors (FN1, FLNA, VCAN, CD44, FCN1, CXCL2, S100, CXCL3, etc.), e-MDSCs can transform into M-MDSCs. This transformation leads to an increase in glycolysis-related genes and antioxidant and stress processes associated genes downregulating [383].

### The mechanism of MDSC recruitment in glioblastoma

MDSCs in tumors play a key component in the development of tumors, and tumors can secrete specific chemokines to facilitate the MDSCs’ recruitment. Chemokines such as CXCR4-CXCL12, CXCR2-CXCL5/8, and CCR2-CCL2 [386], with CXCR2-CXCL5, are particularly significant in primarily regulating M-MDSCs’ recruitment [387]. In human colorectal cancer, the expression of CCL2 increases with cancer progression, and CCL2 deficiency has been associated with reduced infiltration of intratumoral MDSCs and smaller tumor sizes in spontaneous mouse models of colon cancer [388]. Similarly, the upregulation of the expression of CCL15 in colorectal cancer can enhance M-MDSCs’ recruitment [389]. PMN-MDSCs’ recruitment is mediated mainly by chemokines such as CXCR1-CXCL8, CXCR2-CXCL8, CCR5-CCL5, CXCL6, and CXCL12 [388, 390, 391]. Additionally, CCL2, CCL3, and hypoxia have been identified as factors contributing to the recruitment of PMN-MDSCs. IL-8 is also considered one of the inducers of MDSC mobilization [367]. In brain metastasis, CXCL10 emerges as a crucial mediator that establishes the premetastatic niche and contributes to immune suppression in brain tumors [392].

Observations from PB and intratumoral studies in glioma patients reveal a notable proliferation of PMN-MDSCs and M-MDSCs in patients with GBM compared to that in healthy individuals’ PB. GBMs are among the tumors exhibiting the highest levels of MDSCs in PB [22]. Within the PB in patients with GBM, PMN-MDSCs emerge as the dominant subset, with M-MDSCs constituting almost the entirety of MDSC subpopulations [393]. In high-grade gliomas (HGGs) with IDH-mutant, intratumoral studies indicate that PMN-MDSCs are the predominant subset [394]. Moreover, the increased percentage of PMN-MDSCs within the tumor may suggest BBB disruption [395], highlighting the heterogeneity of MDSCs and the TME in GBM. Elevated MDSC levels in the PB and increased infiltration of MDSCs in GBM are indicative of a poorer prognosis [342, 396], with the degree of M-MDSC infiltration correlating with glioma grade [396, 397]. Radiomics development has further confirmed the robust correlation between high MDSC infiltration and poor prognosis in gliomas [398]. Notably, in patients with rGBM, the MDSC population in the TME does not significantly differ from that observed before treatment. This indicates that the persistence of MDSCs is essential in the rGBM [399, 400].

In the GBM TME, numerous constituents contribute to tumor progression, particularly influencing MDSCs. For instance, GBM cells can secrete IL-8, resulting in the upregulation of CCR2 [401]. CCR2 has dual functions, not only facilitating the recruitment of MDSCs but also activating MDSCs within the TME of GBM [396, 402]. GSCs are proficient in secreting substantial amounts of macrophage migration inhibitory factor (MIF) [112], thereby augmenting the production of ARG1 through a CXCR2-dependent pathway, consequently impeding immune function [403]. Notably, while inhibiting MIF does not directly impede tumor progression, it diminishes the infiltration of MDSCs, underscoring its specificity in targeting MDSCs in GBM [403]. Furthermore, GBM cells secrete galectin-1, eliciting stimulation of tumor angiogenesis. Recent investigations have demonstrated that inhibiting galectin-1 significantly diminishes MDSCs’ amount in the microenvironment and improves the mice with GBM in prognosis [404], a phenomenon potentially linked to the regulation of LGALS1 [405]. The histone methyltransferase G9a is pivotal in the GSC-mediated tumor immune microenvironment (TIME). It upregulates the Notch pathway by binding to the H3K9me2 modification on the promoter of F-box and WD-40 domain protein 7 (Fbxw7), which can suppress Notch signal, thereby fostering the recruitment of MDSCs in GBM [406]. FGL2 in GBM exhibits a positive correlation with the increase of MDSCs, notwithstanding its lack of association with the conventional upregulation of PD-1 or CD39 [407]. Notably, activation of the Notch pathway in GBM induces upregulation of CCL2, thereby promoting the recruitment of MDSCs [408]. In addition to the IDO mechanism, the upregulation of complement factor H (CFH) or FH-like protein 1 (FHL-1) can similarly facilitate the infiltration of intratumoral MDSCs in GBM [136].

LOX1 is recognized as a distinctive marker for PMN-MDSCs, playing a vital component in suppressing T-cell proliferation within GBM and contributing to early recurrence and progression [409]. Recent investigations specifically focusing on GBM with epidermal growth factor receptor variant III (EGFRvIII) mutations have uncovered an increasing abundance of PMN-MDSCs, correlating with resistance to PD-1 and CTLA-4 inhibitors. Subsequent studies have elucidated the regulatory axis involving CXCL1/2/3 and the CXCR2 receptor expressed by PMN-MDSCs, influencing PMN-MDSCs’ production and recruitment in bone marrow [325]. These findings underscore an intricate interplay among genetic mutations, TME heterogeneity, and resistance to ICIs in GBM. In contrast to PMN-MDSCs, M-MDSCs in GBM manifest heightened expression of integrin β1 and dipeptidyl peptidase-4 (DPP-4). Inhibiting DPP-4 has been shown to diminish tumor migration mediated by the pERK signaling pathway, while targeting integrin β1 eradicates the immunosuppressive phenotype of MDSCs. Notably, the concurrent inhibition of these targets has been shown to enhance survival outcomes in mice bearing GBM [72].

Hence, MDSCs recruited to tumors are influenced by many factors that vary across different cancers, resulting in high variability. Consequently, therapeutic interventions aimed at blocking MDSC recruitment to tumors by targeting a specific chemokine or cytokine may have limited impact. Nonetheless, a potentially more effective approach could involve targeting specific chemokine receptors, as certain receptors can interact with multiple chemokines.

### Immunosuppressive effect of MDSC in glioblastoma

#### The signaling molecular involved in immunosuppression in MDSC

**Fig. 5:**
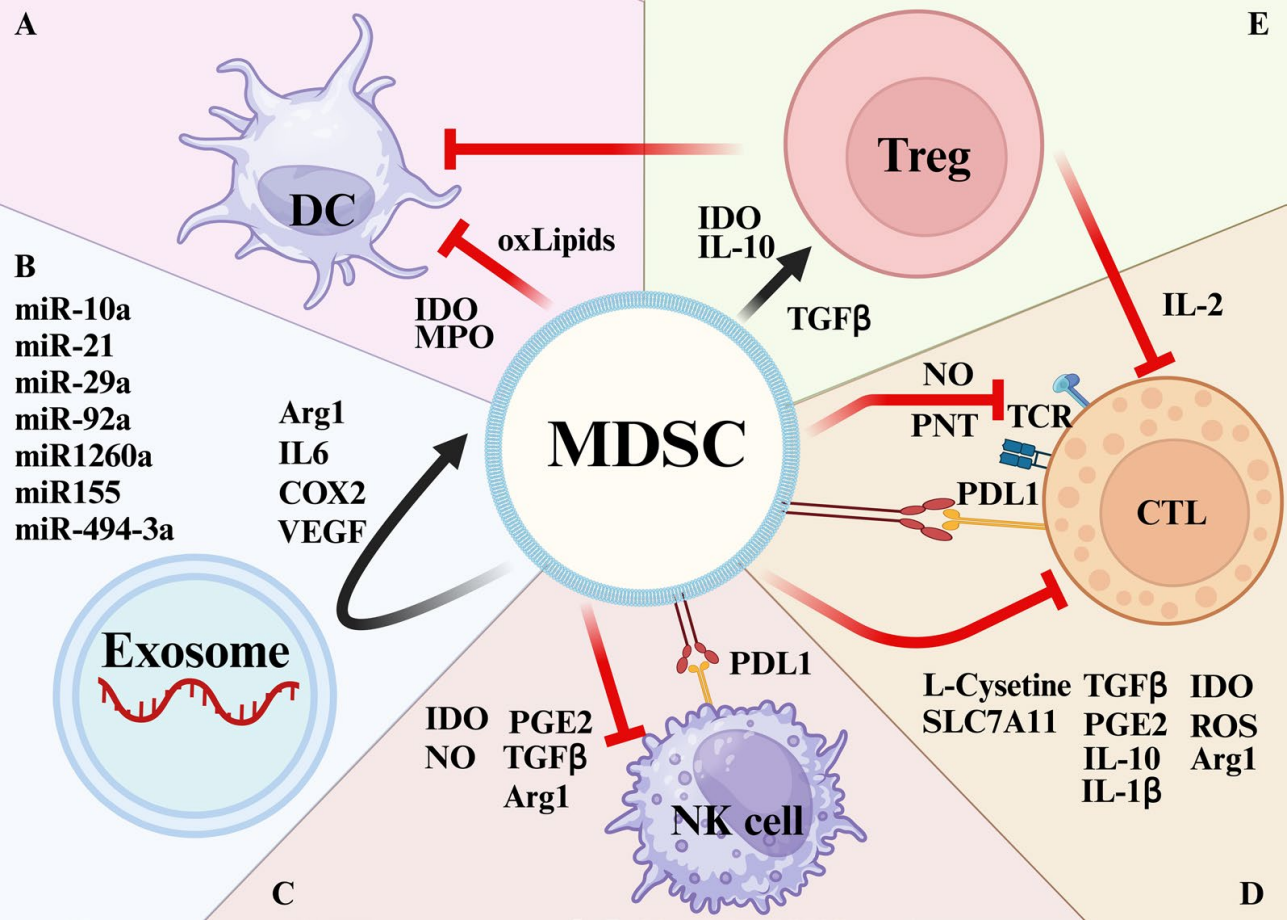
Immunosuppressive role of MDSC in the TME. Once infiltrated into the tumor, MDSCs can promote tumor progression and exert immunosuppressive effects in a variety of ways. Among them, the most important is the release of multiple cytokines to directly inhibit the activity of CTLs and activate and enhance the function of Tregs, directly inhibiting anti-tumor immunity to create a tumor immunosuppression microenvironment. In addition, it can also inhibit the antigen presentation function of DCs and the tumor-killing function of NK cells and enhance autoimmune suppression through the exosome pathway. *Arg1* Arginase 1; *COX2* Cyclooxygenase 2; *CTL* Cytotoxic T cells; *DC* Dendritic cells; *IDO* Indoleamine 2,3-dioxygenase 1; *IL* Interleukin; *MDSC* Myeloid-derived suppressor cell; *miRNA* microRNA; *MPO* Myeloperoxidase; *NK cell* Natural killer cell; *PGE2* Prostaglandin E2; *PNT* Peroxynitrite; *ROS* Reactive oxygen species; *SLC7A11* Solute carrier family 7 member 11; *TGF* Transforming growth factor; *Treg* T regulatory cells; *VEGF* Vascular endothelial growth factor

MDSCs exhibit weaker immunosuppressive abilities than normal bone marrow-derived suppressor cells, yet they exert a prolonged inhibitory effect, leading to sustained immune suppression. The immunosuppressive mechanisms of MDSCs encompass various pathways, including Toll-like receptor signaling [410], certain proinflammatory cytokines (like IL-13, IL-4, PGE2, IFN-γ, and IL-1β) [411], and exosome secretion [412]. Activation of NF-κB signal facilitates iNOS2 expression [358], an essential player inhibiting M-MDSCs’ function. Additionally, ERS is another crucial factor activated by tumor hypoxia, low pH, and proinflammatory cytokines. This activation leads to increased expression of ERS-related proteins (CHOP, LOX1, DR5), IL-6, C/EBPβ, and pSTAT3, further enhancing MDSC recognition and targeting of immune cells in the TME [357, 413, 414]. Notably, ERS has distinct impacts on PMN-MDSCs and M-MDSCs, with inositol-requiring enzyme 1α (IRE1α) and human-activating transcription factor 6 (ATF6) playing critical components in the immunosuppressive activity of PMN-MDSCs. In contrast, M-MDSCs are less dependent on ERS and rely predominantly on IL-6-mediated immunosuppression [358]. Different cytokines exert diverse effects on MDSCs [343]. TNF-α and IFN-γ can promote the formation of a proinflammatory phenotype in the GBM microenvironment by reducing MDSC numbers [415]. This process is activated by JAK/STAT signal, inducing IRF1 downregulation, promoting the secretion of PD-L1, and altering the immunoescape microenvironment [416]. However, the upregulated expression of FAT atypical cadherin 1 (FAT1) enhances IL-1β, IL-10, PD-L1, IL-6, and HIF-1α secretion through AP-1 signal. This promotes the function of MDSCs and establishes a TIME within GBM [417]. Table 3 [72, 112, 122, 324, 354, 388, 401, 406, 409, 418–440] and Fig. 5 provide a comprehensive summary of the main immunosuppressive pathways targeting the TME [441].

**Table 3:** The summary of related targets in MDSCs mediated immunosuppression

Target	Potential role in MDSCs	Effects of targeted therapy	Reference
CCL2	MDSCs inhibit T cell function and promote MDSCs recruitment through STAT3 pathway	Inhibition of MDSCs recruitment	[72]
CCL15	Promote the recruitment of MDSCs	Inhibition of MDSCs recruitment	[112]
CCL26	Induction of MDSCs recruitment under hypoxic conditions	Inhibition of MDSCs recruitment	[122]
CCL9/CCR1	Promote the recruitment of MDSCs	Inhibition of MDSCs recruitment	[324]
CCR2	Promote the recruitment of MDSCs	Inhibits MDSCs recruitment and promotes the effect of ICBs therapy	[354]
CCR4	Promote the recruitment of MDSCs	Inhibition of MDSCs recruitment and inhibition of GBM microglia recruitment	[388]
CCRK	MDSCs recruitment was promoted through indirect activation of STAT3	Inhibition of MDSCs recruitment	[401]
CXCL1/2	Promote MDSCs infiltration and T cell inhibition in GBM	Inhibition of MDSCs recruitment	[406]
CXCL5	Mediate rapid recruitment of MDSCs to tumor sites	Inhibition of MDSCs recruitment	[409]
CXCL12	Inhibit T cell function and mediate tumor metastasis	Promote T cell anti-tumor function	[418]
CXCR1/2	Promote MDSCs recruitment and tumor angiogenesis	Inhibition of MDSCs recruitment	[419]
CD39	Inhibit inflammation in TME	Promote TME inflammation	[420]
DPP-4	Mediate tumor metastasis by pERK signaling	Inhibition of tumor metastasis	[421]
ENTPD2/CD39L1	Induce MDSCs immunosuppressive phenotype under hypoxia	Promote the maturation of MDSCs	[422]
G9a	Promote the recruitment of MDSCs in GBM	Inhibition of MDSCs recruitment	[423]
G-CSF	Continuously inducing the formation of MDSCs	Inhibition of MDSCs formation	[424]
HDAC	Inhibit T cell activity	Promote T cell anti-tumor function	[425]
IL-1β	Mediate differences in the distribution of MDSCs in different sexes	Inhibit the immunosuppressive function of MDSCs in female	[426]
IL-6	Mediate phenotypic changes of MDSCs	Increase tumor sensitivity to chemotherapy	[427]
IL-10	Promot the immunosuppressive phenotype of MDSCs through the autocrine pathway	Inhibit the immunosuppressive phenotype of MDSCs	[428]
IL-12	Mediate the reprogramming of MDSCs function	Influence the immunosuppressive phenotype of MDSCs	[429]
IL-18/TLR2	Promote MDSCs recruitment and inhibit T cell function	MDSCs recruitment was inhibited and T cell function was restored	[430]
IDO1	Promote the immunosuppressive function of MDSCs	Remove the immunosuppressive effect of MDSCs	[431]
LILRB2	Facilitate the transform and recruitment of MDSCs in GBM	Inhibition of MDSCs recruitment	[432]
LOX1	Inhibit T cell proliferation	Promote T cell function	[433]
MIF	Strengthen the function of MDSCs in the TME of GBM	Inhibit the immunosuppressive function of MDSCs	[434]
miR-1246/HIF-1α	Maintain the immunosuppressive function of MDSCs under hypoxic environment	Inhibit the immunosuppressive function of MDSCs	[435]
PD-L1	MDSCs indirectly inhibit the antitumor activity of T cells through PD-L1	Inhibition of MDSCs mediated immunosuppressive environment	[436]
PI3Kγ	Promote the generation of MDSCs	Reduce the generation of MDSCs	[437]
SSAO	Inhibition of MDSC production from PBMC	Inhibition of MDSCs recruitment	[438]
STAT3	Promote the formation and differentiation of MDSCs	The number of MDSCs was decreased by promoting the apoptosis of Fas pathway	[439]
TGFβ	Interfer with IFNγ production and inhibit NK cell activity while apromoting recruitment and expansion of Treg cells	Change the immunosuppressive phenotype of MDSCs	[440]

*DPP-4* Dipeptidyl peptidase-4; *ENTPD2* Ectonucleoside triphosphate diphosphohydrolase 2; *ERK* Extracellular regulated protein kinases; *G-CSF* Granulocyte colony-stimulating factor; *GBM* Glioblastoma; *HDAC* Histone deacetylase; *HIF* Hypoxia-inducible factor; *IDO* Indoleamine2,3-dioxygenase1; *IFN-γ* Interferon γ; *IL* Interleukin; *LILRB2* Leukocyte immunoglobulin-like receptor subfamily B member 2; *LOX1* Lectin-like Oxidized Low-density Lipoprotein Receptor 1; *MDSCs* Myeloid-derived suppressor cells; *MIF* Macrophage migration inhibitory factor; *miRNA* Micro RNA; *NK cells* Natural killer cells; *PI3K* Phosphoinositide-3 kinase; *SSAO* Semi carbazide-sensitive amine oxidase; *STAT* Signal transduction, and transcription factor; *TGF* Transforming growth factor; *TLR* Toll-like receptor; *TME* Tumor microenvironment; *Treg* T regulatory cells

Exosomes, which are double-membrane extracellular vesicles (EVs), play a pivotal role in regulating MDSC function by secreting proteins and miRNAs [442]. Their inhibitory effect on the myeloid cell differentiation is facilitated by TGF-β secretion [443]. Moreover, EVs induce the accumulation of ARG1, cyclooxygenase 2 (COX2), IL-6, and VEGF, thereby enhancing the function of MDSCs [320]. By utilizing heat shock protein 72 (HSP72), EVs activate the TLR2/MyD88 pathway, synergizing with IL-6 to improve the immune inhibitory function of MDSCs [412]. Furthermore, EVs interact with IL-10 and IL-16, participating in microenvironmental regulation, promoting angiogenesis, and activating STAT1/3 to enhance the immunosuppressive function of MDSCs [444, 445]. In GBM, EVs can initiate MDSC differentiation under low-oxygen conditions through retinoic acid related-orphan receptor α (RORα) and PTEN via miR-10a and miR-21, respectively, to promote immune suppression [442]. Another class of miRNAs, miR-92a and miR-29a, can activate MDSCs by targeting high mobility group protein B1 (HMGB1) and cAMP-dependent protein kinase regulatory type I-α (Prkar1a) [446]. Additionally, miR-155, miR-27b, miR-1260a, miR-126-3p, miR-494-3p, miR-320, and miR-494-3p may also be associated with the activation of MDSCs [447, 448].

In GBM, the secretion of EVs involves a unique mechanism in which these vesicles interact with heparan sulfate proteoglycans (HSPGs) and MDSCs, inducing the transformation of MDSCs [441]. This process can be inhibited by heparin, leading to a reduction in the number of MDSCs in GBM [449]. EVs derived from GBM cells can reprogram normal MONs, promoting their differentiation into MDSCs and subsequent suppression of T cell function [449]. Again, heparin can inhibit this reprogramming process and restore T cell function. A recently discovered factor, LILRB2, has been found to propagate between GBM cells through vesicles, inducing the formation, expansion, and shaping of the TIME by promoting MDSCs [324]. EVs secreted under hypoxic conditions have shown an enhanced ability to induce or facilitate the generation and transformation of MDSCs, leading to increased infiltration into the TME and subsequent suppression of immune cell function [450, 451]. Additionally, MDSCs can interact with tumor-associated B cells or regulatory B cell (Breg) cells through EVs, transmitting PD-L1 to regulate B cell function and consequently inhibiting the typical immune function of CD8^+^ T cells, thereby suppressing immune function in GBM [452]. The inhibitory effect of EVs on T cell function is also indirectly mediated through MDSCs [453].

#### Metabolism regulation of immunosuppression through the MDSC in glioblastoma

Immunosuppressive factors such as nitric oxide (NO), ROS, and peroxynitrite (PNT) play crucial roles in the immunosuppressive functional mechanism of MDSCs [454]. NO is a key molecule mediating immunosuppression in MDSCs, especially M-MDSCs, and is primarily metabolized by iNOS in the TME, induced by IL-1β, IFN-γ, and TNF-α, which is included in Th1 cytokines, participating in the inhibition of the IL-2-associated receptor [455]. In PMN-MDSCs, the ROS pathway plays a pivotal role, and ROS is mainly produced by NOX2. Phosphorylation of STAT3 can directly regulate NOX3 and increase ROS production [456]. PMN-MDSCs can generate a substantial amount of ROS by mediating TGF-β, GM-CSF, IL-6, and IL-10, inducing T-cell death [457]. Reactive nitrogen species (RNS) also play a complementary role [458]. PNT serves as another mediator, with MDSCs nitrating amino acids through PNT to form TCR-CD8 nitrate complexes [459]. This interferes with the antigen–antibody recognition process, inhibiting antigen-specific immune activation. PNT can also reduce the efficiency of MHC I binding with peptides on the membrane of cancer cells, nitrating CCL2, STAT1, and Lymphocyte cell-specific protein-tyrosine kinase (LCK) to inhibit anti-tumor immunity [356]. Nitration of CCL2 cannot induce T cell migration but does not affect the migration of MDSCs, thereby exacerbating the TIME to some extent.

In the context of oxidation, polyunsaturated fatty acids (PUFAs) play a crucial role in free radical-mediated peroxidation. The accumulation of oxidized lipids, such as prostaglandin E2 (PGE2), fatty acid transport protein 2 (FATP2), and arachidonic acid, also contributes to MDSC-mediated immune suppression through oxidative stress [460]. PGE2 can engage in NF-κB signaling to mediate immune suppression; it can activate the Ras/Erk pathway, elevate TGF-β levels, and mediate NK cell inhibition [461]. Recent studies have indicated that lipid peroxidation combined with ferroptosis plays a specific role in the immunosuppression mediated by PMN-MDSCs. Ferroptosis induces the production of lipid peroxidation products in PMN-MDSCs, inhibiting the normal function of T cells [462, 463]. In GBM, MDSCs can take up and utilize lactate produced by tumor cells. Estrogen is also crucial in the immunosuppressive mechanism of MDSCs in GBM [464]. The forkhead box protein P3 (FOXP3) promoter region contains estrogen receptors, and estrogen can inhibit its expression, thereby suppressing the function of Tregs. Progesterone can enhance this process, while androgens can increase FOXP3 expression, inhibiting the immunosuppressive function of MDSCs [464].

Other critical mechanisms include the upregulation of ARG1 via Th2-mediated signaling to deplete arginine [465], the upregulation of solute carrier family 7 members 11 (SLC7A11) to limit cysteine utilization [466], the increased activity of IDO to decrease local tryptophan levels [467], and the increased activity of IDO to decrease local tryptophan levels [466, 468]. PMN-MDSCs can also suppress the antigen-presenting capacity of DCs by upregulating myeloperoxidase (MPO) expression. Significant improvements in the cross-presentation of TAAs by DCs were observed in tumor-bearing mice lacking MDSCs or MPO [469, 470]. Furthermore, MPO can catalyze the generation of peroxidized lipids via PMN-MDSCs, contributing to immune suppression [469]. In addition, PI3K-γ has been shown to contribute to the upregulation of iNOS and ARG1 in MDSCs to mediate immunosuppression [471]. PMN-MDSCs can also facilitate tumor angiogenesis by releasing proangiogenic cytokines like basic fibroblast growth factor (bFGF) and VEGF, facilitate metastasis of tumor by releasing matrix metalloproteinases, and contribute to the progression of epithelial-to-mesenchymal transition (EMT) [472].

MDSCs can produce immunosuppressive factors like IL-10 and TGF-β, inducing Treg activation and affecting NK cell function [473]. PMN-MDSCs can directly inhibit NK cell activity by upregulating PD-L1. Most studies suggest that MDSC-mediated immunosuppression of T cell function in lymphoid organs or PB via the ROS pathway requires closer intercellular contact, as the ROS pathway is sensitive, and only closer intercellular contact allows ROS to act quickly for maximum efficiency [474]. However, not all of the above mechanisms operate synchronously, and the specific mechanism depends on the subtype of MDSCs produced in various cancers. The proportion of PMN-MDSCs to M-MDSCs is also crucial for immune suppression, as they have different immunosuppressive mechanisms. PMN-MDSCs are more inclined to induce immunosuppression through PGE2, ROS, ARG1, and PNT, while M-MDSCs rely more on IL-10, TGF-β, PD-L1, and NO [353, 475]. It is noteworthy that male mice have more M-MDSCs, while female mice have more PMN-MDSCs in PB [430]. Therefore, the ROS pathway in PMN-MDSCs requires closer intercellular contact, while M-MDSCs rely on producing large quantities of NO, ARG1, and other immunosuppressive cytokines for immune suppression. The half-life of these molecules is much longer than that of ROS, so M-MDSCs do not need closer attachment with T cells. Therefore, M-MDSCs can effectively inhibit nonspecific responses of T cells, and their suppressive activity is greater than that of PMN-MDSCs on a per-cell basis [476–478]. However, compared to peripheral MDSCs, intratumoral MDSCs exhibit stronger suppressive activity [479, 480]. Different TMEs can explain the distinct ratio of PMN-MDSCs to M-MDSCs or changes in MDSCs function in various tissues.

There is a higher infiltration of PMN-MDSCs in IDH-mutant GBM compared to IDH-WT. However, while M-MDSCs infiltrate less, their immunosuppressive effect is more pronounced in GBM. In addition to the previously mentioned inhibitory mechanisms, hypoxia-inducible heterogeneous nuclear ribonucleoprotein A1 (hnRNPA1) promotes exosome packaging miRNA [341, 481]. MDSCs can take up exosomes, activating MDSCs through dual-specificity phosphatase-3 (DUSP3)/ERK signal and inhibiting T cells through PD-L1 through a HIF-1α-dependent pathway [353]. Current research suggests that the NF-κB-related pathway is crucial in mediating TIME development in GBM and determining the anti-inflammatory or proinflammatory phenotype of MDSCs [482]. The NF-κB pathway, along with the JAK pathway, is associated with the anti-inflammatory pathways linked to MDSCs [482]. It can increase IDO levels through the STAT3 pathway, thereby enhancing the significant immunosuppressive function of MDSCs [482, 483]. The use of NF-κB inhibitors in combination with standard GBM treatment regimens, such as TMZ, can enhance anti-tumor immunity in GBM mouse models [482].

### Heterogeneity of MDSC regulated by the TME

#### Expression profile of MDSC in different tumors

MDSCs exhibit distinct gene expression profiles and characteristics depending on their infiltration into different organs. Recent studies have analyzed individual subtypes of MDSCs, and the results indicate that the TME may enhance the function of MDSCs by altering their properties. PMN-MDSCs exhibit higher generation of inflammatory cytokines and activation of downstream targets in the NF-κB signaling pathway [353, 484], including IL-6, M-CSF, IFN-γ, ERS regulatory factors, and mitogen-activated protein kinase (MAPK) signal [353]. While M-MDSCs upregulate other factors, like IL-6, TGF-β, and PI3K [480]. MDSCs within prostate or lung cancer have higher expression levels of ARG1, ARG2, NOS2, NOS3, and S100A9 than splenic MDSCs, with ARG1 being the highest. This effect is associated with the significantly enhanced inhibitory activity of MDSCs in the TME [485–488]. As for myeloma, NF-κB pathway-related genes, IRF1, COX2/PTGS2, CSF-1, IL-4R, STAT1, STAT3, STAT6, and IL-8 is high expression, promoting MDSC maturation and infiltration, thereby enhancing the TIME [489]. HIF-1α plays a crucial component in differentiating M-MDSCs into TAMs [479]. It facilitates the immune inhibitory activity of MDSCs by upregulating iNOS and ARG1 and acting in conjunction with PD-L1 [479]. HIF-1α also regulates glycolysis in MDSCs [490]. Under hypoxic conditions, the tyrosine phosphatase activity of CD45 increases in M-MDSCs, selectively reducing the activity of STAT3 and promoting the transformation of MDSCs into TAMs [491]. The upregulation of sialylation of CD45 protein dimers induces increased expression of the CD45 phosphatase. Thus, treatment with sialidase can eliminate the impact of hypoxia on the excitation and differentiation of STAT3 in MDSCs.

#### Immunosuppressive function of TME-driven MDSC

The TME serves as a critical component in the activation and immunosuppressive function of MDSCs, and emerging evidence suggests that hypoxic conditions within the tumor, particularly through the HIF-1α-associated pathway, play a significant component in this process. As mentioned earlier, MDSCs can hinder the priming of nonspecific antigen-T cells in hypoxic environments [479], thereby reshaping the TME. HIF-1α promotes TAMs differentiating from some MDSCs, inhibiting anti-tumor immunity by downregulating STAT3. In a lung metastasis model, MDSCs differentiate into fibroblasts with the participation of Kruppel-like factor 4 (KLF4) and ferroptosis suppressor protein 1 (Fsp1), contributing to the establishment of the lung metastatic TIME [492]. Moreover, the process of MDSC differentiation into TAMs may involve the regulation of ARG1 and iNOS. MDSCs lacking HIF-1α cannot differentiate into TAMs but acquire the characteristics of DCs [493]. HIF-1α binds to the proximal promoter of PD-1/PD-L1, increasing PD-L1 expression in MDSC membranes and leading to more potent immunosuppressive activity, especially in M-MDSCs [481, 494–496]. In addition, M-MDSCs can be regulated by various factors to differentiate into macrophages. In a breast tumor model, TLR7/8 agonists induce splenic MDSCs to differentiate into macrophages [497]. In an ovarian tumor model, thrombin stimulation can cause peritoneal MONs to differentiate into TAMs [498]. High expression of IL-6 and LIF in ovarian cancer ascites promotes the differentiation of MONs into TAMs [499]. Furthermore, in the spleen, M-MDSCs can differentiate into DCs upon STAT3 inhibition. In vitro, MDSCs can differentiate into Tregs under the induction of IL-10 and IFN-γ [343]. However, the transformation between MDSCs and TAMs has not been observed in GBM.

Variations in glycolysis and oxidative phosphorylation in tumors significantly influence MDSCs’ function in immunosuppression. To sustain the pathologically rapid proliferation in cancer cells, most cancer cells predominantly utilize aerobic glycolysis, which is known as the Warburg effect. In mice, the augmentation of glycolysis is concomitant with the increased activity of ARG1 in MDSCs. The resultant activation of AMP-activated protein kinase (AMPK) enhances ATP synthesis, maintaining the energy supply for MDSCs [500]. Simultaneously, tumor-associated MDSCs elevate FA uptake and engage in FAO, a metabolic shift controlled by lactate and hypoxia. However, the specific regulatory mechanisms of this process and its potential implications for targeted therapy remain to be precisely elucidated. MDSCs’ heightened activity in tumor immunosuppression is closely related to the increased FAO-related gene expression. This effect can be mitigated by FAO inhibitors [480]. Spleen-derived MDSCs restrain immune reactions by antigens in T cells through the ROS-dependent pathway. Similarly, tumor-derived MDSCs exhibit more potent antigen-specific suppression activity, primarily suppressing responses to anti-CD3/28 stimulation through the production of NO and secretion of ARG1 [479].

Certainly, GBM exhibits dynamic changes, and it is imperative to scrutinize the interactions among immune components from the perspective of spatiotemporal dynamic evolution. In recent years, the fusion of scRNA-seq with lineage tracing has facilitated researchers in gaining insights into the dynamic evolution within the GBM TME. As previously discussed, the early phases of GBM development are characterized by pro-inflammatory microglia and innate immunity [395]. However, these microglia are swiftly modified by tumor cells to foster tumor growth [501]. Simultaneously, bone marrow-derived MONs are recruited in the initial stages, expediting disease progression [502]. Conversely, the later stages of GBM predominantly consist of anti-inflammatory macrophages and MDSCs [270]. Recent studies, however, reveal that this macrophage population is more akin to microglia in terms of single-cell typing [395]. e-MDSC components in GBM, known as e-MDSC, may evolve into M-MDSC during GBM development, engaging in interactions with GSCs to sustain GSC growth and facilitate GBM infiltration into the pseudopalisading region [383]. Lineage tracing results further indicate that the early stages of GBM prompt the urgent mobilization of bone marrow to generate MDSCs [395]. Neutrophils are observed to infiltrate the early stages of mesenchymal subtypes GBM [320], initially exerting a tumor-suppressive role through their cytotoxic and immuno-activating activities [503]. However, they transition to a pro-tumor phenotype during tumor development, expediting tumor growth. Similar to PMN-MDSCs, neutrophils are present only in the early and late stages of GBM, a process potentially associated with BBB disruption [395]. Regarding T cells, they exhibit a "rejection" effect in GBM TME, resulting in minimal internal effector T cell infiltration [38]. Only in the early stages of GBM development do CD8^+^ T cells exhibit normal function; however, due to insufficient stimulation, they enter a non-responsive state. T cells are likely to elicit a response, but subsequently, GBM antigens inhibit T cell activity [504]. Consequently, most T cells comprise immunosuppressive Tregs, persistently circulating throughout GBM development [505]. For B cells, their recruitment to the GBM microenvironment occurs early on, exerting inhibitory effects. Furthermore, MDSCs undergo conversion to Bregs mediated by PD-L1, intensifying inhibitory effects [452].

## Current treatment strategies and progress of glioblastoma

**Fig. 6:**
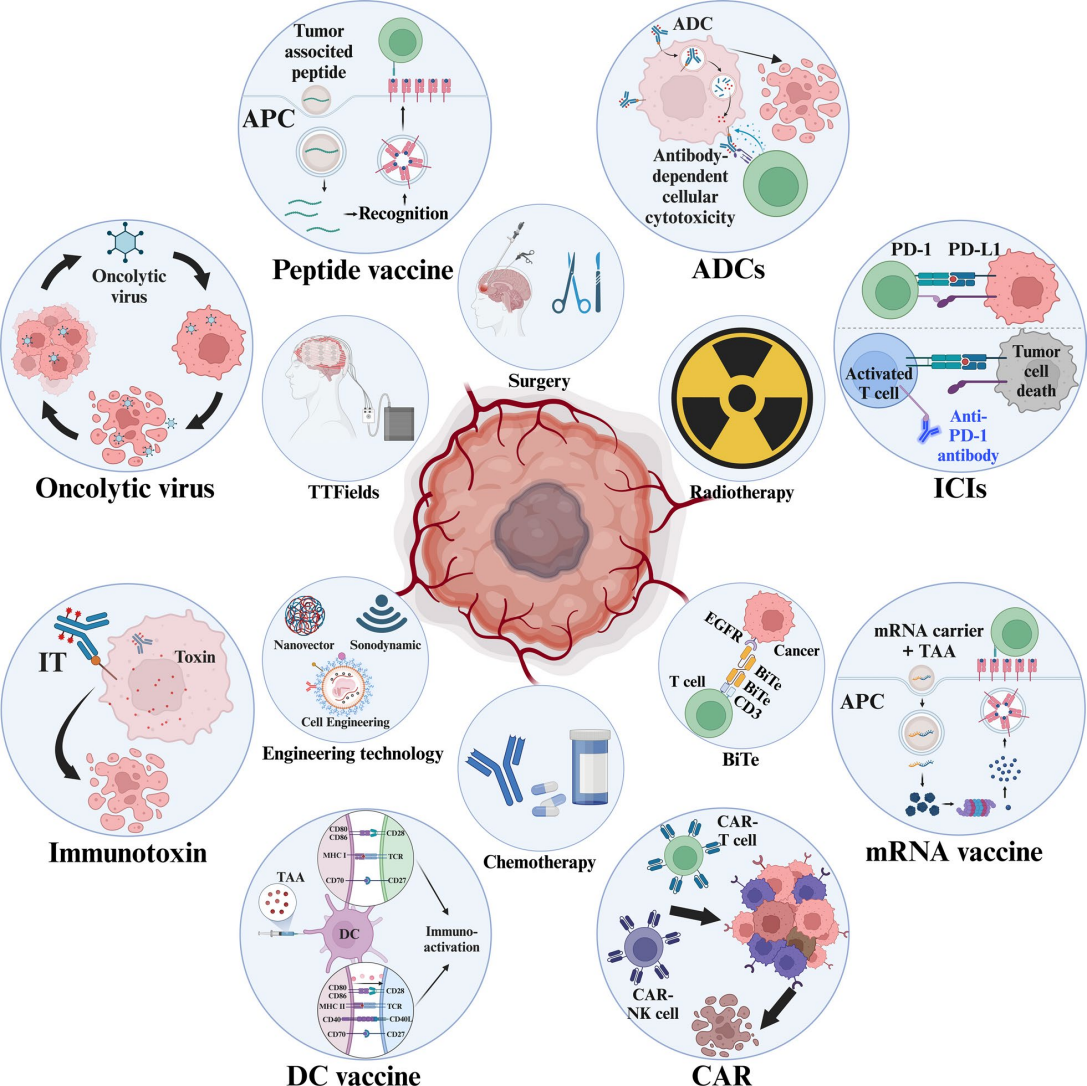
Existing therapeutic strategies against GBM. Currently, there are various therapeutic strategies for GBM, but single-targeted therapy has poor efficacy, and combining multiple treatments is necessary to achieve therapeutic efficacy. The current view is that the initial treatment consists of surgery, RT, and chemotherapy, followed by a variety of other targeted therapies, including immunotherapy, tumor-related vaccine therapy, virus-killing therapy, engineering-based adjuvant therapy, and TTFields. *CAR* chimeric antigen receptor; *BiTe* bispecific T-cell engager; *DC* dendritic cell; *ADC* antibody–drug conjugate; *TTField* tumor treatment field

The conventional treatment paradigm for GBM involves gross total resection (GTR) whenever feasible, followed by adjuvant RT and chemotherapy, typically utilizing TMZ [1]. The STUPP therapy (postoperative RT combined with TMZ) proposed by Stupp et al. in 2005 was previously considered the gold standard for GBM treatment, and it is still a kind of chief treatment in most GBM cases today [144]. This approach, established in an era with limited genetic mutation testing, has demonstrated effectiveness. Despite the emergence of alternative treatments, it remains the primary therapeutic strategy for GBM in cases where specific target sites are not well defined. According to diagnosed tumor position and magnitude, patients commonly receive tumor resection first, following the combination of chemotherapy and RT, incorporating emerging therapies as deemed appropriate. Clinical studies have consistently indicated that aggressive surgical tumor resection correlates with favorable outcomes for GBM patients [327]. However, owing to the diverse locations of brain tumor growth, the surgical approach and prognosis can vary. GTR may not be achievable for all GBMs, especially those in functional areas or proximity to the brainstem, where subtotal resection (STR) might be the chosen course. Given the recurrence tendencies of GBM and the limitations of surgical resection, reliance solely on conventional RT and chemotherapy often proves inadequate. Consequently, various innovative treatment approaches have recently been developed for GBM. Figure 6 provides an overview of the existing treatment strategies for GBM.

### ICI therapy in glioblastoma

ICIs represent an extensively researched class of immunotherapy drugs for GBM, demonstrating efficacy in clinical trials across various malignancies [28]. Prominent targets in ICI therapy, like PD-L1 and CTLA-4, have exhibited promising outcomes in numerous tumors [506]. Data from multiple omics studies and clinical samples underscore elevated PD-L1 expression in GBM, positioning it as a potential and promising immunotherapeutic target [507]. Preclinical findings suggest that anti-PD-1/PD-L1 therapy can shift macrophage polarization from M2 to M1, transforming the immunosuppressive microenvironment into a pro-inflammatory state and ultimately prolonging survival in GBM-afflicted mice [508]. In the GBM mouse model, CTLA-4 blockage can recover CD4^+^ T-cell proliferation, producing stronger anti-tumor ability, while T cells are conferred resistance to Treg suppression in tumors to significantly prolong the survival of mice without causing experimental allergic encephalomyelitis (EAE) [509]. LAG3, also known as CD223, is a marker of exhaustion in T cells expressed on various T-cell surfaces and significantly reduces their ability to produce IFN-γ [510], which is expressed in tumor-associated perivascular lymphocytes and tumor-infiltrating lymphocytes (TILs) in human GBM [511]. Preclinical models have shown that early blocking of LAG3 significantly promotes prognosis in mice with GBM and is highly effective in eradicating tumors along with anti-PD-1/PD-L1 therapy. T-cell immune receptor with Ig and ITIM domains (TIGIT) is another nonclassical checkpoint expressed in various immune cells, like activated T cells, Tregs, and NK cells [512]. Its high expression has been shown to have an overall inhibitory phenotype in various tumor models, which is associated with reduced production of tumor-killing related cytokines and poor survival. Combined anti-TIGIT with anti-PD-1/PD-L1 significantly improved the survival in GBM mouse models compared with only anti-PD-1/PD-L1 therapy, which was attributed to enhancing the function of T cell and downregulating PMN-MDSCs and DCs amount [235, 513]. TIM-3, a membrane protein, is selectively expressed in immune cells, which acts as an immune checkpoint to regulate innate and adaptive immunity [514]. Studies have shown that it is one of the most up-regulated co-inhibitory immune checkpoints and is closely related to the poor prognosis of GBM [31]. Blocking TIM-3 not only inhibited its induction of macrophage migration and transition to a pro-tumor phenotype but also inhibited the tumorigenicity of GBM in vivo, thereby extending mouse survival. Furthermore, TIM3’s expression upregulates in cancer cells, microglias, and macrophages within TME in diffuse intrinsic pontine glioma (DIPG) patients. Blocking TIM-3 can directly inhibit tumor growth and strengthen CD8^+^ T-cell and microglia's function, resulting in durable anti-tumor immune memory, thereby eliminating tumors and preventing their recurrence [32]. Despite these encouraging preclinical results, clinical trials involving PD-1, CTLA-4, and other immunotherapies for GBM have, regrettably, not yielded substantial success. Even though combinations of ICIs with various adjuvant therapies have shown promise in preclinical models, translating these results into successful clinical outcomes remains a significant challenge [515–517]. Ongoing clinical trials investigating immunotherapy in GBM are outlined in Table 4.

**Table 4:** Current ongoing clinical trials based on immunotherapies for glioblastoma

Agents	Targets	Phase	Status	Mechanism	Trial ID
Nivolumab + Bevacizumab	PD-1 and VEGFA in rGBM	III	Active	Inhibit the GBM growth and Terminate the immunosuppression microenvironment	NCT02017717
Pembrolizumab	PD-1 in rGBM	II	Active	Terminate the immunosuppression microenvironment, especially macrophage	NCT02337686
Nivolumab + RT/TMZ	PD-1 and me-MGMT	III	Active	Terminate the immunosuppression microenvironment	NCT02667587
BLZ945 + PDR001	PD-1-CSF-1R	I/II	Terminated	Terminate the immunosuppression microenvironment	NCT02829723
Durvalumab + RT	PD-1	I/II	Active	Terminate the immunosuppression microenvironment	NCT02866747
Atezolizumab + RT/TMZ	PD-1	I/II	Active	Terminate the immunosuppression microenvironment	NCT03174197
Pembrolizumab + RT/TMZ	PD-1	II	Suspended	Terminate the immunosuppression microenvironment	NCT03197506
Ipilimumab and Nivolumab	CTLA-4 and PD-1	I	Active	Terminate the immunosuppression microenvironment	NCT03233152
Retifanlimab + Bevacizumab/Epacadostat + RT	PD-1, IDO and VEGFA	II	Active	Terminate the immunosuppression microenvironment	NCT03532295
Ipilimumab and Atezolizumab	CTLA-4 and PD-1	I/II	Recruiting	Terminate the immunosuppression microenvironment	NCT03673787
MK-4166 + Nivolumab + IDO1 inhibitor INCB024360 + Ipilimumab	CTLA-4,PD-1, GITR and IDO1	I	Terminated	Terminate the immunosuppression microenvironment	NCT03707457
Nivolumab	PD-1 in IDH mutation	II	Recruiting	Terminate the immunosuppression microenvironment	NCT03718767
Bevacizumab + Nivolumab + RT	PD-1 and VEGFA in rGBM	II	Active	Inhibit the GBM growth and Terminate the immunosuppression microenvironment	NCT03743662
Pembrolizumab + Lenvatinib	PD-1 and VEGFR	II	Active	Inhibit the GBM growth and Terminate the immunosuppression microenvironment	NCT03797326
Pembrolizumab + RT/TMZ	PD-1	II	Active	Terminate the immunosuppression microenvironment	NCT03899857
MBG453 and Spartalizumab + RT	PD-1 and TIM-3	I	Active	Terminate the immunosuppression microenvironment, especially T cell	NCT03961971
BMS-986205 and Nivolumab + RT/TMZ	PD-1 and IDO1	I	Active	Terminate the immunosuppression microenvironment, especially macrophage	NCT04047706
Indoximod + RT/TMZ	IDO	II	Recruiting	Terminate the immunosuppression microenvironment, especially macrophage	NCT04049669
Indoximod + Ibrutinib	IDO and BTK	I	Recruiting	Terminate the immunosuppression microenvironment, especially macrophage	NCT04049669
Ipilimumab and Nivolumab	CTLA-4 and PD-1 in IDH mutation	II	Recruiting	Terminate the immunosuppression microenvironment	NCT04145115
INCMGA00012 and INCAGN01876 + RT/Surgery	GITR and PD-1	II	Active	Terminate the immunosuppression microenvironment	NCT04225039
Ipilimumab and Nivolumab	CTLA-4 and PD-1 in Children	I	Active	Terminate the immunosuppression microenvironment	NCT04323046
Ipilimumab and Nivolumab + RT/TMZ	CTLA-4 and PD-1 in nonme-MGMT	II/III	Active	Terminate the immunosuppression microenvironment	NCT04396860
Camrelizumab + RT/TMZ	PD-1	II	Recruiting	Terminate the immunosuppression microenvironment	NCT04583020
Nivolumab + Ipilimumab	CTLA-4 and PD-1	I	Recruiting	Terminate the immunosuppression microenvironment	NCT04606316
AB154 + AB122	PD-1 and TIGIT	I	Recruiting	Terminate the immunosuppression microenvironment, especially T cell	NCT04656535
Regorafenib + Nivolumab	PD-1 and VEGFR	II	Active	Inhibit the GBM growth and Terminate the immunosuppression microenvironment	NCT04704154
Atezolizumab + Tocilizumab + RT	IL6 and PD-1	II	Suspended	Terminate the immunosuppression microenvironment, especially pro-inflammation	NCT04729959
Ipilimumab and Nivolumab + TMZ	CTLA-4 and PD-1	II	Recruiting	Terminate the immunosuppression microenvironment	NCT04817254
ASP8374 + Cemiplimab	TIGIT and PD-1 in rGBM	I	Active	Terminate the immunosuppression microenvironment, especially CD8 + T cell	NCT04826393
Daratumumab + RT/TMZ	CD38	I/II	Recruiting	Terminate the immunosuppression microenvironment, especially B cell	NCT04922723
Camrelizumab and Bevacizumab	PD-1 and VEGFA in rGBM	II	Suspended	Terminate the immunosuppression microenvironment	NCT04952571
Pembrolizumab + RT	PD-1	I/II	Recruiting	Terminate the immunosuppression microenvironment, especially CD8 + T cell	NCT04977375

*GBM* Glioblastoma; *TIME* Tumor immune environment; *rGBM* Recurrent glioblastoma; *RT* Radiotherapy; *TMZ* Temozolomide; *MGMT* Methyl guanine methyl transferase; *me-MGMT* methylated MGMT; *nonme-MGMT* non-methylated MGMT

Presently, the latest preclinical trials involve combining ICIs with other treatment strategies to achieve effective progress in terms of survival benefits. Notably, the simultaneous blockade of PD-1, VEGF, and angiopoietin 2 (Ang-2/ANGPT2) has shown significant promise in prolonging the survival time of GBM mice. This triple therapy demonstrated improvements in the number of CTLs and reduced the infiltration of MDSCs and Tregs. Transcriptome analysis of the GBM microvasculature indicated that triple therapy could promote tumor vascular normalization, potentially limiting or preventing cancer progression and metastasis [515]. Despite these promising preclinical results, translating such findings into successful clinical outcomes has proven challenging. Clinical trials involving ICIs in combination therapy have been disappointing, partly due to the BBB, which hinders these agents from reaching effective therapeutic concentrations within the intracranial space [518]. Consequently, researchers are exploring small molecule immunotargeted drugs, particularly immunomodulatory cytokines, as a research hotspot in immunomodulatory therapy. Immunomodulatory cytokines like TNF-α and IFN-α can traverse the BBB and have been demonstrated effective at reversing GBM-induced immunosuppression. Therapeutic regimens employing IFN-α and TNF-α to counteract the immunosuppressive microenvironment of GBM have shown promise in preclinical models and early clinical trials [519–521]. IFN-α can facilitate the differentiation of DCs, strengthen NK-cell, T-cell, and macrophage’s anti-tumor ability, as well as increase TAA expression. Additionally, IFN-α has exhibited the ability to prohibit tumor angiogenesis through disrupting ECs growth and promoting the synthesis of angiosuppressive chemokines such as CXCL1, CXCL9, and CXCL10 [522]. TNF-α has also been demonstrated to induce DCs’ maturation and enhance the infiltration of T cells within GBM mice models [523].

ICI therapy represents a prominent and promising field in cancer treatment and has demonstrated benefits in various tumors. However, there are no ICIs for GBM that get permission from the Food and Drug Administration (FDA), although a few are in clinical trials. ICIs remain ineffective against GBM as monotherapy, indicating there are limitations and deficiencies in our current preclinical model. Current preclinical models have been established by orthotopic injection of murine glioma cell, patient-derived xenograft model, or genetically engineered mouse model, which cannot recapitulate the complexity and heterogeneity of the patient’s GBM microenvironment [524]. Therefore, mouse models for evaluating immunotherapies in preclinical settings must be carefully considered. In addition, there is a BBB in the brain, which strictly regulates the barrier between the CNS and the PB, allowing small-molecule, lipid-soluble drugs to be passively diffused across, but water-soluble drugs and large-molecule to be largely inaccessible since tightknit connectivity networks [525]. Thus, many drugs, such as monoclonal antibodies, have poor anti-tumor effects due to the insufficient delivery of the BBB. It is necessary to consider improving the delivery system to increase drug delivery to cancer. However, with GBM progression, the integrity of the BBB is gradually lost, followed by the increase of tight junction permeability [526]. Its disruption allows for the delivery of drugs, which can recruit immune cells from the peripheral; however, it strengthens tumorigenicity through facilitating pro-tumorigenic-cell infiltration, like immunosuppressive macrophages [527]. In addition, the BBB is kept perfectly in some areas of the tumor [279]. Thus, systemic treatment of GBM has to conquer these difficult limits to become valid. In addition, ICI can lead to treatment-related toxicity. The expression levels of CTLA-4 and PD-1 strike a subtle balance in self-immunotolerance and autoimmunity [528, 529]. The direct toxicity of anti-CTLA-4 and anti-PD-1/PD-L1 is little, and their vital toxicity is an autoimmune disease known as delayed immune-related side effects (irSEs), which can influence all organs, especially skin, kidney, endocrine system, and gastrointestinal tract [530]. It is well known that the unique heterogeneity of GBM leads to its resistance to most treatments. It has a unique TME consisting of 20% to 40% immune cells, mostly from bone marrow, with various proportions in bone marrow-derived circulating macrophages and tissue-resident microglia [531]. The MON-derived macrophage and lymphocyte infiltration are higher in IDH-WT GBM. However, the immune pool in IDH-mutant GBM is almost microglia [294]. Currently, the standard therapy for adult GBM is RT and TMZ chemotherapy, followed by maintenance TMZ chemotherapy after surgical resection [10]. However, in preclinical models and GBM patients, systemic chemotherapy, including TMZ, has an inherent immunosuppressive effect, which allows the already minimal number of T cells in TME to rapidly deplete or develop tolerance to tumor antigens, leading to a possible failure of immunotherapy to promote TILs effector function [532]. Since there is little T cell infiltration in GBM, neoadjuvant immunotherapy provides drug therapy before chemotherapy, RT, and surgical resection can help address complications associated with its immunosuppressive environment [30]. It has been shown that PD-1 blockade for neoadjuvant therapy leads to the upregulation of T cells and IFN genes within the tumor and the reduction of the cell cycle in rGBM, thereby promoting anti-tumor responses [533]. Therefore, combination therapy and neoadjuvant therapy are necessary to address the unique immune microenvironment of GBM, such as multi-factor immunosuppressive TME and heterogeneity in cancer. Additionally, TMZ can reduce the number of anti-inflammatory MDSCs, although their number significantly increases at the late stage of the tumor, which is the focus of current ICI therapy [294, 395]. In GBM, the origin and function of MDSCs also vary depending on the gender of the patient [430]. Notably, therapies targeting MDSCs will be discussed later, showcasing significant benefits in improving the immunosuppressive microenvironment of GBM.

### Molecular-based therapy in glioblastoma

Targeted therapy in cancer treatment focuses on addressing proteins that regulate the growth, division, and spread of tumor cells while minimizing the impact on normal cells. This approach aligns with the principles of precision medicine, tailoring treatments based on the specific characteristics of the individual and their cancer [534]. As our understanding of the genetic and protein changes underlying tumors deepens, researchers can design treatments targeting these aberrations. The two main targeted therapies are small-molecule drugs and monoclonal antibodies. Monoclonal antibodies, or therapeutic antibodies, are laboratory-produced proteins designed to bind to specific targets on tumor cells. They can mark cancer cells, making them more visible to the immune system for detection and destruction. Some monoclonal antibodies directly inhibit tumor cell growth or trigger self-destructive mechanisms in these cells. Additionally, certain antibodies are engineered to carry toxins that can selectively destroy tumor cells. Small-molecule drugs, compact enough to traverse the BBB, can bind to specific targets on tumor cells, impeding their growth or inducing cell death. This makes them particularly relevant for brain cancers such as GBM. In addition to targeting tumor proto-oncogenes or mutated genes, emerging targeted therapies encompass tumor epigenetics and metabolism. This diversification allows for a more comprehensive and personalized approach to cancer treatment. Table 5 provides an overview of ongoing clinical trials focused on targeted therapies for GBM.

**Table 5:** Current clinical trials based on targeted therapies or small-molecule drugs for glioblastoma

Agents	Targets	Phase	Status	Mechanism	Trial ID
Rapamycin	mTOR	I/II	Completed	Inhibit tumor growth	NCT00047073
Gefitinib + RT	EGFR	I/II	Completed	Inhibit tumor growth	NCT00052208
AP23573	mTOR	I	Completed	Inhibit tumor growth	NCT00087451
Vorinostat + TMZ	Class I, II and III HDAC	I	Active	Inhibit HDAC and suppress GBM cell transcription	NCT00268385
ZD6474 + RT/TMZ	VEGF + EGF	I/II	Completed	Suppress tumor growth	NCT00441142
Erlotinib hydrochloride + Sorafenib tosylate	EGFR + BRAF + CRAF	II	Completed	Inhibit tumor growth	NCT00445588
Pazopanib hydrochloride	VEGFR	II	Completed	Inhibit tumor and abnormal vessel growth	NCT00459381
Sunitinib	PDGFR	II	Unknown	Inhibit tumor growth	NCT00535379
Sorafenib + RT/TMZ	Raf	II	Completed	Inhibit tumor growth	NCT00544817
Vorinostat + Isotretinoin + TMZ	Class I, II and III HDAC	I/II	Active	Inhibit HDAC and suppress GBM cell transcription	NCT00555399
TAVARLIN	Keton	I	Completed	The ketogenic diet was used to limit the energy acquisition of tumor cells	NCT00575146
Sorafenib + Temozolomide	Raf	II	Completed	Inhibit tumor growth	NCT00597493
Cilengitide	αvβ5	II	Completed	Inhibition of TGF-β/Smad signaling pathway and regulation of PD-L1 expression	NCT00679354
Cilengitide + RT/TMZ	ανβ3 and ανβ5	III	Completed	Inhibition of TGF-β/Smad signaling pathway and regulation of PD-L1 expression	NCT00689221
Olaratumab + Ramucirumab	PDGFRA	II	Completed	Inhibit the tumor growth and expansion	NCT00895180
ZD6474 + Carboplatin	VEGFR2/KDR	II	Completed	Inhibit tumor growth	NCT00995007
Perifosine + Temsirolimus	Akt + mTOR	I/II	Completed	Inhibit tumor growth	NCT01051557
CTO + RT/TMZ	VEGF	I	Active	Inhibit tumor and abnormal vessel growth	NCT01107522
Lomustine + TMZ	DNA	III	Completed	Induce DNA damage to inhibit tumor growth	NCT01149109
LY2157299 + RT/TMZ	TGF-βRI	I/II	Completed	Inhibit tumor growth	NCT01220271
Bevacizumab + Vorinostat + TMZ	HDAC + VEGFR	II/III	Active	Inhibit tumor growth	NCT01236560
XL765 + XL147	PI3K + mTOR	I	Completed	Inhibit tumor growth and promote apoptosis	NCT01240460
Bevacizumab	VEGFR	I/II	Recruiting	Inhibit GBM growth and cause certain destruction of the blood–brain barrier	NCT01269853
BKM120	PI3K	II	Completed	Inhibit tumor growth	NCT01339052
PLX3397	CSF-1R in rGBM	II	Terminated	Inhibit tumor growth	NCT01349036
MFGR1877S	FGFR3	I	Completed	Inhibit tumor growth	NCT01363024
Mefloquine + Memantine + TMZ	KvQT1 + NMDAR	I	Active	Inhibit tumor growth	NCT01430351
Sorafenib + Everolimus	Raf + mTOR	I/II	Completed	Inhibit tumor growth	NCT01434602
GSK2636771	PI3K	I	Completed	Inhibit tumor growth	NCT01458067
BKM120 + RT/TMZ	PI3K	I	Completed	Inhibit tumor growth	NCT01473901
PF-299804	ERBB	II	Completed	Inhibit tumor growth	NCT01520870
ERKD	Keton	None	Active	The ketogenic diet was used to limit the energy acquisition of tumor cells	NCT01535911
GDC-0084	PI3K	I	Completed	Inhibit tumor growth	NCT01547546
LY2157299 + Lomustine	TGF-βRI + DNA	II	Active	Inhibit GBM growth and cause certain destruction of the blood–brain barrier	NCT01582269
Lapatinib + RT/TMZ	ErbB-2 + EGFR	II	Active	Inhibit tumor growth	NCT01591577
AXL1717	IGF-1R	I/II	Terminated	Inhibit tumor growth	NCT01721577
Calorie-restricted ketogenic diet and transient fasting	Keton	None	Completed	The ketogenic diet was used to limit the energy acquisition of tumor cells	NCT01754350
PLX3397 + RT/TMZ	IGF-1R	I/II	Completed	Inhibit tumor growth	NCT01790503
SIACI of Erbitux and Bevacizumab	EGFR + VEGFR	I/II	Terminated	Inhibit tumor and abnormal vessel growth	NCT01884740
WP1066	STAT3	I	Completed	Inhibit tumor growth	NCT01904123
Topotecan + Pazopanib	Topoisomerase I + VEGFR	II	Completed	Inhibit tumor and abnormal vessel growth	NCT01931098
Plerixafor + RT/TMZ	CXCR4	I/II	Completed	Promote lymphocyte recruitment	NCT01977677
Ketogenic Diet	Keton	I/II	Terminated	The ketogenic diet was used to limit the energy acquisition of tumor cells	NCT02046187
Belinostat + RT	Class I, II and IV HDAC	II	Active	Inhibit HDAC and suppress GBM cell transcription	NCT02137759
Metformin + Low carbohydrate diet	Glucose	I	Unknown	The diet was used to limit the energy acquisition of tumor cells	NCT02149459
CC-486 + Vidaza	DNA/RNA methyltransferases	I	Completed	Damage to DNA inhibit tumor cell growth	NCT02223052
Palbociclib isethionate	CDK4 and CDK6	I	Terminated	Induce tumor cell cycle arrest and inhibit growth	NCT02255461
Enasidenib	IDH2	I/II	Completed	Inhibit tumor growth	NCT02273739
MK-8628	BRD2, 3 and 4 in rGBM	II	Terminated	Inhibit BRD and suppress GBM cells transcription	NCT02296476
Ribociclib	CDK4, CDK6, Rb and E2F	I	Unknown	Induce tumor cell cycle arrest and inhibit growth	NCT02345824
Varlilumab + Nivolumab	CD27 + PD-1	I/II	Completed	Inhibit tumor growth and modulate anti-tumor immune	NCT02335918
INC280 + Bevacizumab	c-MET and VEGFR	I	Completed	Inhibit c-MET-dependent tumor growth and tumor migration	NCT02386826
Galunisertib + Nivolumab	TGF-βRI + PD-1	I/II	Completed	Inhibit tumor growth and modulate anti-tumor immune	NCT02423343
MK 3475	PI3K/Akt	I/II	Unknown	Inhibit tumor growth	NCT02430363
AZD2014	mTOR	I	Completed	Inhibit tumor growth	NCT02619864
BMS 986016 + Anti-CD137/Anti-PD-1	LAG-3, CD137 and PD-1	I	Completed	Restore anti-tumor immunity, especially T cell	NCT02658981
Disulfiram + Chemotherapy	ALDH1	II/III	Completed	Inhibit replication of tumor cell	NCT02678975
Vorinostat + TMZ	Class I, II and III HDAC	I	Active	Inhibit expansion of tumor cell; Inhibit HDAC and suppress GBM cell transcription	NCT00268385
BMS-986179 + Nivolumab + rHuPH20	CD73, PD-1 and PH20	I/II	Completed	Restore anti-tumor immunity, especially T cell	NCT02754141
Metformin + RT/TMZ	AMPK	II	Active	Inhibit tumor growth	NCT02780024
Intra-arterial Cetuximab + Mannitol + RT	EGFR	II	Recruiting	Inhibit tumor growth	NCT02800486
BLZ945 + PDR001	CSF-1R + PD-1	I/II	Terminated	Inhibit tumor growth and recruitemrnt of lymphcyte	NCT02829723
Intra-arterial Cetuximab + Mannitol	EGFR	I/II	Recruiting	Inhibit tumor growth	NCT02861898
hrBMP4 + CED	hrBMP4	I	Unknown	Inhibition of tumor growth and mesenchymal transformation	NCT02869243
Regorafenib + Lomustine	VEGFR + DNA	II	Completed	Inhibit tumor and abnormal vessel growth	NCT02926222
Sunitinib + RT/TMZ	VEGFR/PDGFR	II	Unknown	Inhibit tumor and promote vascular normalization	NCT02928575
Bevacizumab and Cediranib/Olaparib	VEGFA in rGBM	II	Active	Promote vascular normalization in GBM	NCT02974621
Abemaciclib	CDK4 and CDK6	II	Active	Induce tumor cell cycle arrest	NCT02981940
Disulfiram/Copper + TMZ	ALDH1	II	Completed	Inhibit expansion of tumor cell	NCT03034135
Navtemadlin + RT	MDM2	I	Recruiting	Restore P53 activity to kill tumor	NCT03107780
Disulfiram + Metformin	ALDH1 + AMPK	I	Terminated	Inhibit tumor growth	NCT03151772
TG02 + RT/TMZ	CDK/JAK2/FLT3	I	Completed	Inhibit tumor growth	NCT03224104
Valproic Acid + TMZ	HDAC	III	Recruiting	Inhibit HDAC and suppress GBM cell transcription	NCT03243461
Metformin + TMZ	AMPK	II	Completed	Inhibit tumor growth	NCT03243851
Axitinib + Avelumab	VEGFR/PDGFR + PD-L1	II	Completed	Inhibit tumor and promote vascular normalization and restore anti-tumor immunity	NCT03291314
Ketogenic Diet + TMZ	Keton	I	Active	The ketogenic diet was used to limit the energy acquisition of tumor cells	NCT03451799
Paxalisib	PI3K/mTOR	II	Active	Inhibit tumor growth	NCT03522298
Dabrafenib + Trametinib	Raf in BRAF V600E	None	Terminated	Inhibit tumor growth	NCT03593993
Panobinostat + Everolimus	HDAC + mTOR	II	Withdraw	Inhibit tumor growth	NCT03632317
Bortezomib + TMZ	MGMT	I/II	Unknown	Promote autophagy of tumor cell	NCT03643549
FT-2102 + Azacitidine/Nivolumab/Gemcitabine/Cisplatin	IDH1, PD-1, DAN methyltransferase and DNA Synthesis	I/II	Completed	Restore anti-tumor immunity	NCT03684811
Temferon	IFN-α	I/II	Recruiting	Activate anti-tumor immunity	NCT03866109
Fimepinostat	PI3K and HDAC classes I, II	I	Active	Inhibit HDAC and suppress GBM cell transcription	NCT03893487
BGB-290 + RT/TMZ	PARP	I/II	Suspended	Inhibit tumor growth	NCT03914742
Dabrafenib Mesylate + Trametinib Dimethyl Sulfoxide	Raf + MEK	II	Recruiting	Inhibit tumor growth	NCT03919071
BGB 324	AXL	I	Suspended	Inhibit tumor growth	NCT03965494
Encorafenib + Binimetinib	BRAF + MEK	II	Active	Inhibit tumor growth	NCT03973918
Dabrafenib + Trametinib	Raf + MEK	IV	Recruiting	Inhibit tumor growth	NCT03975829
Anlotinib	VEGFR/PDGFR/FGFR/c-Kit in rGBM	I/II	Unknown	Inhibit tumor growth	NCT04004975
Regorafenib	VEGFR/PDGFR in rGBM	II	Active	Inhibit tumor and promote vascular normalization	NCT04051606
TPX-0005	ALK	I/II	Recruiting	Inhibit tumor growth	NCT04094610
CC-90010 + RT/TMZ	BET	I	Active	Inhibit tumor growth	NCT04324840
Infigratinib	FGFR in rGBM	I	Terminated	Inhibit tumor growth	NCT04424966
BCA101 + Pembrolizumab	EGFR, TGFβ and PD-1	I	Recruiting	Target EGFR	NCT04429542
Anlotinib + TMZ	VEGFR/PDGFR/FGFR/c-Kit	II	Unknown	Inhibit tumor growth	NCT04547855
OS2966 + CED	ITGB1	I	Terminated	Inhibit the invasion and MET of GBM	NCT04608812
Ketogenic Diet + Metformin	Keton	II	Recruiting	The ketogenic diet was used to limit the energy acquisition of tumor cells	NCT04691960
Regorafenib + Nivolumab	VEGFR/PDGFR + PD-1	II	Active	Restore anti-tumor immunity	NCT04704154
Anlotinib	VEGFR/PDGFR/FGFR/c-Kit in nonme-MGMT GBM	II	Recruiting	Inhibit tumor growth	NCT04725214
Ketogenic Diet + RT/TMZ	Keton	None	Recruiting	The ketogenic diet was used to limit the energy acquisition of tumor cells	NCT04730869
Talazoparib	PARP	II	Recruiting	Inhibit tumor growth	NCT04740190
BPM31510 + Vitamin K1 + RT/TMZ	oxidized CoQ10	II	Recruiting	Activate the apoptosis pathway of mitochondria to kill tumor cells	NCT04752813
Regorafenib 40 MG Oral Tablet	VEGFR	None	Active	Promote vascular normalization	NCT04810182
Metformin + RT/TMZ	AMPK	II	Not yet recruiting	Inhibit tumor growth	NCT04945148
Anlotinib Hydrochloride + RT/TMZ	VEGFR/PDGFR/FGFR/c-Kit	II	Unknown	Inhibit tumor growth	NCT04959500
TAS2940	HER2 + EGFR	I	Recruiting	Inhibit tumor growth	NCT04982926
ONC201 + Paxalisib + RT	Akt/ERK/PI3K/mTOR	II	Recruiting	Inhibit tumor growth	NCT05009992
Anlotinib + AK105 + RT	VEGFR/PDGFR/FGFR/c-Kit/PD-1 in nonme-MGMT GBM	II	Unknown	Restore anti-tumor immunity	NCT05033587
Dichloroacetate	PDK	II	Recruiting	Inhibit tumor glycolysis and promote tumor autophagy	NCT05120284
Paxalisib + Metformin + Ketogenic Diet	Keton/AMPK/PI3K	II	Recruiting	Inhibit tumor growth	NCT05183204
JBI-802	LSD1 + HDAC6	I/II	Recruiting	Inhibit stem cell transformation and immunosuppressive regulation	NCT05268666
MTX110	Class I, II and IV HDAC	I	Recruiting	Inhibit HDAC and suppress GBM cell transcription	NCT05324501
Gabapentin + Sulfasalazine + Memantine + RT/TMZ	GABA + NF-κB + NMDAR	I/II	Recruiting	Inhibit tumor growth	NCT05664464

*RT* Radiotherapy; *TMZ* Temozolomide; *HDAC* Histone deacetylase; *GBM* Glioblastoma; *TMZ* Temozolomide; *BBB* Blood brain barrier; *rGBM* recurrent GBM; *BRD* Bromodomain; *MET* Mesenchymal transformation; *CED* Convection enhanced delivery; *MGMT* Methyl guanine methyl transferase; *nonme-MGMT* nonmethylated MGMT; *PDK* Pyruvate dehydrogenase kinase; *NMDAR* N-methyl-D-aspartic acid receptor

Extensive transcriptomic and proteomic analyses have identified numerous potential therapeutic targets in GBM, with a particular emphasis on angiogenesis as a pivotal process in GBM initiation and progression. Noteworthy interventions targeting VEGF or EGFRvIII, such as bevacizumab and cetuximab, have been extensively investigated in clinical monotherapy, showcasing variable efficacy. VEGF, a key angiogenic factor and regulator of the innate immune response, significantly influences GBM pathology [535–537]. Elevated VEGF levels contribute to a threefold increase in tumor volume, marked vascular architecture remodeling, and an approximately 50% reduction in GAMs infiltration. Bevacizumab, a VEGF inhibitor, promotes tumor vascular normalization, mitigates GBM-related edema, and significantly enhances patient symptoms [538]. EGFRvIII, the predominant mutant form of EGFR in GBM, plays an important component in the progression of tumors. Studies indicate EGFRvIII expressing with a substantial proportion of GBM patients (40% ~ 60%), establishing its significance in regulating angiogenesis, growth, and metastasis [539]. Preclinical studies validate cetuximab's efficacy in suppressing GBM cell growth and enhancing the effectiveness of therapeutic modalities, including radiation therapy [540]. Aquaporin 4 (AQP4), a prominent aquaporin in the CNS, emerges as a crucial determinant of glioma cell fate and an ideal biomarker for precise diagnosis and treatment [246]. TMZ suppresses AQP4 expression through MAPK signaling pathway activation, suggesting the therapeutic potential of targeting the AQP4-MAPK pathway [541]. Inhibition of AQP4 enhances GBM sensitivity to TMZ, influencing TMZ efficacy by regulating sodium pump α3 subunit protein (ATP1A3) [542]. AQP4's role in maintaining BBB integrity positions selective inhibition as a promising avenue for innovative therapies. PDGFRA amplification characterizes proneural subtypes, emphasizing its pivotal role [543–545]. Analysis of the database of TCGA and clinical samples reveals that elevated EPH receptor A2 (EPHA2) expression correlates with PDGF signaling pathway upregulation [151]. Prohibiting EPHA2 and PDGFRA simultaneously shows synergistic results in malignant cells in GBM.

The circadian rhythm, a conserved phenomenon, is a crucial regulatory system maintaining normal cell and tissue homeostasis. It plays a pivotal role in regulating various tumor-related processes, including tumor cell proliferation, survival, metabolism, DNA repair, and inflammation [546]. The transcription factors CLOCK and BMAL1 [308], key components of the circadian rhythm mechanism, form a heterodimeric complex with either pro-tumor or anti-tumor effects depending on the TME and cancer type [69]. In GBM, the CLOCK-BMAL1 complex is identified as an oncogenic factor fostering proliferation and migration in tumor cells [547] through enabing NF-κB signal [548]. Targeting CLOCK or its heterodimeric partner BMAL1 induces cell cycle arrest and apoptosis by attenuating mitochondrial metabolic function and inhibiting key enzymes in the tricarboxylic acid (TCA) cycle [548]. Furthermore, the CLOCK-BMAL1 complex suppresses anti-tumor immunity by upregulating chemokines, leading to immunosuppressive microglial infiltration into the GBM TME [309]. Additionally, it contributes to angiogenesis and cancer progression, associated with adverse clinical outcomes in GBM through activating TANK binding kinase 1 (TBK1) signaling pathway in ECs [549]. Inhibiting the CLOCK-BMAL1 complex counteracts its tumor-promoting effects on GBM and enhances BBB permeability [550, 551], increasing the effective concentration of therapeutic drugs in the brain. This underscores the potential of CLOCK-BMAL1 as an important treatment target in GBM [552, 553].

The CNS is pivotal for development and oncology, exerting regulatory control over stem and precursor cell populations and influencing tumor growth and metastasis. This recognition has given rise to an emerging field known as cancer neuroscience. Increasingly, studies underscore the critical involvement of the nervous system in cancer initiation and metastasis, forming the basis for figuring out the relation of neurological processes and tumorigenesis [554]. For GBM, infiltration into the brain often follows organized anatomical structures, such as blood vessels and white matter tracts containing neuronal axons. This observation suggests the active participation of neuronal populations in GBM progression. Recent investigations into GBM pathobiology reveal a bidirectional signaling relationship between cancers and neurons, establishing a feedback loop characterized by heightened brain activity, increased proliferation, and synaptic integration. This suggests the intriguing possibility that neuronal activity itself contributes to tumor invasion and progression. Specifically, callosal projection neurons in the hemisphere opposite primary GBM drive tumor progression and widespread infiltration, with Ssemaphorin 4F (SEMA4F) emerging as a key regulator dependent on neuronal activity [555]. This finding unveils a novel mechanism in GBM progression regulated by neuronal activity. In the intricate interplay between neurons and GBM, the physical interaction between potassium voltage-gated channel subfamily a regulatory beta subunit 2 (KCNAB2 or Kvβ2) and Ether-a-go-go 2 (EAG2) forms a potassium channel complex, regulating intracellular Ca^2+^ concentration in tumor cells, promoting growth, invasion, and chemoresistance in GBM. Inhibition of the EAG2-Kvβ2 complex mitigates cancer aggressiveness, extending survival time in mice with GBM, even in GBM resistant to TMZ [288, 556, 557]. These findings highlight the potential of targeting the EAG2-Kvβ2 complex as a therapeutic strategy for GBM, particularly in cases where resistance to conventional treatment poses a challenge [558, 559].

Epigenetic modifications, pervasive in tumors, play pivotal roles in establishing and maintaining heterogeneity in GBM. Aberrant epigenetic regulation is a primary driver for GBM initiation, with dysregulation of epigenetic regulators contributing to tumor formation. DNA methylation, orchestrated by DNA methyltransferases (DNMTs), represents a reversible process converting 5-methylcytosine (5mC) to 5-hydroxymethylcytosine (5hmC). Transformations in 5mC to 5hmC patterning are documented in various human cancers, with lower 5hmC levels correlating negatively with glioma grade [560]. Hypermethylation in the promoter of O6-methylguanine DNA methyltransferase (MGMT) occurs in approximately 40% of GBM cases [561], serving as a key marker for evaluating GBM sensitivity to TMZ treatment and prognostic outcomes [562]. Gliomas, including GBM, exhibit overall hypermethylation in CpG islands, which is glioma CpG island methylator phenotype (G-CIMP) [563], which is recognized as a prognostic indicator for glioma patient survival. Most GBMs are characterized as G-CIMP negative [564]. Consequently, drugs designed to suppress DNMTs are anticipated to induce DNA hypomethylation, potentially activating tumor-suppressing genes. DNMT inhibitors, such as 5-azacytidine and decitabine [565], demonstrate anti-tumor effects in preclinical GBM models and FDA approval as Class I epigenetic drugs for treating various tumors [566, 567]. Histone modification, a multifaceted process, involves diverse mechanisms such as lactylation, methylation, ubiquitination, acetylation, phosphorylation, and adenosine diphosphate (ADP) ribosylation [568], facilitated by various enzymes. Aberrations in histone modification contribute significantly to glioma progression, particularly histone acetylation and methylation in GBM [569]. EZH2, known as histone methyltransferase in polycomb repressive complex 2 (PRC2), modulates gene expression [570, 571] by inhibiting PTEN and activating the NF-κB pathway in GBM [572, 573]. Conflicting opinions exist regarding the efficacy of the EZH2 inhibitor tazemetostat in GBM clinical trials, emphasizing the need for cautious consideration until its specific benefits are delineated for GBM patients [574, 575], especially tumors with H3K27M mutation [576–578]. Histone deacetylation, mediated by HDACs, promotes a closed chromatin conformation, inhibiting tumor suppressors [579]. Class I HDACs (HDAC1, 2, 3, and 8) mainly operate within nucleus and primarily inhibit gene transcription, while Class II HDACs (HDAC4, 5, 6, 7, 9, and 10) shuttle between the nucleus and cytoplasm. Class III comprises NAD^+^-dependent protein deacetylases involved in various cellular processes, and Class IV contains HDAC11, whose sequence is homologous to catalytic core regions of Class I and II HDACs. The multifaceted functions of Class III and IV HDACs in GBM pathogenesis have yet to be fully elucidated [580, 581]. Notably, researchers have observed a downregulation in the mRNA levels in Class II and IV HDACs in GBM compared to low-grade astrocytoma [582]. HDAC inhibitors (HDACIs) have become potential treatments in GBM, impacting oncogene transcription, cell cycle regulation, apoptosis, and differentiation [583]. Recent advancements highlight lactate-derived histone lactylation as a novel modification implicated in GBM progression [59]. This modification induced by the Warburg effect upregulates LINC01127 expression via NF-κB signaling pathway, promoting cancer cell self-renewal through the MAP4K4/JNK/NF-κB axis [61]. Inhibiting lactate production, the substrate for histone lactylation, suppresses GBM progression, making lactylation a potential target for GBM treatment. Elevating lactate production within the TME plays a pivotal role in shaping an acidic microenvironment conducive to tumor promotion, supporting tumor growth, and serving as a cellular substrate for lactylation within the microenvironment. Targeting lactylation emerges as a potentially effective treatment strategy for GBM.

Metabolic reprogramming is a prominent hallmark of tumors, with tumor cells autonomously modulating adaptations through diverse metabolic pathways to meet heightened bioenergetic and biosynthetic demands, which are crucial for proliferation and survival while alleviating oxidative stress. In the local microenvironment, poor vascular differentiation leads to inefficient delivery of oxygen, nutrients, and metabolic waste removal, creating conditions where cancer cells, by rapid proliferation, outcompete anti-tumor immune cells for limited nutrients [584]. Consequently, cancer cells establish a unique anti-immune metabolic microenvironment. Immune cells undergo metabolic adaptations associated with a tolerance phenotype, such as T cells relying on aerobic glycolysis and glutamine catabolism [585]. GBM, with heightened metabolic demands, presents an opportunity for treatment by targeting tumor metabolism [569, 586]. The sodium/hydrogen exchanger 1 (NHE1), from SLC9A1, plays a pivotal role in keeping the microenvironment alkaline within the tumor, supporting aerobic glycolysis crucial for tumor progression [587]. High SLC9A1 expression is observed in the classical and mesenchymal subtypes, indicating a positive correlation with GBM malignancy. NHE1 promotes GBM cell migration and invasion, impacting cell adhesion and ECM rearrangement [588]. Inhibition of NHE1 reduces tumor volume, invasion, angiogenesis, TAM infiltration, and cytokine secretion, enhances the immune system to resist tumors, as well as improves sensitivity to anti-PD-1/PD-L1 immunotherapy and TMZ in mice with GBM [589]. GBM's metabolic shift toward glucose oxidation results in elevated ROS production, requiring upregulation of redox pathways, such as glutathione synthesis [590]. BPM31510 and valerenic acid show promise in inducing oxidative stress and inhibiting GBM progression [591, 592]. Lactate, once considered a glycolysis byproduct [593], now plays a crucial role in metabolic coupling, immune responses, and intercellular communication in the TME [594]. Targeting lactate metabolism, specifically monocarboxylate transporters (MCTs) and LDH, presents therapeutic potential [595, 596]. MCT inhibitors and LDHA inhibitors like NHI-1 and NHI-2 show effectiveness against GBM [60, 597, 598], affecting cell viability and inducing apoptosis and differentiation [171]. GBM utilizes an internal immune escape mechanism through LDH5 secretion, suppressing NK cell recognition [196]. Targeting LDH5 may enhance tumor recognition [599]. The IDO pathway [136], perilipin-2 (PLIN2) [600], ketone oxidation [601], and amino acid metabolism [602] are additional GBM treatment targets, often combined with other therapies to enhance efficacy, impacting immune or epigenetic pathways for improved patient survival.

### Targeted CAR modification in glioblastoma

The CAR represents a synthetic modular protein characterized by division into three distinct domains: intracellular, transmembrane, and extracellular domain. The extracellular domain is capable of recognizing target antigens independently of MHC presentation. The transmembrane domain serves the crucial function of integrating the extracellular and intracellular domains, playing a pivotal component in information transmission. The intracellular domain assumes responsibility for T cell stimulation, facilitating proliferation of T cells and inducing cytotoxicity, thereby contributing to the anti-tumor effect [603, 604]. The modification of CAR significantly augments the anti-tumor activity of immune cells [605, 606]. Notably, Table 6 provides a list of CAR immune cells targeting GBM.

**Table 6:** Current CAR and cell therapy-based clinical trials in GBM

Agents	Targets	Phase	Status	Mechanism	Trial ID
Aldesleukin + Autologous lymphocytes	T cell	II	Completed	Enhance anti-tumor immunity	NCT00331526
IL-13 zetakine/Hy/TK CAR-T	IL-13Rot2	I	Completed	CAR-T	NCT00730613
Autologous NK cells	NK cell	I	Suspended	Increase the infiltration of NK cells	NCT00909558
GRm13Z40-2 + CED	CTL	I	Completed	CAR-T	NCT01082926
HER.CAR CMV-specific CTLs	HER2	I	Completed	CAR-T	NCT01109095
E. coli CD-expressing genetically modified NSC	5-FC	I	Completed	Local drug concentration was increased by drug conversion	NCT01172964
Autologous CMV-specific CTL	CMV	I/II	Terminated	Target CMV to kill tumor cells	NCT01205334
EGFRvIII CAR-T	EGFRvIII	I/II	Completed	CAR-T	NCT01454596
ALECSAT	Autologous CTL and NK cell	I	Completed	Enhance anti-tumor immunity	NCT01588769
Allogeneic HCT + Donor NK Cell Infusion	T cell + NK cell	II	Active	Enhance anti-tumor immunity	NCT02100891
hCE1m6-NSC	NSC and Carboxylesterase	I	Active	Enzyme deprivation mediated tumor growth restriction	NCT02192359
IL13Rα2 CAR-T	IL13Rα2	I	Active	CAR-T	NCT02208362
CAR-T-EGFRvIII T cells	EGFRvIII in rGBM	I	Completed	CAR-T	NCT02209376
HER2-specific T cells	HER2	I	Active	CAR-T	NCT02442297
anti-CD133-CAR vector-transduced T cells	CD133	I/II	Completed	CAR-T	NCT02541370
TMZ + Autologous Cytomegalovirus-specific Cytotoxic T-lymphocytes	CMV specific CTL	I/II	Completed	Modulate anti-tumor immunity	NCT02661282
EGFRvIII CAR-T	EGFRvIII	I	Terminated	CAR-T	NCT02664363
EGFRvIII CAR-T	EGFRvIII	I	Unknown	CAR-T	NCT02844062
Anti-PD-L1 CSR T cells	PD-1	I	Unknown	CAR-T	NCT02937844
Antigen-specific IgT cells	T cell in rGBM	I	Recruiting	Modulate anti-tumor immunity	NCT03170141
EGFRvIII CAR-T	EGFRvIII	I	Terminated	CAR-T	NCT03283631
EGFR BiTe + RT/TMZ	EGFR	I	Active	BiTe	NCT03344250
PD1-TIL	PD-1	I	Unknown	Transgenic modified TIL cells target tumor	NCT03347097
NK-92/5.28.z + Ezabenlimab	ErbB2/HER2 + PD-1	I	Recruiting	CAR-NK	NCT03383978
HER2(EQ)BBζ/CD19t + T cells	HER2(EQ)BBζ/CD19t	I	Active	Modulate anti-tumor immunity	NCT03389230
EGFRvIII, IL13Rα2, Her-2, CD133, EphA2, GD2-CAR-T	Multiple antigen	I	Unknown	CAR-NK	NCT03423992
CAR-T-EGFRvIII T cells + Pembrolizumab	EGFRvIII + PD-1	I	Completed	CAR-T	NCT03726515
IL13Rα2 CAR-T + Ipilimumab/Nivolumab	IL13Rα2	I	Recruiting	CAR-T	NCT04003649
CD147-CAR-T	CD147	I	Unknown	CAR-T	NCT04045847
B7-H3 CAR-T + TMZ	B7-H3	I/II	Recruiting	CAR-T	NCT04077866
C7R-GD2.CAR-T	IL-7 + GD-2	I	Recruiting	CAR-T	NCT04099797
DRI cell therapy + TMZ	γδT cell	I	Recruiting	Modulate anti-tumor immunity	NCT04165941
GD2 CAR-T + Chemotherapy	GD2 in H3K27M	I	Recruiting	CAR-T	NCT04196413
Chlorotoxin (EQ)-CD28-CD3zeta-CD19t-CAR-T	MMP2	I	Recruiting	CAR-T	NCT04214392
NK cells	NK cell	I	Not yet recruiting	Increase the infiltration of NK cells	NCT04254419
B7-H3 CAR-T + TMZ	B7-H3	I	Recruiting	CAR-T	NCT04385173
CYNK001-IV and CYNK001-IT	NK cell	I	Terminated	Increase the infiltration of NK cells	NCT04489420
MSC11FCD	MSC and CD	I/II	Completed	Cell drugs are injected to kill tumors	NCT04657315
IL13Rα2 CAR-T	IL13Rα2 + CD19	I	Recruiting	CAR-T	NCT04661384
NKG2D CAR-T	NKG2D	I	Not yet recruiting	CAR-T	NCT04717999
CB-NK-TGF-betaR2-/NR3C1-NK	NK cell	I	Recruiting	CAR-NK	NCT04991870
EGFRvIII CAR-T	EGFRvIII	I	Unknown	CAR-T	NCT05063682
NK cell therapy	NK cell	I	Unknown	Increase the infiltration of NK cells	NCT05108012
NKG2D CAR-T	NKG2D	I	Recruiting	CAR-T	NCT05131763
CAR-T-EGFR-IL13Ra2 Cells	IL13Rα2 + EGFR	I	Recruiting	CAR-T	NCT05168423
B7-H3 CAR-T	B7-H3	I	Recruiting	CAR-T	NCT05241392
B7-H3 CAR-T	B7-H3	I	Recruiting	CAR-T	NCT05366179
B7-H3 CAR-T	B7-H3	I	Recruiting	CAR-T	NCT05474378
IL7Ra CAR-T	IL7Ra	I	Not yet recruiting	CAR-T	NCT05577091
CHM-1101 CAR-T	MMP2	I	Recruiting	CAR-T	NCT05627323
CARv3-TEAM-E T cells	EGFRvIII	I	Recruiting	CAR-T	NCT05660369
SC-CAR4BRAIN	B7-H3, EGFR806, HER2 and IL13-zetakine	I	Recruiting	CAR-T	NCT05768880

*CAR* Chimeric antigen receptor; *GBM* Glioblastoma; *NK* Natural killer; *CED* Convection enhanced delivery; *CTL* Cytotoxic T-lymphocytes; *HCT* Hematopoietic cell transplantation; *NSC* Neural stem cells; *rGBM* recurrent GBM; *TMZ* Temozolomide; *TIL* Tumor-infiltrating T Lymphocyte; *RT* Radiotherapy; *MSC* Mesenchymal stem cell; *CD* Cytosine deaminase

The advancement of immunotherapy has broadened the therapeutic landscape for GBM patients. Immunotherapy employing CAR-T technology, commonly known as CAR-T therapy, represents an innovative approach to targeting tumors. This method contains extracting T cells from the patient's blood, modifying them with genetic engineering to give specific antigen recognition domains to T cells, and subsequently reintroducing the modified T cells into the patient to eliminate the tumor [607, 608]. CAR-T can specifically recognize cancer cells, thereby enabling targeted cytotoxicity [609]. To mitigate the risk of CAR-T cells targeting normal cells, TAAs must remain either undetectable or minimally expressed in normal tissues [610]. This strategy is a potential therapy for leukemia as well as certain solid tumors. CAR-T therapy, leveraging specific tumor antigens, has been applied to GBM treatment. IL-13 plays a regulatory component in the responses to inflammation and immunity within the TME by binding to IL13Rα1, and it also interacts with the high-affinity decoy receptor IL13Rα2 [611, 612]. Notably, research has demonstrated abundant expression of the IL-13 receptor in GBM patients, with minimal binding sites in normal individuals, rendering it a potential target for CAR-T therapy in GBM [613]. In GBM, IL13Rα2 is related to aggressiveness and worse outcomes. Studies have indicated that CAR-T cells, transfected with human anti-IL13Rα2 CAR and mouse anti-IL13Rα2 CAR, exhibit enhanced expansion capabilities in T cells and more effective inhibitory in GBM growth [614]. Noteworthy clinical outcomes have been observed, such as increased immune cells and cytokines in CSF of patients with rGBM following IL13Rα2-CAR-T therapy, leading to subsidence of cancer cells in the spinal canal and spine [615]. Additionally, investigations have demonstrated favorable tolerance and anti-tumor responses in patients with rGBM treated with intracranial infusion of IL13Ra2 CAR-T [616]. Approximately 40% of newly diagnosed GBM cases exhibit EGFR expression and amplification. Notably, about 50% of GBM patients with EGFR amplification harbor the constitutively active EGFRvIII oncogenic variant, which is characteristically low or absent in normal tissues. This unique expression profile renders EGFRvIII a practical, feasible, and safe therapeutic target for GBM [617, 618]. In a research conducted by Rourke et al. in 2017, CAR-T targeting EGFRvIII in ten patients with EGFRvIII^+^ rGBM was found nonsignificant in prognosis [619]. However, post-surgery observations in seven patients revealed increased CAR-T cells in tumor-infiltrating area. Concurrently, elevations in Tregs were noted, accompanied by heightened expression of inhibitory molecules like PD-L1, IL-10, IDO, and TGF-β. Upregulation of these immunosuppressive factors in the TME led to continuous loss of the EGFRvIII antigen, resulting in diminished CAR-T efficacy. Furthermore, adoptive transfer of CAR-T cells in 18 patients, previously subjected to MDSC and Treg depletion through chemotherapy and IL-2 infusion to support CAR-T cell expansion demonstrated prolonged durability of CAR-T cells but lacked objective responses [620, 621]. These findings suggest that CAR-T targeting EGFRvIII induces a supplementary immune response in the TME. Consequently, it is implied that EGFRvIII CAR-T therapy may exhibit enhanced effectiveness when utilized in conjunction with other immunotherapies to potentiate the anti-tumor immune response or reprogram the TIME. Additionally, EPHA2 is frequently overexpressed within GBM and is correlated with prognosis [622]. Targeting EPHA2 with CAR-T therapy can facilitate IL-2 and IFN-γ secretion, exhibiting significant cytotoxicity against malignant cells as well as extending mice outcomes [623, 624].

Given limited success observed with CAR-T-related therapies, the exploration of other immune cells within the GBM microenvironment holds significant promise for advancing effective immunotherapy strategies [625]. Macrophages, an integral role in innate immune system, can proficiently infiltrate tumors, engulf and eliminate abnormal cells, uptake antigens, and present them to T cells [626]. These distinctive attributes underscore the potential of CAR expression on macrophages to enhance targeting and represent a viable avenue for immunotherapeutic interventions. A noteworthy study has reported the successful generation of CAR macrophages (CAR-MΦ) through the utilization of a cavity-injectable nanoporter-hydrogel system, demonstrating efficacy in preventing GBM recurrence [627]. These engineered CAR-MΦ exhibit a remarkable ability to locate phagocytic GSCs, impeding their residual presence. This mechanism stimulates an adaptive anti-tumor immune response within the TME. Significantly, these CAR-MΦ have demonstrated the capacity to lead to enduring anti-tumor immunity, effectively preventing the recurrence of GBM post-surgery.

NK cells can be subject to genetic engineering to express CAR, resulting in increased protein levels in GBM, a condition associated with a worse prognosis. Following the stereotactic injection of Erb-B2 receptor tyrosine kinase 2 (ErbB2)-specific CAR NK cells into the tumor, a notable extension of asymptomatic survival time was observed, extending from 73 days to 200.5 days. CAR-NK therapy exhibited curative effects in immunocompetent mice, curing a significant proportion of subcutaneous tumor-bearing and GBM-bearing mice while enhancing the innate immune system to resist tumors. This, in turn, led to the acquisition of enduring anti-tumor immune responses [628]. Moreover, CD155/CD112, through interaction with DNAX accessory molecule-1 (DNAM-1) and TIGIT on NK cells, exerts immunomodulatory effects and enhances their expression in GBM. These findings position CAR-NK as a potential therapy in GBM [234]. Although neutrophils possess an efficient ability to traverse physiological barriers in response to pathogens, their short lifespan and resistance to genome editing have constrained their broader application in immunotherapy. Chang et al. employed gene editing technology to induce CAR neutrophils, incorporating specific gamma signaling domains produced by human pluripotent stem cells, demonstrating a favorable anti-GBM effect. CAR-neutrophils, thus engineered, can deliver and release nano drugs that influence the TME without inducing additional inflammation, providing a more targeted approach to GBM treatment [629]. While exploring CAR non-T cell treatment in GBM is still in the early stage, preclinical findings indicate an unlimited potential for applying this strategy, making it a promising avenue for future research. As with various other treatments, combination therapy involving CAR appears to be a prevailing trend in GBM treatment.

A parallel therapeutic strategy is the application of bispecific T cell engagers (BiTE), which involves linking an agonist antibody fragment targeting the TCR complex CD3ε to a tumor antigen [630] and a gene-fusion antibody fragment promoting the crosstalks in T cells and target cells, like cancer cells. This design establishes an artificial immune synapse to enhance killing target cells by T cells [631, 632]. The current landscape of BiTE therapy in GBM is an emerging area of investigation. The promising outcomes observed in BiTE therapy targeting IL13Rα2 [633, 634], EGFRvIII [635], EGFR [633], and Fn14 [636], along with notable efficacy in GBM animal models, provide a robust foundation for subsequent clinical translation studies.

### Application of glioblastoma vaccine therapy

Vaccine therapy, as the earliest developed form of immunotherapy, has emerged as a crucial approach for researchers to modulate the immune system, enhancing local immune responses to achieve therapeutic effects [637]. It holds longstanding promise for instigating potent anti-tumor immunity, directing cytotoxicity toward tumors while preserving normal tissue, and establishing durable immune memory capable of monitoring tumor recurrence [638, 639]. In the spectrum of immunotherapy strategies for GBM, vaccine therapy stands out as a method to target tumor antigens, surmount the internal immunosuppressive milieu within the tumor, and augment the immune response against the tumor. Multiple TAAs have been identified in GBM, some of which present as promising candidates for vaccine-directed immunotherapy [640]. These cancer vaccines are meticulously crafted to instigate the development of long-term memory in tumor-specific effector T cells, aiming to eradicate cancer cells and forestall tumor recurrence [640, 641]. Table 7 provides an overview of ongoing clinical trials exploring various vaccine therapies for GBM, encompassing peptide vaccines, cell vaccines, mRNA vaccines, and more.

**Table 7:** Current clinical trials based on tumor-associated vaccine for glioblastoma

Agents	Targets	Phase	Status	Mechanism	Trial ID
Autologous tumor cell vaccine + GM-CSF	T cell	II	Completed	Enhance anti-tumor immunity	NCT00003185
DCVax-L + RT/TMZ	DC vaccine	III	Active	Establish anti-tumor immunity	NCT00045968
Tumor lysate-pulsed DC vaccine	DC vaccine	I	Completed	Establish anti-tumor immunity	NCT00068510
540–548 peptide vaccine + Sargramostim	TRET	I	Completed	Enhance anti-tumor immunity	NCT00069940
HSPPC-96	HSPPC-96	I/II	Completed	Establish anti-tumor immunity	NCT00293423
Autologous DC + RT/TMZ	DC vaccine	II	Completed	Establish anti-tumor immunity	NCT00323115
Tumor lysate-pulsed DC vaccine	DC vaccine	II	Completed	Establish anti-tumor immunity	NCT00576537
Autologous DC + RT/TMZ	DC vaccine	I	Completed	Establish anti-tumor immunity	NCT00576641
TAA + Autologous DC vaccine	DC vaccine	I	Completed	Modulate anti-tumor immunity	NCT00612001
PEP-3-KLH conjugate vaccine + Daclizumab + TMZ	KLH + CD25	I	Completed	Establish anti-tumor immunity, especially inhibit Treg	NCT00626015
RNA-loaded dendritic cell vaccine + Basiliximab	DC vaccine + IL2R	I	Completed	Modulate anti-tumor immunity	NCT00626483
CMV pp65-LAMP DC and CMV-ALT	DC vaccine	I	Completed	Establish anti-tumor immunity	NCT00639639
PEP-3 vaccine + TMZ + Sargramostim	KLH + GM-CSF	II	Completed	Enhance anti-tumor immunity	NCT00643097
CMV-ALT + CMV-DCs	DC vaccine	I	Completed	Modulate anti-tumor immunity	NCT00693095
AP12009	TGF-β2	III	Terminated	mRNA activates anti-tumor immunity	NCT00761280
DC vaccine with mRNA from GSC	DC vaccine	I/II	Completed	Establish anti-tumor immunity	NCT00846456
BTSC mRNA-loaded DCs	DC vaccine	I	Completed	Establish anti-tumor immunity	NCT00890032
HSPPC-96 + TMZ	HSPPC-96	II	Completed	Establish anti-tumor immunity	NCT00905060
Tumor lysate-pulsed DC vaccine	DC vaccine	II	Completed	Establish anti-tumor immunity	NCT01006044
DC + Imiquimod	DC vaccine + TLR7	I	Completed	Modulate anti-tumor immunity	NCT01171469
Tumor lysate-pulsed DC vaccine + ICLC	DC vaccine	II	Active	Establish anti-tumor immunity	NCT01204684
Trivax + RT/TMZ	DC vaccine	I	Completed	Establish anti-tumor immunity	NCT01213407
ISA-51/Survivin Peptide Vaccine	Survivin	I	Completed	Establish anti-tumor immunity	NCT01250470
ICT-107	DC vaccine	II	Completed	Establish anti-tumor immunity	NCT01280552
TVI-Brain-1	T cell	II	Completed	Enhance anti-tumor immunity	NCT01290692
TTRNA-xALT + TTRNA-DCs	T cell + DC	II	Active	Enhance anti-tumor immunity	NCT01326104
CDX-110 + GM-CSF	EGFRvIII	III	Completed	Enhance anti-tumor immunity	NCT01480479
CDX-110 + GM-CSF + Bevacizumab	EGFRvIII + VEGFR	II	Completed	Enhance anti-tumor immunity	NCT01498328
DEC-205/NY-ESO-1 Fusion Protein CDX-1401	NY-ESO-1	I	Completed	Establish anti-tumor immunity	NCT01522820
GSC DC vaccine + RT	DC vaccine	II	Unknown	Establish anti-tumor immunity	NCT01567202
WT2725	WT1	I	Completed	Establish anti-tumor immunity	NCT01621542
DC vaccine, allogeneic hematopoietic stem cells, cytotoxic lymphocytes	Multiple vaccine	II/III	Unknown	Modulate anti-tumor immunity	NCT01759810
Tumor lysate-pulsed DC vaccine + Imiquimod	DC vaccine + TLR7	I	Completed	Modulate anti-tumor immunity	NCT01808820
HSPPC-96 + Bevacizumab	HSPPC-96 + VEGFR	II	Terminated	Modulate anti-tumor immunity	NCT01814813
ERC1671 + GM-CSF + Bevacizumab	Tumor lysate and VEGFA	II	Active	Modulate anti-tumor immunity	NCT01903330
IMA 950	Multiple vaccine	I/II	Completed	Modulate anti-tumor immunity	NCT01920191
Lysate-Pulsed Autologous DC Vaccine + TMZ	DC vaccine	I	Active	Establish anti-tumor immunity	NCT01957956
GSC DC vaccine + RT/TMZ	DC vaccine	I	Completed	Establish anti-tumor immunity	NCT02010606
ICT-121 DC vaccine	DC vaccine + CD133	I	Completed	Modulate anti-tumor immunity	NCT02049489
SL-701 + Bevacizumab	Multiple vaccine	I/II	Completed	Modulate anti-tumor immunity	NCT02078648
APVAC1/2 vaccine + Poly-ICLC + GM-CSF	Personal antigen	I	Completed	Modulate anti-tumor immunity	NCT02149225
NeoAntigen vaccine + Pembrolizumab/MK-3475 + RT/TMZ	TAA and PD-1	I	Recruiting	Modulate anti-tumor immunity	NCT02287428
Autologous DC + Decitabine/Hiltonol	NY-ESO-1, MAGE-A1 and MAGE-A3	I/II	Terminated	Modulate anti-tumor immunity	NCT02332889
CMV pp65-LAMP DC vaccine + TMZ/Basiliximab	DC vaccine and IL-2R	II	Completed	Establish anti-tumor immunity	NCT02366728
SVN53-67/M57-KLH Peptide Vaccine	Survivin	II	Active	Modulate anti-tumor immunity	NCT02455557
pp65-shLAMP DC with GM-CSF	DC vaccine	II	Active	Establish anti-tumor immunity	NCT02465268
DC + Nivolumab	DC vaccine + PD-1	I	Completed	Modulate anti-tumor immunity	NCT02529072
ICT-107	TAA	III	Suspended	Modulate anti-tumor immunity	NCT02546102
WT1 mRNA DC vaccine + TMZ	DC vaccine	I/II	Recruiting	Establish anti-tumor immunity	NCT02649582
Personalized cellular vaccine	DC vaccine	I	Completed	Establish anti-tumor immunity	NCT02709616
HSPPC-96 + RT	HSPPC-96 in children	I	Terminated	Modulate anti-tumor immunity	NCT02722512
GSC DC vaccine	DC vaccine in rGBM	I	Completed	Establish anti-tumor immunity	NCT02820584
PEP-CMV + TMZ	Peptide vaccine	I	Terminated	Modulate anti-tumor immunity	NCT02864368
Autologous DC + Tumor lysate antigen Vaccine	Multiple vaccine	II	Withdraw	Modulate anti-tumor immunity	NCT03014804
HSPPC-96 + Pembrolizumab + TMZ	HSP-96 and PD-1	II	Completed	Modulate anti-tumor immunity	NCT03018288
DSP-7888 + Bevacizumab	WT1 peptide vaccine + PD-1	III	Completed	Modulate anti-tumor immunity	NCT03149003
MTA-based peptide vaccine + ICLC + TTField	Peptide vaccine	I	Active	Establish anti-tumor immunity	NCT03223103
Tumor Lysate-Pulsed Autologous DC Vaccine	DC vaccine	I	Completed	Establish anti-tumor immunity	NCT03360708
VBI-1901 + GM-CSF + Carmustine/Lomustine	CMV vaccine	I/II	Recruiting	Modulate anti-tumor immunity	NCT03382977
Immune adjuvants + RT	Multiple vaccine	I	Unknown	Modulate anti-tumor immunity	NCT03392545
Tumor lysate-pulsed DC vaccine + RT/TMZ	DC vaccine	II	Recruiting	Establish anti-tumor immunity	NCT03395587
NeoVax + Nivolumab + Ipilimumab	TAA + PD-1 + CTLA-4		Completed	Modulate anti-tumor immunity	NCT03422094
AV-GBM-1	DC vaccine	II	Active	Establish anti-tumor immunity	NCT03400917
INO-5401 + INO-9012 + Cemiplimab + RT/TMZ	WT1, PSMA, TERT, IL12 and PD-1	I/II	Active	mRNA activates anti-tumor immunity	NCT03491683
hTERT/GSC DC vaccine + TMZ	DC vaccine	II/III	Active	Establish anti-tumor immunity	NCT03548571
CMV-DCs with GM-CSF	DC vaccine	I	Completed	Establish anti-tumor immunity	NCT03615404
GP96 vaccine + RT/TMZ	Peptide vaccine	II	Recruiting	Modulate anti-tumor immunity	NCT03650257
IMA950/Poly-ICLC + Pembrolizumab	HLA-A2 peptide vaccine and PD-1 in rGBM	I/II	Active	Modulate anti-tumor immunity, especially CD8^+^ T cell	NCT03665545
CMV pp65-LAMP mRNA-pulsed autologous DCs	DC vaccine	II	Active	Modulate anti-tumor immunity	NCT03688178
VXM01 + Avelumab	VEGFR-2 + PD-L1	I/II	Active	mRNA activates anti-tumor immunity	NCT03750071
Temferon	HSPC with CD34, IFN-α2 in nonme-MGMT	I/II	Recruiting	Enhance anti-tumor immunity	NCT03866109
Tumor lysate-pulsed DC vaccine + RT/TMZ + Nivolumab and Ipilimumab	DC vaccine and PD-1, CTLA-4 in children rGBM	I/II	Recruiting	Eliminate the Treg	NCT03879512
Immunomodulatory DC vaccine to target DIPG and GBM	DC vaccine	I	Unknown	Establish anti-tumor immunity	NCT03914768
V-Boost	Oral TAA	II	Unknown	Modulate anti-tumor immunity	NCT03916757
CMV pp65-LAMP DC vaccine with GM-CSF	DC vaccine in nonmeMGMT	II	Terminated	Establish anti-tumor immunity	NCT03927222
Montanide ISA 51 + Pembrolizumab/SurVaxM	Survivin peptide vaccine and PD-1	II	Active	Modulate anti-tumor immunity	NCT04013672
Personalized neoantigen DNA vaccine	TAA	I	Active	mRNA activates anti-tumor immunity	NCT04015700
Autologous DC	DC vaccine	II	Recruiting	Establish anti-tumor immunity	NCT04115761
EO2401 + Nivolumab/Bevacizumab	IL13Rα2, BIRC5, FOXM1 and PD-1/VEGFA	I/II	Active	Modulate anti-tumor immunity, especially CD8^+^ T cell	NCT04116658
Tumor lysate-pulsed DC vaccine + Pembrolizumab + ICLC	DC vaccine and PD-1	I	Recruiting	Modulate anti-tumor immunity	NCT04201873
UCPVax + TMZ	TERT vaccine	II	Active	Modulate anti-tumor immunity, especially CD4^+^ T cell	NCT04280848
ADCTA-SSI-G1	DC vaccine in rGBM	III	Unknown	Modulate anti-tumor immunity	NCT04277221
Tumor lysate-pulsed DC vaccine with IL12 + TMZ	DC vaccine in rGBM	I/II	Recruiting	Establish anti-tumor immunity	NCT04388033
IGV-001 + TMZ	IGF-1R	II	Recruiting	mRNA activates anti-tumor immunity	NCT04485949
Autologous DC vaccine + TMZ	DC vaccine	II	Recruiting	Establish anti-tumor immunity	NCT04523688
TH-1 DC vaccine + TMZ	DC vaccine	I	Recruiting	Modulate anti-tumor immunity	NCT04552886
RNA-lipid Particle	LAMP in children	I	Recruiting	mRNA activates anti-tumor immunity	NCT04573140
GBM6-AD + hP1A8	Tumor lysate and CD200AR-L	I	Active	Modulate anti-tumor immunity, especially CD8^+^ T cell	NCT04642937
Tumor lysate-pulsed DC vaccine + RT/TMZ	DC vaccine	I/II	Recruiting	Establish anti-tumor immunity	NCT04801147
H3K27M peptide vaccine + Atezolizumab	Peptide vaccine + PD-1	I	Recruiting	Modulate anti-tumor immunity	NCT04808245
GLIO-XS15 + RT/TMZ	Peptide vaccine	I	Recruiting	Modulate anti-tumor immunity	NCT04842513
GSC DC vaccine + Carilizumab	DC vaccine and PD-1	II	Recruiting	Modulate anti-tumor immunity	NCT04888611
Autologous DC + multiple neoantigen peptides + RT/TMZ	Multiple vaccine	I	Recruiting	Modulate anti-tumor immunity	NCT04968366
CMV pp65-LAMP DC with GM-CSF	DC vaccine	I	Active	Establish anti-tumor immunity	NCT04963413
AV-GBM-1 + Autologous monocytes	DC vaccine	III	Not yet recruiting	Modulate anti-tumor immunity	NCT05100641

*RT* Radiotherapy; *TMZ* Temozolomide; *DC* Dendritic cell; *TAA* Tumor-associated antigen; *Treg* regulatory T cell; *GSC* Glioblastoma stem cell; *ICLC* Interstitial Cajal-like cells; *GBM* Glioblastoma; *rGBM* relapsed GBM; *TTField* Tumor Treating Fields; *HSPC* Hematopoietic stem/progenitor cells; *MGMT* Methyl guanine methyl transferase; *nonme-MGMT* non-methylated MGMT; *DIPG* Diffuse intrinsic pontine glioma

GBM-associated TAAs identified thus far encompass but are not confined to IDH1, HSP, Wilms tumor protein (WT1), survivin, IL13Ra2, EGFRvIII, and IL-4 [642–644]. EGFRvIII, expressed heterogeneously in approximately one-third of GBM patients, is absent in normal tissues and serves as an independent adverse prognostic marker, presenting a crucial target for antitumor immunotherapy [645]. Investigations have demonstrated that the introduction of Rindopepimut, a 14-amino acid peptide vaccine targeting EGFRvIII, significantly extended patients with GBM prognosis, particularly combined with TMZ, showcasing the vaccine's remarkable efficacy [646]. Combining Rindopepimut with the VEGFR inhibitor bevacizumab has demonstrated prolonged progression-free survival (PFS) in rGBM [619]. Survivin, an anti-apoptotic protein prevalent in brain tumors, is associated with a poorer prognosis and is scarcely found in normal tissues, rendering it an appealing vaccine target. SurVaxM, a survivin-targeted peptide vaccine, received orphan drug designation from the FDA owing to its capacity to stimulate T cell immunity and inhibit the survivin pathway. Clinical research has indicated that SurVaxM can enhance the PFS of patients with survivin-positive rGBM [647]. DCs, as the most critical type of antigen-presenting cell (APC), are essential for stimulating primary T-cell proliferation. As for brain tumor immunotherapy, a significant focus is placed on DC vaccines, involving the in vitro production of autologous DCs pretreated with tumor antigens, which are reintroduced into patients as immunotherapy [648]. While autologous cell vaccines, particularly DC vaccines, are intricate and costly, they have demonstrated the capacity to elicit robust immune responses [649]. DCVax-L, an autologous cell vaccine comprising DCs pulsed with autologous tumor lysate to stimulate the immune response, has exhibited promising outcomes. Patients with MES gene expression characteristics treated with DCVax-L displayed higher CD8^+^ T cell infiltration to TME, significantly extending outcomes compared to patients with other gene expression profiles in GBM [650]. Cytomegalovirus (CMV), a double-stranded DNA virus, has been detected in various tumor types, including GBM [651]. Persistent chronic inflammation and immunosuppression in GBM can reactivate CMV, offering a potential therapeutic avenue [652]. The CMV phosphoprotein 65 (pp65) RNA, expressed in over 90% GBM but not in the normal tissue, serves as a novel target [651]. Targeting CMV pp65 mRNA-pulsed DC vaccines has induced robust anti-tumor immunity by upregulating CCL3. Deposing with some antigen, like tetanus/diphtheria (Td) toxoid, enhances tumor-antigen-specific DC infiltration into draining lymph nodes, related to a notable improvement in the OS of GBM patients [653]. Vaccine therapy for GBM holds promise in preclinical and early clinical assessments. Combined strategies, including immune checkpoint blockade (ICB), Treg depletion, and enhanced DC migration, may synergize with tumor-specific vaccines to enhance patient outcomes. The future of GBM vaccine therapy may involve combinatorial approaches that integrate the identification of tumor-specific antigens with vaccines and block immunosuppressive pathways, thereby mitigating the strength and duration of antitumor immunity in GBM patients [654].

### Oncolytic viruses, immunotoxins, and antibody-coupled drug therapy

Immunotherapy, encompassing strategies such as ICI, cytokine-based therapies, vaccine therapies, T cell therapies, and viral therapies designed to specifically target tumors, has emerged as a focal point in anti-tumor research [655]. Oncolytic viruses (OVs) operate primarily through two mechanisms: some infect and selectively replicate within tumor cells, while others involve the introduction of transgenes promoting anti-tumor effects into non-replicating viruses [656]. Current research aims to express novel transgenes in viruses, preserving their replication and lytic capabilities to enhance tumor clearance and patient survival. Several oncolytic viruses are undergoing clinical development, like herpes simplex virus (HSV), adenovirus (ADV), vaccinia virus, coxsackievirus, measles virus (MV), poliovirus (PV), reovirus, and Newcastle disease virus (NDV), with many in early clinical trials [657–659]. However, like other treatments, oncolytic virus therapy encounters challenges in patient selection. Identifying patients likely to respond to oncolytic virus treatment remains challenging, and reliable biomarkers and predictive factors for OV therapy response are yet to be fully elucidated [660]. Table 8 provides an overview of current clinical trials related to virotherapy strategies for GBM, encompassing OVs, immunotoxins (ITs), and antibody–drug conjugates (ADCs), among others.

**Table 8:** Current clinical trials based on OV, IT and ADC for glioblastoma

Agents	Targets	Phase	Status	Mechanism	Trial ID
H5.010RSVTK	Adenovirus + Aglatimagene	I	Completed	Directly kill tumors and activate the immune system	NCT00002824
G207	HSV-1	I/II	Completed	Directly kill tumors and activate the immune system	NCT00028158
IL13-PE38QQR	IL13 + Pseudomonas exotoxin A	III	Completed	Tumoricidal activity	NCT00076986
TP-38	EGFR,TGF-α and Pseudomonas exotoxin-38	II	Completed	Tumoricidal activity	NCT00104091
G207	HSV-1	I	Completed	Directly kill tumors and activate the immune system	NCT00157703
Seneca Valley Virus	Seneca Valley Virus	I	Unknown	Directly kill tumors and activate the immune system	NCT00314925
MV-CEA	Measles virus + CEA	I	Completed	Directly target kill tumors and activate the immune system	NCT00390299
REOLYSIN®	Reovirus	I	Completed	Directly kill tumors and activate the immune system	NCT00528684
AdV-tk	Adenovirus + Aglatimagene	II	Completed	Directly kill tumors and activate the immune system	NCT00589875
AdV-tk + RT/TMZ	HSV thymidine kinase gene	I	Completed	Directly kill tumors and activate the immune system	NCT00634231
GliAtak	Adenovirus + Aglatimagene	I	Completed	Directly kill tumors and activate the immune system	NCT00751270
PRX321 + CED	IL4 + Pseudomonas exotoxin A	II	Withdrawn	Tumoricidal activity	NCT00797940
ADV-TK/GCV + Chemotherapy	Adenovirus + Aglatimagene	II	Completed	Directly kill tumors and activate the immune system	NCT00870181
New Castle Disease Virus	OV	I/II	Withdrawn	Directly kill tumors and activate the immune system	NCT01174537
H-1PV	H-1 parvovirus	I/II	Completed	Directly kill tumors and activate the immune system	NCT01301430
PVSRIPO	Polio/Rhinovirus	I	Completed	Directly kill tumors and activate the immune system	NCT01491893
delta-24-RGD adenovirus + CED	Adenovirus	I/II	Completed	Directly kill tumors and activate the immune system	NCT01582516
Ad-hCMV-TK and Ad-hCMV-Flt3L	Flt3L	I	Completed	Directly target, kill tumors and activate the immune system	NCT01811992
DNX-2401 + TMZ	Adenovirus	I	Completed	Directly kill tumors and activate the immune system	NCT01956734
HSV-1716 + Dexamethasone	HSV-1	I	Terminated	Directly kill tumors and activate the immune system	NCT02031965
M032	HSV-1	I	Active	Directly kill tumors and activate the immune system with IL12	NCT02062827
DNX-2401 + IFN-γ	Adenovirus	I	Completed	Directly kill tumors and activate the immune system	NCT02197169
Depatuxizumab mafodotin	ADC target EGFR	II	Completed	Antibody conjugated drugs that target EGFR	NCT02343406
Toca 511 + Toca FC + TMZ + Bevacizumab	Retroviral Replicating Vector	II/III	Terminated	Directly kill tumors and activate the immune system	NCT02414165
G207	HSV-1	I	Active	Directly kill tumors and activate the immune system	NCT02457845
LY3076226	ADC target FGFR3	I	Completed	Antibody conjugated drugs that target FGFR3	NCT02529553
Toca 511 + Toca FC	Retroviral Replicating Vector	I	Terminated	Directly kill tumors and activate the immune system	NCT02576665
EGFR(V)-EDV-Dox	ADC target EGFR	I	Unknown	Doxorubicin kill tumors and activate immunity	NCT02766699
DNX-2401 + Pembrolizumab	Genetically modified oncolytic adenovirus + PD-1	II	Completed	Directly kill tumors and activate the immune system	NCT02798406
MDNA55 + CED	IL4 + Pseudomonas aeruginosa exotoxin A	II	Completed	Tumoricidal activity	NCT02858895
PVSRIPO	Polio/Rhinovirus in rGBM	II	Active	Directly kill tumors and activate the immune system	NCT02986178
PVSRIPO + CED	Polio/Rhinovirus	I	Unknown	Directly kill tumors and activate the immune system	NCT03043391
NSC loaed with an oncolytic adenovirus	Adenovirus	I	Completed	Directly kill tumors and activate the immune system	NCT03072134
rQNestin	HSV-1	I	Recruiting	Directly kill tumors and activate the immune system	NCT03152318
TG6002 and 5-flucytosine	Oncolytic vaccinia virus	I/II	Unknown	Directly kill tumors and activate the immune system	NCT03294486
AdV-tk + RT/TMZ + Nivolumab	HSV thymidine kinase gene + PD-1	I	Active	Directly kill tumors and activate the immune system	NCT03576612
Ad-RTS-hIL-12 + Veledimex + Nivolumab	Adenovirus + PD-1	I	Completed	Directly kill tumors and activate the immune system with IL12	NCT03636477
C134	HSV-1 in IRS-1	I	Recruiting	Directly kill tumors and activate the immune system	NCT03657576
DNX-2440	Adenovirus	I	Terminated	Directly kill tumors and activate the immune system	NCT03714334
Oncolytic Adenovirus Ad5-DNX-2401	Adenovirus	I	Recruiting	Directly kill tumors and activate the immune system	NCT03896568
G207	HSV-1	I	Recruiting	Directly kill tumors and activate the immune system	NCT03911388
PVSRIPO + Atezolizumab	Polio/Rhinovirus and PD-1	I/II	Withdrawn	Directly kill tumors and activate the immune system	NCT03973879
Ad-RTS-hIL-12 + Veledimex + Cemiplimab-Rwlc	Adenovirus	II	Completed	Directly kill tumors and activate the immune system with IL12	NCT04006119
D2C7-IT + Atezolizumab	EGFR, EGFRvIII and PD-1	I	Active	Tumoricidal activity and immunotoxins induce secondary the activation of T cells	NCT04160494
VB11 + Bevacizumab	Adenovirus + VEGFA	II	Active	Target and damage the blood vessels	NCT04406272
Lerapolturev + Pembrolizumab	Small RNA OV target CD155 + PD-1	II	Active	Directly kill tumors and activate the immune system	NCT04479241
G207 + RT	HSV-1 in children	II	Not yet recruiting	Directly kill tumors and activate the immune system	NCT04482933
D2C7-IT + 2141-V11	EGFR, EGFRvIII and CD40	I	Recruiting	Tumoricidal activity and immunotoxins induce secondary the activation of T cells	NCT04547777
C5252	HSV-1 with IL12/PD-1	I	Not yet recruiting	Directly kill tumors and modulate the immune system	NCT05095441

*OV* Oncolytic virus; *IT* Immunotoxin; *ADC* Antibody–drug conjugate; *HSV* Herpes simplex virus; *CEA* Carcino-embryonic antigen; *RT* Radiotherapy; *TMZ* Temozolomide; *CED* Convection enhanced delivery; *GBM* Glioblastoma; *rGBM* recurrent GBM; *NSC* Neural stem cells

CCL5, an inflammatory chemokine that facilitates immune cell chemotaxis through interaction with CCR1/CCR5, undergoes methylation-induced silencing in the progression of solid tumors [661]. Consequently, restoring or augmenting CCL5 expression is a prospective therapeutic strategy for overcoming the TIME in GBM. However, the inherent challenges of its short half-life, delivery to the TME, and potential off-target toxic effects limit its efficacy in tumor therapy. GBM cells infected with oncolytic HSV, targeting both EGFR and CCL5 receptors, exhibit elevated and sustained levels of CCL5 in the TME. This elevation enhances adaptive and innate immune cell infiltration. Furthermore, acting as an IgG1 anti-EGFR monoclonal antibody, it activates macrophage antibody-dependent cellular phagocytosis (ADCP) and NK cells through antibody-dependent cellular cytotoxicity (ADCC), thereby reducing EGFR signaling in cancer cells [662, 663]. This comprehensive strategy significantly prohibits cancer growth and prolongs the mice's prognosis. The ECM contributes to tumor progression by interacting with cancer cells and stromal components within the TME [664]. In GBM, the tumor ECM, consisting of proteins like collagen, fibronectin, and laminin, along with non-proteins such as hyaluronan (HA), plays a critical role [665, 666]. HA regulates cancer cell proliferation and invasion and affects chemotherapy activity by binding to CD44 and receptor for hyaluronic acid-mediated motility (RHAMM) [665]. ICOVIR17, an ADV expressing hyaluronidase, is employed to treat GBM-bearing mice [667]. This virus degrades HA, disrupting the immunosuppressive microenvironment by inhibiting the NF-κB signaling pathway. Consequently, this approach increases CD8^+^ T cells and macrophages infiltrating into tumors, ultimately extending mice prognosis [666]. Moreover, oncolytic HSV-1 G207 demonstrates significant efficacy in prolonging the median OS of GBM patients. As a neurophilic virus, G207 is well-suited for targeting GBM. Its ability to bypass the BBB through intratumoral inoculation enables direct infection and lysis of tumor cells. This, in turn, reverses tumor immune escape, enhances the cross-presentation of tumor antigens, and enhances the immune system resisting tumors [662, 668, 669].

ITs represent a class of therapeutic agents comprising targeted peptides, typically antibodies or antibody fragments, coupled with peptide toxins sourced from plants or bacteria [670]. Some toxins possess potent cytotoxic properties, inducing apoptosis and inhibiting protein synthesis in the cytoplasm. Consequently, ITs are recognized as crucial agents in cancer treatment and infection prevention [671]. Several pseudomonas exotoxins (PE) based ITs have undergone exploration and evaluation [672]. After recognition and binding to the target antigen, ITs undergo internalization through endocytosis mediated by receptors. The functional domain in PE then catalyzes elongation factor-2 (EF2) with ADP-ribosylation in cytoplasm. This process induces the arrest in protein synthesis, ultimately inducing cell death [673]. In the context of GBM, IL-13R has been identified by the majority of GBM cells and samples obtained from surgically resected patients [674]. Particularly, the IL13Rα2 chain, a principal binding and internalization component of IL-13, is expressed in approximately 80% of GBM tumor specimens but is minimally expressed in normal brain tissues [675]. IL13-PE38QQR is IL-13 with a truncated form of *Pseudomonas aeruginosa* exotoxin A (PE38QQR). This compound induces cytotoxicity through inhibiting protein synthesis, causing cell apoptosis and death [676–678]. Convection-enhanced delivery (CED) in CNS of IL13-PE38QQR has demonstrated significant efficacy in extending the median OS of patients with rGBM [678]. This targeted therapeutic approach capitalizes on the specific expression of IL13Rα2 in GBM tumor specimens, underscoring its potential as a promising treatment strategy for this aggressive form of brain cancer. Furthermore, when combined with concurrent 5 Gy irradiation, the cytotoxicity to GBM cells was significantly enhanced [679]. This suggests that IT-targeted IL-13R, in combination with other modalities such as RT, holds promise for enhancing treatment outcomes in GBM patients. Additionally, intratumoral injection of EGFRvIII IT has demonstrated the eradication of tumors in a GBM mouse model. The down-regulation of MGMT mediated by IT further sensitizes tumor cells to TMZ [680]. D2C7-IT (D2C7) represents a recombinant antibody fragment-based IT targeting EGFR and EGFRvIII, two predominant driver oncogenes in GBM [135]. Delivery of D2C7 via CED leads to direct tumor cell death and facilitates CD4^+^ and CD8^+^ T cells, triggering secondary immune responses [681]. While D2C7 monotherapy has demonstrated prolonged survival and promoted disease control in some patients, its efficacy is constrained by the potent immunosuppressive microenvironment in GBM [682]. Combined therapy with targeted CD40 has shown the potential to enhance the response of GBM to D2C7 treatment. CD40, a costimulatory factor in TNF receptor superfamily, is highly expressed in GBM [683]. The combination of D2C7 and anti-CD40 cytotoxic immunotherapy activates microglia and TAMs, creates a pro-inflammatory TME, inhibits exhaustion of CD8^+^ TILs, and increases tumor antigen-specific CD8^+^ TILs. This comprehensive approach has demonstrated prolonged survival and development of a long-term anti-tumor immune response in mice bearing GBM. Phase I clinical trials for this combination therapy have been initiated [684].

ADCs represent an advancing anti-cancer drug, combined with targeting precision of monoclonal antibodies and the anti-tumor effects in cytotoxic drugs [630, 685]. Currently, more than 40 ADCs have entered clinical trials, including FDA-approved examples like Adcetris and Kadcyla, used in treating CD30-overexpressing Hodgkin lymphoma and human epidermal growth factor receptor 2 (HER2)-overexpressing breast cancer, respectively [686, 687]. Application in ADCs is also gaining prominence in the treatment of GBM. AMG595 combines the highly selective anti-EGFRvIII antibody with mertansine (DM1), an anti-tubulin agent, through a non-cleavable linker. This ADC combines with the membrane and gets into the endo-lysosomal pathway of EGFRvIII^+^ cells, inducing mitotic arrest in tumor cells and resulting in regression of GBM [688, 689]. Galectin 3 binding protein (LGALS3BP) is vital in regulating stroma-tumor interactions and is among the most abundant surface components in tumor-derived extracellular vesicles [690]. Plasma vesicle LGALS3BP levels are related to the grade and progression of glioma [691]. Targeting LGALS3BP with an ADC has proven effective in inhibiting GBM cell growth in vivo, inducing a noticeable improvement in the survival time of mice [692]. CD97 is expressed in various immune system lineages. It is vital in inflammatory responses in a range of liquid (leukemia) and solid (ovarian, esophageal, breast, stomach, colon, pancreatic, thyroid, prostate, hepatocellular) malignancies, including GBM [693–695]. CD97 is associated with cell proliferation, brain invasion, and tumor metabolism in GBM [696]. It promotes Warburg metabolism through signaling mechanisms, including receptor cytoplasmic C-terminal phosphorylation, β-arrestin recruitment, and activating MAPK/ERK signal, thereby contributing to tumorigenesis in GBM [697]. The ADC targeting CD97 has demonstrated selective killing of patient-derived GBM cultures while sparing neural stem cells and non-neoplastic human astrocytes. This suggests that a CD97-targeting ADC is a potential treatment in GBM [698].

### The integration of medicine and engineering technology shines brightly in glioblastoma

The convergence of medicine and engineering constitutes an emerging interdisciplinary field that embodies a collaborative and innovative approach, amalgamating medical sciences with engineering technologies [699]. In the context of cancer treatment, this fusion entails the application of biotechnology in tandem with engineering methods to optimize drug delivery and treatment targeting. Table 9 provides an overview of ongoing clinical trials focused on engineering-based treatments for GBM.

**Table 9:** Current clinical trials based on engineering technology for glioblastoma

Agents	Targets	Phase	Status	Mechanism	Trial ID
OncoGel	None	I/II	Terminated	Use a gel to release the drug slowly	NCT00479765
Nanoliposomal CPT-11	Topoisomerase-I	I	Completed	Nanoparticles are used to deliver drugs to improve drug absorption	NCT00734682
Pegylated Liposomal Doxorubicine	None	I/II	Completed	Increase drug absorption and thus increase drug concentration	NCT00944801
Carboplatin + CED	Chemotherapy	I	Withdrawn	Local administration of CED increased effectiveness	NCT01317212
2B3-101 + Trastuzumab	HER2	I/II	Completed	Increase drug absorption and thus increase drug concentration	NCT01386580
MRI-guided Laser Heat Ablation + Doxorubicin	BBB + Topoisomerase-II	I	Completed	Local BBB is destroyed by thermal ablation to improve drug absorption efficiency	NCT01851733
Rhenium Liposome + CED	RT	I/II	Recruiting	Local radioactive substances kill tumors	NCT01906385
SonoCloud + Carboplatin	BBB	I/II	Completed	The BBB is opened by ultrasound to facilitate drug absorption	NCT02253212
MRI-guided laser ablation + MK-3475	BBB + PD-1	I/II	Active	Local BBB is destroyed by thermal ablation to improve drug absorption efficiency	NCT02311582
SGT-53 + TMZ	P53	II	Terminated	Repair of gene mutations in tumors by local liposomal DNA delivery	NCT02340156
Transcranial ExABlate	BBB	None	Unknown	The BBB is opened by ultrasound to facilitate drug absorption	NCT02343991
MRI-guided Laser Heat Ablation + Doxorubicin	BBB + Topoisomerase-II	II	Recruiting	Local BBB is destroyed by thermal ablation to improve drug absorption efficiency	NCT02372409
EGFR(V)-EDV-Dox	EFFR + Topoisomerase-II	I	Unknown	Target specific targets to deliver drugs and activate the immune system	NCT02766699
Myocet®	Topoisomerase-II	I	Completed	Using liposome to improve drug absorption efficiency	NCT02861222
NU-0129	Bcl2L12 in rGBM	I	Completed	Nanoparticles are used to deliver drugs to improve drug absorption	NCT03020017
ExAblate Model 4000 Type 2	BBB	None	Suspended	The BBB is opened by ultrasound to facilitate drug absorption	NCT03322813
ExAblate Model 4000 Type 2	BBB	None	Active	The BBB is opened by ultrasound to facilitate drug absorption	NCT03551249
C225-ILs-dox	EFFR + Topoisomerase-II	I	Completed	Target specific targets to deliver drugs using liposome	NCT03603379
FUS BBB Disruption	BBB	None	Active	The BBB is opened by ultrasound to facilitate drug absorption	NCT03616860
NaviFUS System	BBB	None	Completed	The BBB is opened by ultrasound to facilitate drug absorption	NCT03626896
ExAblate Model 4000 Type 2	BBB	None	Unknown	The BBB is opened by ultrasound to facilitate drug absorption	NCT03712293
SonoCloud-9 + Carboplatin	BBB	I/II	Completed	The BBB is opened by ultrasound to facilitate drug absorption	NCT03744026
AGuIX®	None	II	Recruiting	Nanoparticle coated radiosensitizer used to improve the effect of RT	NCT03818386
ExAblate Model 4000 Type 2 + Carboplatin	BBB in rGBM	I/II	Active	The BBB is opened by ultrasound to facilitate drug absorption	NCT04440358
ExAblate Model 4000 Type 2 + Carboplatin	BBB in rGBM	I/II	Active	The BBB is opened by ultrasound to facilitate drug absorption	NCT04417088
NaviFUS System + Bevacizumab	BBB + VEGFR	None	Completed	The BBB is opened by ultrasound to facilitate drug absorption	NCT04446416
Sonication + Chemotherapy	BBB	I/II	Recruiting	The BBB is opened by ultrasound to facilitate drug absorption	NCT04528680
SonoCloud-9 (SC9) device + TMZ	BBB	II	Recruiting	The BBB is opened by ultrasound to facilitate drug absorption	NCT04614493
ExAblate Model 4000 Type 2	BBB	None	Suspended	The BBB is opened by ultrasound to facilitate drug absorption	NCT04667715
Magnetic Resonance guided Focused ultrasound	BBB	None	Recruiting	The BBB is opened by ultrasound to facilitate drug absorption	NCT04998864
NaviFUS System	BBB	None	Recruiting	The BBB is opened by ultrasound to facilitate drug absorption	NCT04988750

*CED* Convection enhanced delivery; *RT* Radiotherapy; *BBB* Blood brain barrier; *GBM* Glioblastoma; *rGBM* recurrent GBM; *FUS* Focused Ultrasound

Zinc ion carriers, known for their tissue specificity, have found extensive applications in this field. They are employed to modify engineering carriers with CpG oligonucleotide nanoparticles (CpG NPs) and AMD-Zn (Zn(II)2-AMD3100), creating an injectable hydrogel system (imGEL) that, among them, the tissue-specific affinity of zinc nanoparticles and the unique tissue diffusion and resident properties of hydrogels can increase the drug efficacy [700]. When delivered into the surgical cavity, it effectively inhibits persistent GAMs activation and stimulates CTLs. The results indicate that imGEL can modulate the TIME, suppress the recurrence of GBM, and provide precious time for follow-up clinical adjuvant therapy [701]. Hydrogels have also recently been extensively used due to their tissue-specific dispersion properties. Leveraging their diffusion characteristics, Chen et al. [627] combined a special hydrogel composite structure with GSC-specific CAR-MΦ to be injected into the tumor cavity following GBM resection in mice. This approach conferred powerful tumor-immune cytotoxicity in the surgical cavity, inhibiting GBM recurrence. Moreover, direct intratumoral administration is an emerging and highly effective approach in current cancer treatments, and ultrasound (US) possesses strong tissue-penetrating capabilities and has widespread clinical applications. So, sonodynamic therapy (SDT) is a novel approach that utilizes the principles of ultrasound to activate photosensitizers previously injected into tumor tissue, generating ROS and cavitation bubbles, thus eradicating GBM cells [702]. Several GBM combination therapy approaches based on SDT have been studied, like SDT-thermotherapy, SDT-autophagy inhibition, photodynamic therapy (PDT) with SDT, and SDT-chemotherapy. The above combinatorial methods synergize tumor ablation, significantly strengthening the effectiveness of GBM treatment [703]. Another adjuvant strategy based on CED can facilitate the improved delivery of drugs to the interior of GBM [704–706].

However, the applications of medicine and engineering go beyond that. They can be combined with other treatment approaches, such as OVs, engineered using engineering techniques to enhance their tissue specificity for tumor tissue. Moreover, bacteria-mediated tumor therapy can stimulate the immune system and carry various drugs with genetic engineering [707, 708]. Zhu et al. [709] used C-novyi-spores with melittin-RADA32 nanofiber hybrid peptide. It armed them with metformin, inducing the infiltration of CD8^+^ T cells, regulating immune-active factors secretion, and promoting the polarization of M1 macrophages, thus reactivating anti-tumor immunity in the GBM microenvironment. The integration of medicine and engineering can also be combined with therapies that target tumor metabolism. Both glioma cells and TAMs overexpress α7 nicotinic acetylcholine receptors (nAChRs) [710]. A lipid complex, CDX-LIPO, has been developed to target these receptors. It can co-target tumor cells, tumor vasculatures, and TAMs to restrain aerobic glycolysis through the mTOR pathway, thereby inducing tumor autophagy, suppressing M2 macrophages, and MDSCs while activating the function of CTL, M1 macrophages, and NK cells in GBM [711]. Applying engineering techniques can also improve the effectiveness of ICIs [712].

The integration of medical and engineering technologies has recently become a prominent strategy in cancer therapy. Various treatments, including immunotherapy, cell therapy, and metabolic therapy, are being modified using engineering technologies to achieve better targeting and improved tumor specificity. Furthermore, engineering modifications can enhance the efficacy of existing treatment modalities, ultimately strengthening their tumor-killing effects. This fusion of medicine and engineering represents a powerful tool in cancer treatment, providing innovative strategies to combat the complexity of cancer and improve patient outcomes.

### Tumor treating fields therapy in glioblastoma

Tumor treating fields (TTFs) represents a physical therapy approach in cancer treatment that involves applying low intensity, intermediate frequency, alternating electric fields (1–3 V/cm and 100 kHz to 300 kHz). This disrupts the processes of the mitotic spindle in rapidly dividing tumor cells, leading to chromosome missegregation, incomplete cytoplasmic separation, mitotic catastrophe, and p53-dependent and independent apoptosis [713]. TTFs have shown efficacy in extending patients with GBM prognosis, leading to FDA approval for treating GBM and rGBM after surgery and RT with adjuvant TMZ (combined with TMZ to extend median PFS to 6 months) [714–717]. TTFs have been observed to cause cell cycle arrest at the G2/S phase or disrupt G1/synthesis, along with enhancing ROS production to augment radiation-induced apoptosis [718–720]. Additionally, TTFs can delay DNA damage repair and enhance radiation-mediated DNA damage. The combination of TTFs with radiation treatment has been shown to promote caspase-3 and poly ADP-ribose polymerase (PARP) cleavage, contributing to a more effective killing of GBM cells [721]. Moreover, TTFs activate autophagy by inducing miR-29b, which inhibits the Akt2/mTOR/p70S6K/4EBP1 axis signaling, thereby inhibiting GBM progression in vitro [722]. TTFs have demonstrated multifaceted effects in GBM treatment. TTFs not only impact the cell cycle and apoptosis but also exhibit potential in modulating various signaling pathways associated with GBM proliferation and progression. For instance, TTFs have been found to reduce eukaryotic translation initiation factor 4A3 (EIF4A3)-mediated circMMD biosynthesis, which is elevated in GBM. The circMMD expressed highly is related to worse outcomes in GBM cases. By inhibiting Wnt/β-catenin pathway activation, TTFs contribute to the suppression of GBM proliferation [723]. Moreover, TTFs have been shown to induce anti-tumor immunity, potentially enhancing immunotherapy. TTFs promote the infiltration of tumor-infiltrating leukocytes in the TME. This results in increased PD-L1 expression in macrophages and DCs, as well as elevated release of IFN-γ by CTLs [724]. Combining TTFs and anti-PD-1/PD-L1 significantly reduces tumor volume, enhances anti-tumor immunity, and achieves a more potent anti-tumor effect. It's noteworthy that TTFs do not seem to adversely affect crucial functions of T cells involved in anti-tumor immunity. The secretion of IFN-γ, cytotoxic degranulation, and antigen-directed cytotoxic function in T cells exposed to TTFs remain unaffected. Although TTFs inhibit the T-cell activity in proliferation, the viability of non-proliferative T cells is not compromised [725, 726]. Interestingly, TTFs have been related to a significant upregulation in tumor antigen-specific infiltration of T cells in patients who received TTFs combined with standard chemoradiotherapy in GBM, with no apparent alteration in their proliferative capacity [725].

The recent study highlights the potential of TTFs in triggering immunogenic responses in GBM. TTFs-induced mitotic catastrophe leads to the local disruption of the nuclear envelope, resulting in the release of micronucleus within the cell. This, in turn, activates DNA sensing pathways such as cGAS/STING and is absent in melanoma 2 (AIM2), eliciting various inflammatory mediators, such as IL-6, CXLC10, IL-8, type 1 interferon, IL-1, and type 1 interferon-responsive genes production [727]. In addition to the in vitro findings, TTFs have been shown to stimulate STING/AIM2-mediated anti-tumor immunity in mice with GBM. This stimulation promotes T cell activation in the microenvironment and the generation of durable memory T cells. As a result, mice treated with TTFs were protected from re-challenge by the same GBM cell line [728]. These findings suggest that TTFs may have an immunomodulatory effect by promoting anti-tumor immunity. This highlights the potential of TTFs as a therapeutic strategy not only for directly targeting GBM but also for mounting an effective anti-tumor reaction. The implications of this research extend beyond GBM, hinting at the possibility of using TTFs as cancer immunotherapy for other solid tumors.

In the contemporary landscape, therapies for GBM have transitioned into an epoch characterized by comprehensive interventions. The profound intratumoral heterogeneity inherent to GBM renders singular therapeutic modalities susceptible to heightened drug resistance and recurrent manifestations. Moreover, propelled by advancements in scRNA-seq, the discernment of various drug-sensitive and drug-resistant cellular clusters within GBM has become feasible. Consequently, the amalgamation of diverse treatment modalities emerges as a strategic imperative for surmounting the constraints precipitated by the heterogeneity intrinsic to singular treatment modalities. This strategic amalgamation is oriented towards realizing a comprehensive therapeutic impact, delineating a departure from unilaterally oriented approaches.

### Potential prospects for targeting MDSC in glioblastoma

**Fig. 7:**
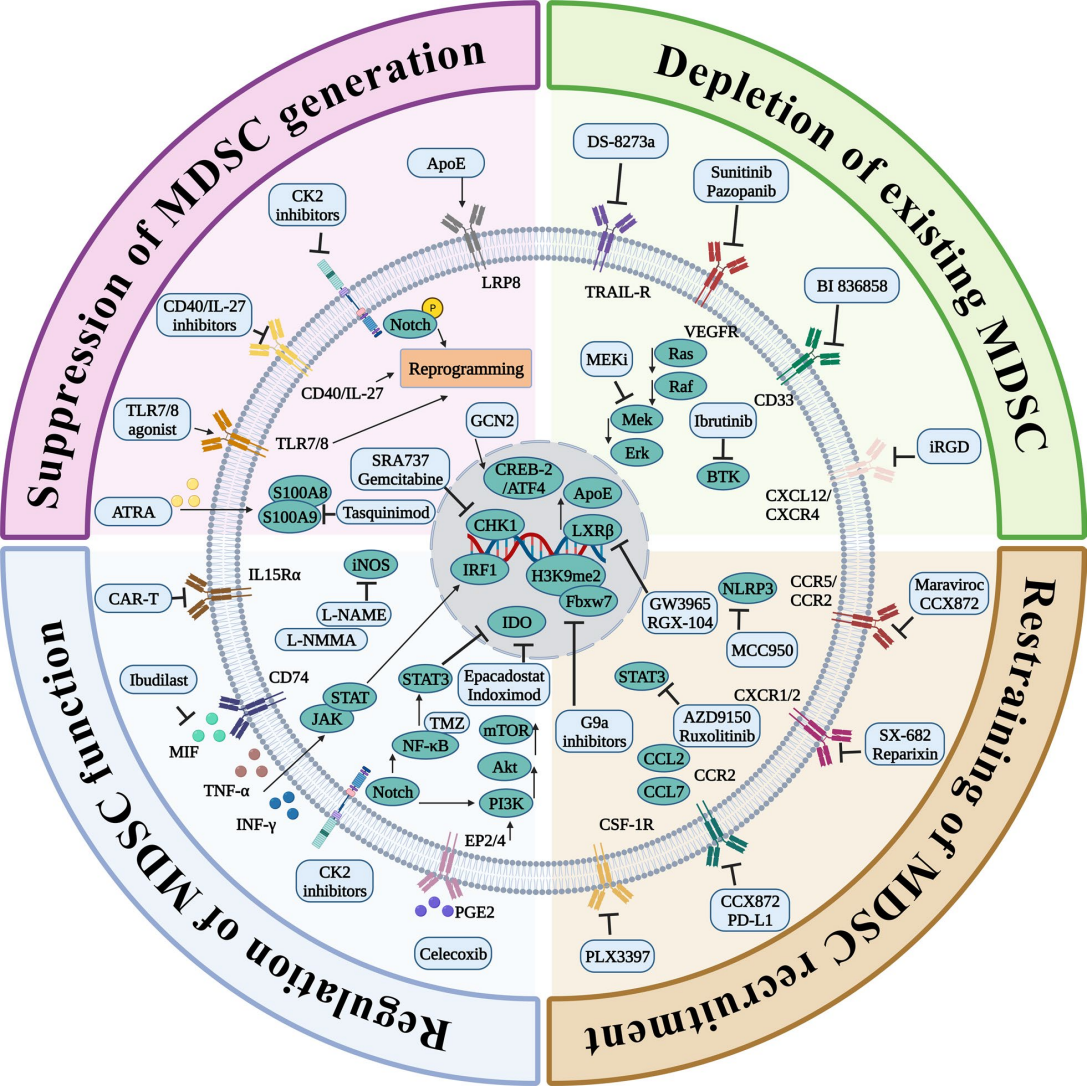
Therapeutic strategies targeting MDSC. Current therapeutic strategies targeting MDSCs can include four steps: suppressing the generation or expansion of MDSCs, depleting the existing MDSCs, restraining the recruitment of MDSCs, and regulating the immunosuppressive function of MDSCs. *Akt* Protein kinase B; *ApoE* Apolipoprotein E; *ATRA* All-trans-retinoicacid; *BTK* Bruton’s tyrosine kinase; *C/EBPβ* CCAAT/enhancer binding protein β; *CAR-T* Chimeric antigen receptor T-Cell immunotherapy; *CHK1* Checkpoint kinase 1; *CK2* Casein kinase 2; *CSF-1* Macrophage colony-stimulating factor-1; *Erk* Extracellular regulated protein kinases; *Fbxw7* F-box and WD-40 domain protein 7; *GCN2* General control nonderepressible 2 kinase; *IDO* Indoleamine2,3-dioxygenase1; *IFN-γ* Interferon γ; *IL* Interleukin; *iNOS* inducible nitric oxide synthase; *IRF* Interferon regulatory factor; *iRGD* internalizing RGD; *JAK* Janus Kinase; *LRP8* Low-density lipoprotein receptor-related protein 8; *LXRβ* Liver X receptor β; *MDSC* Myeloid-derived suppressor cells; *MIF* Macrophage migration inhibitory factor; *NLRP3* NOD-like receptor thermal protein domain associated protein 3; *PGE2* Prostaglandin E2; *PI3K* Phosphoinositide-3 kinase; *STAT* Signal transduction and transcription factor; *TLR2* Toll-like receptor 2; *TMZ* Temozolomide; *TNF* Tumor necrosis factor; *TRAIL-R* Tumor necrosis factor-related apoptosis-inducing ligand receptor; *VEGF* Vascular endothelial growth factor

The heightened infiltration of MDSCs within the TME intricately correlates with tumor invasiveness, compromised efficacy of immunotherapy, and a more unfavorable prognosis. Elevated MDSC levels are discernible in the peripheral circulation of GBM patients, a phenomenon mediated by arginase activity and G-CSF, with ensuing reversible dysfunction observed in T cells [19]. Consequently, targeting MDSCs stands out as a promising therapeutic avenue in the GBM treatment landscape. Four primary therapeutic strategies have evolved for MDSC targeting: the inhibition of MDSC generation, depletion of MDSC populations, curbing MDSC recruitment to the TME, and interference with the immunosuppressive functionality of MDSC. Refer to Table 10 and Fig. 7 for a comprehensive summary of available MDSC-targeting strategies in tumors.

**Table 10:** Current ongoing clinical trials based on MDSCs therapy

Agents	Targets	Phase	Status	Mechanism	Trial ID
	PI3Kγ	I	Active, not recruiting	Regulate the function of MDSCs	NCT02637531
	CXCR1/2	I	Recruiting	Reduce MDSCs recruitment	NCT04477343
Vorinostat + Hydroxy-chloroquine	HDAC	I	Completed	Regulate the function of MDSCs	NCT01023737
Quisinostat + Chemotherapy	HDAC	I	Completed	Regulate the function of MDSCs	NCT02728492
Ibrutinib + Nivolumab	Tyrosine kinase	I	Unknown	Reduce MDSCs recruitment	NCT03525925
AZD9150 + Durvalumab	STAT3	I	Active, not recruiting	Inhibition of differentiation of MDSCs	NCT03421353
	STAT3	I	Completed	Inhibition of differentiation of MDSCs	NCT01904123
Capecitabine + Bevacizumab	Chemotherapy	I	Active, not recruiting	Reduce MDSCs recruitment	NCT02669173
	LXRβ	I	Recruiting	Reduce MDSCs recruitment	NCT02922764
Panobinostat + Ipilimumab	HDAC	I	Completed	Regulate the function of MDSCs	NCT02032810
	Casein Kinase	I	Active, not recruiting	Reduce MDSCs recruitment	NCT03897036
	STAT3	I	Active, not recruiting	Inhibition of differentiation of MDSCs	NCT03195699
DS-8273a	TRAIL-R2	I	Completed	Depletion of MDSCs	NCT02076451
OPB-31121	STAT3	I	Completed	Inhibition of differentiation of MDSCs	NCT00955812
	HDAC	I	Completed	Regulate the function of MDSCs	NCT00697879
Maraviroc + Nivolumab + Ipilimumab	CCR5	I	Unknown	Reduce MDSCs recruitment	NCT04721301
Slidenafil + Regorafenib	PDE5	I	Completed	Regulate the function of MDSCs	NCT02466802
OPB-51602	STAT3	I	Completed	Inhibition of differentiation of MDSCs	NCT01423903
Omaveloxolone + Ipili-mumab + Nivolumab	NF-κB	I/II	Completed	Regulate the function of MDSCs	NCT02259231
Pazopanib + Topotecan	Tyrosine kinase	I/II	Completed	Reduce MDSCs recruitment	NCT02303028
	STAT3	I/II	Not yet recruiting	Inhibition of differentiation of MDSCs	NCT04733521
Ibrutinib	Tyrosine kinase	I/II	Completed	Reduce MDSCs recruitment	NCT02321540
Chidamide + Toripalimab	HDAC	I/II	Recruiting	Regulate the function of MDSCs	NCT04651127
CX-4945 + Chemotherapy	Casein Kinase	I/II	Completed	Reduce MDSCs recruitment	NCT02128282
SX-682 + Nivolumab	CXCR1/2	I/II	Recruiting	Reduce MDSCs recruitment	NCT04599140
ATRA + Pembrolizumab	ERK1/2	I/II	Active, not recruiting	Inhibition of differentiation of MDSCs	NCT03200847
Celecoxib + Radiation Therapy	COX2	I/II	Completed	Regulate the function of MDSCs	NCT00046839
	CXCR2	I/II	Active, not recruiting	Reduce MDSCs recruitment	NCT03177187
ATRA + Anastrozole	Vitamins	II	Recruiting	Inhibition of differentiation of MDSCs	NCT04113863
ATRA + 5-Azacitidine + Lupron	Vitamins	II	Completed	Inhibition of differentiation of MDSCs	NCT03572387
Pazopanib	Tyrosine kinase	II	Completed	Reduce MDSCs recruitment	NCT01956669
Vesanoid + Ipilimumab	Vitamins	II	Active, not recruiting	Reduce MDSCs recruitment	NCT02403778
Pazopanib + Durvalumab	Tyrosine kinase	II	Completed	Reduce MDSCs recruitment	NCT03798106
ATRA + Chemotherapy	Vitamins	II	Not yet recruiting	Inhibition of differentiation of MDSCs	NCT04241276
Entinostat + Azacitidine	HDAC	II	Completed	Regulate the function of MDSCs	NCT01105377
Vicriviroc + Pembrolizumab	CCR5	II	Completed	Reduce MDSCs recruitment	NCT03631407
	HDAC	II	Completed	Regulate the function of MDSCs	NCT01075308
Leronlimab	CCR5	II	Active, not recruiting	Reduce MDSCs recruitment	NCT04504942
Ruxolitinib	JAK/STAT3	II	Recruiting	Inhibition of differentiation of MDSCs	NCT03153982
Tadalafil	PDE5	II	Completed	Regulate the function of MDSCs	NCT01697800
Celecoxib + Nivolumab	COX2	II	Recruiting	Regulate the function of MDSCs	NCT03026140
Celecoxib	COX2	III	Completed	Regulate the function of MDSCs	NCT02429427
SX-682 + Pembrolizumab	CXCR1/2	III/IV	Recruiting	Reduce MDSCs recruitment	NCT03161431

*COX2* Cyclooxygenase 2; *ERK* Extracellular regulated protein kinases; *HDAC* Histone deacetylase; *JAK* Janus Kinase; *LXRβ* Liver X receptor β; *MDSCs* Myeloid-derived suppressor cells; *PDE5* Phosphodiesterase 5; *PI3K* Phosphoinositide-3 kinase; *STAT* Signal transduction and transcription factor; *TRAIL-R* Tumor necrosis factor-related apoptosis-inducing ligand receptor

#### Suppression of MDSC generation

In recent years, ICIs have emerged as pivotal components of cancer therapy. Sen et al. demonstrated that combining oral checkpoint kinase 1 (CHK1) inhibitor SRA737 with gemcitabine significantly augmented the amount of CD8^+^ T cells, DCs, and M1 macrophages in small cell Lung cancer (SCLC) models [729]. This therapy concomitantly induced a marked reduction in M2 macrophages and MDSCs. The resultant attenuation of the immunosuppressive microenvironment holds promise for strengthening anti-tumor results combined with anti-PD-L1/anti-PD-1 [730]. Targeting CD33, a standard marker for human MDSCs, is applied to treat acute myeloid leukemia [731]. Recent studies have revealed that metformin, belonging to a class of drugs capable of activating the AMPK pathway and inhibiting the mTOR pathway, can diminish the levels of S100A8/A9 and ARG1. This reduction, coupled with an upregulation in CD8^+^ T cells, collectively inhibits the population of PMN-MDSCs when combined with ICIs [732]. Additionally, all-trans retinoic acid (ATRA) can impede retinoic acid signaling, prompting the conversion of MDSCs into MONs and DCs [356]. This process involves the activation of extracellular regulated protein kinases 1/2 (ERK1/2) and generating glutathione, which has anti-angiogenic effects in breast cancer [733]. ATRA-based therapies are presently undergoing evaluation in melanoma, renal cell carcinoma (RCC), and lung cancer, showcasing significant reductions in MDSC and improved prognoses. Casein kinase 2 (CK2) inhibitors represent an additional strategy for impeding MDSC differentiation, particularly targeting PMN-MDSCs differentiation by regulating the Notch phosphorylation pathway [734–736]. When combined with anti-CTLA-4, CK2 inhibitors can inhibit bone marrow cell differentiation and diminish PMN-MDSC generation [734]. While MDSCs are traditionally considered to originate from the bone marrow, recent studies have illuminated the spleen as an additional reservoir of MDSCs [737]. In lung adenocarcinoma, researchers have identified substantial migration of MDSC precursors from the spleen to the TME. These cells promote CCR2 signaling, which is crucial for recruiting spleen-derived MDSCs in vivo [738, 739]. Notably, splenectomy, either before or after tumor development, significantly attenuates MDSC responsiveness and retards tumor progression. Liver X Receptors (LXRs) activate genes about glucose metabolism, cholesterol, and FA regulation transcription [740]. Agonists of LXR, such as GW3965 and RGX-104, currently undergoing Phase I clinical trials, have demonstrated potent anti-tumor effects in immune-competent mice, inhibiting tumorigenesis, including GBM [741, 742]. These agonists induce the up-regulation of apolipoprotein E (ApoE), a transcriptional target of LXR, which acts on the LRP8 receptor on MDSCs. This action reduces the abundance of tumor-infiltrating and systemic MDSCs, concurrently increasing CD8^+^ and CD4^+^ T cells infiltrating into the microenvironment. This modulation aims to reverse tumor immune evasion and promote anti-tumor immunity [743].

#### Depletion of MDSC

MDSCs, highly heterogeneous cells originating from BM, impose limitations on the efficacy of immunotherapy in tumors. The elimination of MDSCs within the TIME has demonstrated a substantial enhancement in the anti-tumor effects of immunotherapy, leading to a noteworthy extension in the mice’s prognosis in tumors. MDSCs in both mice and tumor-afflicted patients exhibit a significantly heightened ERS response compared to their counterparts in normal mice and healthy individuals. Multiple factors can induce ERS in MDSCs, among which an elevation in ROS within MDSCs is noteworthy [744, 745]. Induction of DR5 expression in mouse MDSCs through ERS inducers has been observed. Targeting DR5 effectively eliminates MDSCs via caspase-8-mediated apoptosis, facilitating the expansion and augmenting the cytotoxic activity of CD8^+^ T cells. This, in turn, significantly amplifies the anti-tumor efficacy of anti-CTLA-4, particularly in weakly immunogenic tumors [403]. Resiquimod, a TLR7/8 agonist, exerts anti-viral and anti-tumor immunomodulatory effects by stimulating various cytokines secretion [746, 747]. In a breast cancer mouse model, resiquimod induces F4/80^+^ macrophages and CD11c^+^/I-A^+^ DCs, differentiating from MDSCs. These differentiated cells exhibit heightened proliferation-inducing activity on antigen-primed T cells and robustly stimulate the proliferation of CD4^+^ and CD8^+^ T cells, reinforcing anti-cancer immunity [748]. Furthermore, the loss of the serine-threonine kinase general control nonderepressible 2 (GCN2), a key driver in the polarization of MDSCs, leads to the transition of immunosuppressive MDSCs to an antitumor-responsive phenotype in the TME. This transition is achieved by promoting the transcription of cyclic-AMP response binding protein 2/ATF4 (CREB2/ATF4), strengthening proinflammatory responses, and enhancing IFN-γ secreted by CD8^+^ T cells [749]. Notably, patients with pre-existing or newly diagnosed systemic autoimmune conditions have been reported to exhibit a significantly increased likelihood of developing tumors, particularly melanoma [750]. Excessive immunosuppressive therapy in cancer patients can induce elevated IFN-γ, potentially triggering de novo autoinflammation and exacerbating pre-existing autoimmune conditions [751]. The expansion of MDSCs derived from systemic lupus erythematosus (SLE) in the context of melanoma has been implicated in driving systemic macrophage polarization. Notably, SLE-derived MDSCs interact with autoimmune macrophages to suppress CD40 expression and IL-27 production on the cell surface. This inhibition of CD40/IL-27 signaling in tumors is associated with increased TAM infiltration and resistance to ICB. In GBM, the selective depletion of MDSCs using low doses of 5-Fluorouracil (5-FU) has demonstrated increased activated-T-cell amount and extended mice prognosis [403]. Oral administration of the 5-FU prodrug capecitabine in rGBM patients activated anti-tumor immunity, including CD8^+^ T cells and NK cells. This treatment also led to reduced circulating MDSCs, which is related to a more favorable prognosis [752]. Conversely, dexamethasone, used to treat peritumoral edema in GBM patients, promotes abnormal myeloid lineage cell proliferation in the bone marrow. This increased proportion of MDSCs contributes to the immunosuppressive microenvironment in GBM. This effect is associated with the immunosuppressive response to corticosteroids and is considered reversible [752]. Consequently, the management of peritumoral edema during the perioperative period in GBM warrants reevaluation.

GBM necessitates a comprehensive treatment approach, emphasizing maximal surgical resection followed by a combination of RT, chemotherapy, and immunotherapy or targeted therapy. Maximal surgical resection not only aims at reducing the tumor burden but has also been observed to decrease MDSCs: tumor debulking significantly diminishes MDSCs. It facilitates CD4^+^ and CD8^+^ T-cell recruitment. This synergistic approach, especially when combined with immunotherapy, strengthens anti-tumor efficacy [753]. Elevated TIGIT expression on TIL has been associated with reduced CTL cytokine production and poorer survival outcomes [754]. In a murine GBM model, TIGIT blocking stimulated anti-tumor CTL responses and concurrently reduced the number of immunosuppressive PMN-MDSCs [235]. Within the GBM microenvironment, pro-angiogenic cytokines such as VEGF and Ang-2 are highly expressed. These cytokines drive tumor angiogenesis and vascular permeability while negatively regulating T cells and the innate immune response [755, 756]. Targeted VEGF therapy has shown promise in alleviating immunosuppression, allowing T cells to enter the TME and function effectively. Combined with ICIs, anti-VEGF/Ang-2 treatment has demonstrated enhanced infiltration of CD8^+^ T cells, reduced immunosuppressive MDSCs, and diminished FOXP3^+^ Tregs, thereby improving the efficiency of immunotherapy [515]. The TIME poses a significant obstacle to CAR-T therapy in GBM. Notably, GBM patient TME cells, including MDSCs, exhibit significantly elevated levels of IL15Rα [757]. IL15Rα-targeted CAR-T (CAR-IL15-T) effectively depletes MDSCs within the TME, inhibits the secretion of immunosuppressive molecules by MDSCs, and extends the survival of GBM mouse models. Moreover, combining B7-H3-targeted CAR-T and OVs with chemokine CXCL11 (oAd-CXCL11) achieves superior anti-tumor effects in GBM. oAd-CXCL11 contributes to TIME reprogramming by facilitating M1 macrophage, CD8^+^ T cell, and NK cell infiltration while concurrently depleting MDSCs, Tregs, and M2 macrophages [758].

#### Restraining of MDSC recruitment to the TME

Two distinct sets of signals govern the recruitment of MDSCs. Firstly, there is the induction of emergency myelopoiesis and the modulation of myeloid cell differentiation, primarily mediated through G-CSF and GM-CSF. The second signal involves the activation of MDSCs, predominantly mediated by pro-inflammatory cytokines, like IL-6, IL-1β, IFN-γ, and IL-4 [759, 760]. Research has demonstrated that mitogen-activated protein kinase (MEK) inhibitors can reduce GM-CSF and IL-6 production, thereby restraining the recruitment of MDSCs while concurrently promoting CD8^+^ T-cell recruitment. This microenvironment reprogramming aims to restore the sensitivity of Kirsten rat sarcoma viral oncogene (KRAS)-mutant tumors to PARP inhibitors and anti-PD-1/PD-L1 therapy [761]. The synergistic combination with MEK inhibitor, PARP inhibitor, and anti-PD-1/PD-L1 therapy has shown potential for achieving a more sustained anti-tumor response [761, 762]. Inhibition of the CXCL12/CXCR4 signaling pathway has been identified as another strategy to modulate MDSC recruitment and enhance anti-tumor responses. Targeting this pathway not only inhibits tumor cell proliferation but also restrains the recruitment of CXCR4^+^ M-MDSCs to the TME. Additionally, it contributes to restoring BBB integrity and induces immunogenic cell death (ICD), thereby sensitizing tumors to complementary therapies such as RT and fostering an anti-GBM immune response [763]. Within specific cancer types like oral and lung cancers, PMN-MDSCs constitute the predominant myeloid cell subpopulation. SX-682, an oral small-molecule CXCR1/CXCR2 inhibitor currently undergoing clinical evaluation, demonstrates significant efficacy in inhibiting the recruitment of CXCR1^+^ PMN-MDSCs. This inhibition is accompanied by an enhancement in the accumulation of endogenous or adoptively transferred T cells, thus facilitating the effectiveness of T cell-based immunotherapies, including ICBs and adoptive T cell transfer. Importantly, this occurs without altering the expression of CXCR2 ligands and the trafficking of CXCR1^+^ macrophages [764, 765]. In patients with head and neck squamous cell carcinoma (HNSCC), CD14^+^ M-MDSCs and CXCR1/2^+^/CD15^+^ PMN-MDSCs evident infiltration is observed both in the circulation and at tumor sites [765]. Notably, MDSCs within tumors exhibit a more pronounced immunosuppressive effect than those present in the circulation. The small-molecule inhibitor SX-682 has demonstrated efficacy in mitigating MDSCs accumulating within tumors through blocking CXCR1/2, thereby inhibiting PMN-MDSCs recruiting [766–768]. This intervention enhances the anti-tumor efficiency in NK cells. Importantly, SX-682 does not directly alter the proliferation, survival, or sensitivity of tumor cells to NK cells, and it does not affect the immunosuppressive function of PMN-MDSC. TAMs play multifaceted roles in tumor development, making them an attractive target for therapeutic intervention [769]. However, targeting TAMs with CSF-1R inhibitors has shown limited antitumor efficacy. Tumor cells producing CSF-1 can down-regulate granulocyte-specific chemokine in CAFs through HDAC2-mediated pathways, inhibiting myeloid cells recruited into tumor. Paradoxically, blocking CSF-1R can result in CAFs secreting numerous cytokines, recruiting PMN-MDSCs into the tumor. The use of CXCR2 inhibitors can counteract the adverse effects of CSF-1R blockade. As most chemokines bind to CXCR2, up-regulation of CXCR2 induced by CSF-1R blockade can be mitigated by CXCR2 inhibitors, preventing the chemokines secreted by CAFs from functioning. This inhibition of CXCR2 enhances the antitumor effect of CSF-1R inhibitors by restraining the recruitment of PMN-MDSCs. In the breast cancer models, the PARP inhibitor inhibits the recruitment of MDSCs mediated by CXCR4. This inhibition is achieved by reducing stromal cell-derived factor 1 alpha (SDF1α) released by CAFs, thereby augmenting the anti-tumor effect of EGFRvIII targeted CAR-T therapy [770].

RT has been a longstanding and integral component of GBM treatment, contributing to enhanced local control rates and extended survival. Despite its importance, RT can induce local inflammatory responses, including generating complement C5a, a classical inducer of MDSCs [771–773]. Consequently, there is an induction of MDSC recruitment. Resistance to tumor RT arises from mechanisms such as STING signal activated through RT. This activation induces IFN-β secretion within tumor cells, inducing the secretion of chemokines like CCL12, CCL2, and CCL7. These chemokines attract CCR2^+^ M-MDSCs to the TME [774–776]. However, it's noteworthy that RT, particularly at high doses, can also decrease MDSC levels. Ablative hypofractionated radiotherapy (AHFRT), instead of conventionally fractionated radiotherapy (CFRT), has been observed to downregulate the amount and immunosuppressive function in MDSCs. This effect is attributed to reduced intratumor hypoxia and VEGF [777]. Combining a single dose of AHFRT with anti-PD-1/PD-L1 treatment activates CD8^+^ T cells and reduces MDSC levels. This strategy induces the generation in T cells and DCs, further leading to the elimination of MDSCs in GBM-bearing mice [749]. In the GBM microenvironment, chemokines CCL2 and CCL7, secreted by both tumor and non-tumor cells, redundantly contribute to the migration of CCR2^+^/CX3CR1^+^ M-MDSCs into the TME. This population of MDSCs can directly impede CD4^+^ and CD8^+^ T-cell proliferation and activation, exacerbating the TIME in GBM [778]. Furthermore, CCL2 expression has been verified to negatively correlate with the survival time of GBM patients, with patients with low expression of CCL2 surviving longer than those with high expression of CCL2 [421]. Disruption of the CCL2/CCR2 axis inhibited intratumoral MDSCs’ recruitment and led to the related accumulation of these cells in the BM but had no effect on the intratumoral T cell population [401]. Additionally, studies have shown that gram-negative bacteria/LPS can induce the production of TLR4-dependent CXCL1 in hepatocytes, which induces CXCR2^+^ PMN-MDSCs infiltrating in TME, thereby regulating the formation of an immunosuppressive microenvironment in hepatocytes and promoting liver tumor growth [736]. Neomycin treatment can block CXCL1 and PMN-MDSC accumulating and inhibit tumor growth. Sunitinib, a tyrosine kinase inhibitor, is the oral compound permitted by the FDA for first-line treatment of various cancers [779]. In the mouse glioma model, CD4^+^ T cells increased, and MDSCs recruitment decreased after sunitinib treatment, and the reduced amount of MDSCs was consistent with the increased CD4^+^ T cell quantity and higher proliferation ability, resulting in tumor reduction and significantly prolonged mouse survival [780]. The CXCR4/CXCL12 signaling pathway is crucial in the homing and migration of immune cells [781]. CXCR4 is commonly expressed in hematopoietic cells like MDSCs, T cells, microglia, and B cells, overexpressing in various tumors, including GBM. It contributes to tumor treatment resistance by recruiting immunosuppressive bone marrow cells and promoting abnormal tumor angiogenesis [782]. Anti-CXCR4 therapy can reduce the amount of immunosuppressive tumor-infiltrating leukocytes, like MDSCs and intracranial microglial cells. Targeting MDSC with anti-CXCR4 promotes anti-PD-1 anti-tumor immune responses and improves GBM mouse survival through modulation of the myeloid and T cell TME and the underlying tumor bed vasculature [783–785]. Therefore, targeting MDSC to reprogram the immunosuppressive microenvironment is promising to enhance the efficacy of other anti-tumor immunotherapies in GBM.

#### Regulation of MDSC’s immunosuppressive function

The success of immune checkpoint therapy has instilled optimism regarding the potential cure for cancer. However, a substantial proportion of patients remain unresponsive, and many experience relapse due to immune escape. Among the critical elements contributing to resistance to ICIs, the presence of MDSCs within cancers stands out. MDSCs drive T-cell exhaustion and dysfunction, ultimately leading to immunosuppression. Therefore, the strategic targeting of MDSCs to convert GBM from a "cold" tumor, refractory to immune response, to a "hot" tumor that responds favorably to immunotherapy holds significant therapeutic promise (Fig. 7).

In the pursuit of developing targeted therapies against MDSCs to counteract immunosuppression, MIF has emerged as a notable candidate. MIF exhibits expression in different tumors, including GBM, lung cancer, and breast cancer. Several immune cells, like neutrophils, T cells, MONs, and macrophages, can produce MIF [786]. Particularly noteworthy is the induction of MIF expression by glucocorticoids, commonly used for edema in GBM patients. The levels of MIF increase with glioma grade and upregulation of MIF is related to worse outcomes [787]. Investigations have revealed that M-MDSCs express elevated CD74, a MIF cognate receptor, and are expressed within the TME of GBM [112]. Ibudilast, a brain-permeable inhibitor, can effectively restrain the MIF/CD74 signaling pathway, diminish the immunosuppressive functions in MDSCs, and enhance the activity of CD8^+^ T cells in the microenvironment. Furthermore, clinically approved MIF inhibitors have been developed, showcasing the potential for repurposing in treating GBM [788–790]. The IRF8 has been identified as a crucial player in normal bone marrow formation and the secretion of certain pro-inflammatory type 1 cytokines, like IL-12p40 and CCL5 [791]. Notably, a robust negative correlation exists between the expression of IRF8 and the presence of MDSCs in tumors. Increased expression of IRF8 has been shown to mitigate the pro-tumorigenic capabilities of cancer-induced MDSCs [792]. As MDSCs emerge in response to cancer-derived factors [759], several transcription factors are implicated in STAT3 or STAT5 signaling pathways, with the activation of STAT3 or STAT5 playing various roles in MDSC biology [418, 793–797]. Research indicates that MDSC-inducing factors like GM-CSF and G-CSF in TME promote IRF8 downregulating through STAT3 and STAT5-dependent signals. The reduction in IRF8 is correlated with an increase in MDSC frequency [798, 799]. Downregulation of IRF8 in MDSCs can also influence the expression of Bax and Bcl-xL, suppressing FAS-mediated spontaneous apoptosis and facilitating evasion from elimination by CTLs [800]. Elevated levels of IRF8 have been demonstrated to alleviate the immunosuppressive characteristics of MDSCs, thereby enhancing the efficacy of immunotherapy. MDSCs, known as major producers of IL-6, exhibit significantly higher IL-6 production compared to tumor cells in tumor-bearing mice [797]. IL-6, generated by MDSCs, serves a dual role by safeguarding these cells from TNF-α-mediated necroptosis and sustaining their immunosuppressive functions within the TME. This is achieved through up-regulation of DNMT1 and DNMT3b via STAT3 activation in an autocrine pathway. Additionally, IL-6 can enhance the immunosuppressive abilities of MDSCs by increasing ARG1 activity and ROS production through STAT3 signaling [368]. The STAT3 plays a pivotal role in MDSC functions, and its inhibition has been shown to disrupt MDSC-mediated immunosuppression [801]. Blocking STAT3 induces apoptosis in MDSCs and reduces the expression of immunosuppressive factors [802, 803]. IDO is associated with tumor invasiveness and advanced metastasis [804]. IDO-positive cancer patients often exhibit high expression of inhibitory MDSCs, which inhibit T-cell activation and facilitate FOXP3^+^ Tregs’ differentiation and activation through the production of kynurenine [467, 805, 806]. Inhibiting IDO with a selective inhibitor has been shown to reverse the (TIME by reducing the infiltration of MDSCs and Tregs and eliminating their suppressive functions in vivo. Cysteine, crucial for mammalian protein synthesis and cell proliferation, is required by T cells for antigen presentation and activation [807]. MDSCs, lacking the neutral amino acid transporters, acquire cysteine from the environment without exporting it. This consumption of cysteine limits its availability in the extracellular environment, suppressing the T-cell activation and anti-tumor immunity [808–810]. Targeting amino acid metabolism to inhibit MDSC function and restore the antitumor effect of T cells represents a potential strategy [466, 811, 812]. Entinostat, an HDAC inhibitor, has been shown to reduce MDSC infiltration and its inhibitory functions through STAT3-mediated down-regulation of ARG1. When combined with ICIs, entinostat significantly alters innate immune cells' infiltration and activity, leading to a more effective adaptive immune reation [429, 803, 813].

## Conclusions

**Fig. 8:**
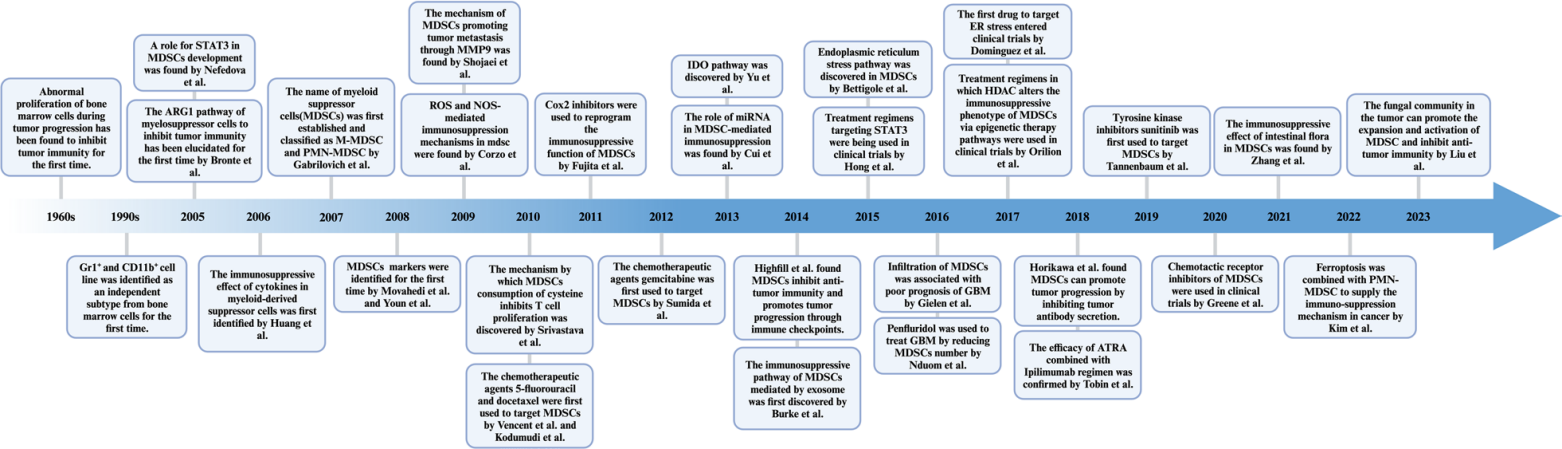
Timeline depicting the history of targeted MDSC anti-tumor therapy strategy. *ARG1* Arginase 1; *ATRA* All-trans-retinoic acid; *Cox2* Cyclooxygenase 2; *GBM* Glioblastoma; *HDAC* Histone deacetylase; *IDO* Indoleamine 2,3-dioxygenase; *M-MDSCs* Monocytic myeloid-derived suppressor cells; *MDSCs* Myeloid-derived suppressor cells; *miRNA* MicroRNA; *NOS* Nitric oxide synthase; *PMN-MDSCs* Polymorphonuclear myeloid-derived suppressor cells; *STAT* Signal transduction, and transcription factor

The intricate and highly heterogeneous TME is essential in the initiation and advancement in GBM. GBM is characterized by pronounced intratumor heterogeneity and a variable immunosuppressive milieu, contributing to drug resistance, frequent recurrence, and rapid disease progression. Among the significant contributors to the TME of GBM, MDSCs emerge as pivotal players, showcasing their essential role in shaping the immune landscape of aggressive brain tumors. The occurrence, recruitment, and dynamic functional alterations of MDSCs exhibit remarkable diversity across distinct stages of glioma development, orchestrated by various regulatory mechanisms. This diversity is further complicated by the profound influence of the heterogeneous microenvironment within gliomas on the function and differentiation of MDSCs. Figure 8 illustrates the timeline of key events in the establishment of targeting MDSCs as a novel therapeutic approach.

As indicated earlier, compelling evidence underscores the significance of the intricate interactions between tumor cells and stromal cells in developing GBM and resistance to immunotherapy. Cancer cells actively recruit and instruct stromal cells, including MDSCs and T cells, during their evolution. Conversely, infiltrating stromal cells are vital to enhance the aggressiveness of cancer cells, leading to resistance against immunotherapy. These observations highlight the potential of targeting the interaction in the tumor and the microenvironment as a promising therapeutic strategy for GBM. Recently, ICI has profoundly transformed the tumor treatment landscape, gaining FDA approval for its safety and feasibility in various malignancies. However, its efficacy in clinical trials for GBM remains under investigation. Presently, the standard treatment for GBM involves post-tumor resection RT combined with TMZ, constituting the primary therapeutic approach. It's important to note that both RT and TMZ have immunosuppressive effects. Additionally, the GBM microenvironment poses a challenging barrier to anti-tumor immune responses, emphasizing the need for a nuanced understanding of this complexity in developing immunotherapeutic strategies. Hence, there is an urgent imperative for combination therapies aimed at transforming these "cold" tumors into "hot," thereby augmenting existing immunotherapy approaches. MDSCs, by inhibiting host immune responses to tumors, play a pivotal role in immunotherapy resistance.

In Tables 4, 5, 6, 7, 8, a comprehensive summary revealed that a substantial portion of clinical studies across immune checkpoint therapy, targeted therapy, CAR-T, tumor vaccine therapy, OVs, ADCs, ITs, and integration of medicine and engineering technology encountered early-stage treatment failures and excessive complications, leading to premature trial termination. Upon systematic categorization of these clinical trials, it was observed that targeted therapy for GBM boasts the highest number of ongoing trials (69 in Active and recruiting), positioning it as the most actively pursued modality. Cancer-related vaccines, recognized as a burgeoning treatment avenue, also exhibit a noteworthy count of ongoing trials in the "Active" status. However, an assessment of the maturity of extant treatment methods, particularly those in phase II and more advanced, indicates that immune checkpoint therapy, tumor vaccine therapy, and targeted therapy lead the landscape. This underscores the relative maturity and safety of immunotherapy and targeted therapy within the contemporary spectrum of novel treatment approaches for GBM. While the field of tumor vaccines is steadily advancing, the anticipated progress in treatment strategies across these three domains is a promising prospect for the future. Despite the multitude of ongoing clinical trials, the impact on the prognosis of GBM remains limited, emphasizing the urgent need for innovative and effective treatment modalities for patients.

Consequently, combining alternative strategies that target MDSCs with active or passive immunotherapy holds the promise of synergistic effects. Most of the existing therapeutic strategies for MDSC are in the early stages of clinical trials. However, existing MDSC-targeting treatments face challenges due to the unclear phenotype, significant heterogeneity, and complex origin and functional networks of MDSCs [814]. To address these challenges, it is essential to employ high-throughput proteomics and genomics technologies to investigate the phenotype and characteristics of MDSCs in various tumor types. This will pave the way for precise methods to eliminate MDSCs. Moreover, the complexity of MDSC binding to tumor cells makes isolating MDSCs challenging, leading researchers to focus primarily on the overall MDSC population rather than tumor-infiltrating MDSCs. As different MDSC subtypes exhibit distinct regulatory mechanisms, identifying and understanding their unique functions is crucial for accurately targeting specific subtypes. Notably, MDSCs share similar phenotypes with normal bone marrow cells, posing a challenge for selective targeting. Therefore, targeting MDSCs in tumor patients must consider tumor site, stage, molecular type, and others. Various drugs have been demonstrated to inhibit the effects of MDSCs in tumors, with some receiving FDA approval, others undergoing clinical trials, and some being studied in preclinical models [815]. However, the intricate mechanisms involved in the generation, recruitment, activation, and immune suppression of MDSCs make it seemingly impossible to induce potent antitumor effects through a single approach. Consequently, combining MDSC-targeted therapy with other immunotherapies emerges as the preferred strategy.

## Data Availability

Not applicable.

## Abbreviations

5-FU: 5-Fluorouracil
5hmC: 5-Hydroxymethylcytosine
5mC: 5-Methylcytosine
AC-like: Astrocyte-like
ACLY: ATP-citrate lyase
ADC: Antibody–drug conjugate
ADCC: Antibody-dependent cellular cytotoxicity
ADCP: Antibody-dependent cellular phagocytosis
ADP: Adenosine diphosphate
ADV: Adenovirus
AHFRT: Ablative hypofractionated radiotherapy
AHR: Aryl hydrocarbon receptor
AIM2: Absent in melanoma 2
Akt: Protein kinase B
ALKBH5: AlkB homolog 5
AMPK: AMP-activated protein kinase
Ang-2/ANGPT2: Angiopoietin 2
ANXA1: Annexin A1
AP-1: Activator protein-1
APC: Antigen-presenting cell
ApoE: Apolipoprotein E
AQP4: Aquaporin 4
ARG1: Arginase 1
ATF: Activating transcription factor
ATP1A3: Sodium pump α3 subunit protein
ATRA: All-trans-retinoicacid
B1R: Bradykinin receptor 1
BBB: Blood–brain barrier
Bcl3: B cell lymphoma 3
BDNF: Brain-derived neurotrophic factor
bFGF: Basic fibroblast growth factor
BiTE: Bispecific T cell engagers
BMAL1: Brain and muscle ARNT-like 1
BRD4: Bromodomain-containing protein 4
Breg: Regulatory B cell
BTK: Bruton’s tyrosine kinase
c-Kit: Receptor tyrosine kinase
C/EBPβ: CCAAT/enhancer binding protein β
CAF: Cancer-associated fibroblast
CAR-MΦ: Chimeric antigen receptor macrophages
CAR-T: Chimeric antigen receptor T-cell immunotherapy
CCR2: C–C motif chemokine receptor 2
CCL2: C–C motif chemokine receptor 2
CDK 4: Cycle-dependent kinase 4
CDKN: Cyclin-dependent kinase inhibitor
CED: Convection-enhanced delivery
CFH: Complement factor H
CFRT: Conventionally fractionated radiotherapy
cGAS/STING: Cyclic GMP-AMP synthase/stimulator of interferon genes
CHK1: Checkpoint kinase 1
CHOP: C/EBP homologous protein
CIS: Cytokine-inducible SH2-containing protein
CK2: Casein kinase 2
CLOCK: Circadian locomotor output cycles kaput
CMV: Cytomegalovirus
CNS: Central nervous system
COL6A3: Collagen type VI alpha 3 chain
COX2/PTGS2: Cyclooxygenase 2
CpG NP: CpG oligonucleotide nanoparticle
CPT1: Carnitine palmitoyl transferase I
CREB: Cyclic-AMP response binding protein
CSC: Cancer stem cell
CSF: Cerebrospinal fluid
CSF-1/M-CSF: Macrophage colony-stimulating factor-1
CSF-1R: Colony-stimulating factor 1 receptor
CSPG4: Chondroitin sulfate proteoglycan 4
CTLA-4: Cytotoxic T-lymphocyte-associated protein 4
CTLs: Cytotoxic T cells
CXCL: C-X-C motif chemokine ligand
CXCR: C-X-C motif chemokine receptor
DCs: Dendritic cells
DDRi: DNA damage response inhibitors
DHHC9: Asp-His-His-Cys 9
DHX9: DExH-box helicase 9
DIPG: Diffuse intrinsic pontine glioma
DM1: Mertansine
DMG: Diffuse midline gliomas
DNAM-1: DNAX accessory molecule-1
DNMT: DNA methyltransferase
DPP-4: Dipeptidyl peptidase-4
DUSP3: Dual-specificity phosphatase 3
EAE: Experimental allergic encephalomyelitis
EAG2: Ether-a-go-go 2
EC: Endothelial cell
ECM: Extracellular matrix
e-MDSCs: Early-stage myeloid-derived suppressor cells
EF2: Elongation factor-2
EGFR: Epidermal growth factor receptor
EGFRvIII: Epidermal growth factor receptor variant III
EIF4A3: Eukaryotic translation initiation factor 4A3
EMT: Epithelial to mesenchymal transition
ENTPD2: Ectonucleoside triphosphate diphosphohydrolase 2
EPHA2: EPH receptor A2
ErbB2: Erb-B2 receptor tyrosine kinase 2
ERK: Extracellular regulated protein kinases
ERS: Endoplasmic reticulum stress
EVs: Extracellular vehicles
EZH2: Enhancer of zeste 2
FA: Fatty acid
FAO: Fatty acid oxidation
FAT1: FAT atypical cadherin 1
FATP2: Fatty acid transport protein 2
Fbxw7: F-box and WD-40 domain protein 7
FCN1: Ficolin 1
FDA: Food and Drug Administration
FGL2: Fibroleukin 2
FHL-1: FH-like protein 1
FLNA: Filamin A
FLT3L: Fms-related tyrosine kinase 3 ligand
FN1: Fibronectin 1
FOXP3: Forkhead box protein P3
Fsp1: Ferroptosis suppressor protein 1
G-CIMP: Glioma CpG island methylator phenotype
G-CSF: Granulocyte colony-stimulating factor
GABA: γ-Aminobutyric acid
GAM: Glioma-associated macrophages/microglia
GASC: GBM-associated stromal cell
GBM: Glioblastoma
GBP5: Guanylate binding protein 5
GCN2: General control nonderepressible 2 kinase
GLUT1: Glucose transporters 1
GM-CSF: Granulocyte–macrophage colony-stimulating factor
GO: Gene ontology
GPNMB: Glycoprotein nonmetastatic melanoma protein B
GSCs: Glioblastoma stem cells
GTR: Gross total resection
HA: Hyaluronan
HDAC: Histone deacetylase
HDACI: Histone deacetylase inhibitors
HER2: Human epidermal growth factor receptor 2
HGG: High-grade gliomas
HIF: Hypoxia-inducible factor
HLA: Human leukocyte antigen
HMGB1: High mobility group protein B1
hnRNPA1: Hypoxia-inducible heterogeneous nuclear ribonucleoprotein A1
HNSCC: Head and neck squamous cell carcinoma
HSC: Hematopoietic stem cell
HSP72: Heat shock protein 72
HSPGs: Heparan sulfate proteoglycans
HSV: Herpes simplex virus
HVEM: Herpes virus entry mediator
ICAM: Intercellular adhesion molecule
ICBs: Immune checkpoint blockade
ICD: Immunogenic cell death
ICIs: Immune checkpoint inhibitors
ICOS: Inducible T cell costimulator
IDH1: Isocitrate dehydrogenase 1
IDO: Indoleamine2,3-dioxygenase
IFN-γ: Interferon γ
IGFBP6: Insulin-like growth factor-binding protein 6
IL: Interleukin
IMCs: Immature myeloid cells
iNOS: Inducible nitric oxide synthase
iNPC: Injured neural progenitor cells
IPS: Immune phenotype score
IRE1α: Inositol-requiring enzyme 1α
irSEs: Immune-related side effects
IRF: Interferon regulatory factor
iRGD: Internalizing RGD
IT: Immunotoxins
ITGαvβ5: Integrin αvβ5
ITGAM: Integrin subunit alpha M
ITGB2: Integrin subunit beta 2
JAK: Janus Kinase
K–M: Kaplan–Meier
KCNAB2/Kvβ2: Potassium voltage-gated channel subfamily a regulatory beta subunit 2
KDM6B: Lysine demethylase 6B
KLF4: Kruppel-like factor 4
KRAS: Kirsten rat sarcoma viral oncogene
LAG3: Lymphocyte activating 3
LAMP2A: Lysosomal-associated membrane protein 2A
LCK: Lymphocyte cell-specific protein-tyrosine kinase
LDH: Lactate dehydrogenase
LGALS1: Galectin-1
LGALS3BP: Galectin 3 binding protein
LGMN: Legumain
LIF: Leukemia inhibitory factor
LIFR: LIF receptor subunit alpha
LILRB2: Leukocyte immunoglobulin-like receptor subfamily B member 2
LOX1: Lectin-like oxidized low-density lipoprotein receptor 1
LPS: Lipopolysaccharide
LRP8: Low-density lipoprotein receptor-related protein 8
LXR: Liver X receptor
LXRβ: Liver X receptor β
M-MDSCs: Monocytic myeloid-derived suppressor cells
MAFB: MAF BZIP transcription factor B
MAPK: Mitogen-activated protein kinase
MCT: Monocarboxylate transporter
MEK: Mitogen-activated protein kinase
MET: Mesenchymal transformation
MDSCs: Myeloid-derived suppressor cells
MES-like: Mesenchymal-like
MGMT: Major histocompatibility complexes I
MHC I: Major histocompatibility complexes I
MIF: Macrophage migration inhibitory factor
miRNA: Micro RNA
MLPGs: Granulocyte-monocyte progenitors
MON: Monocytes
MPO: Myeloperoxidase
mRNAsi: MRNA stemness index
MSC: Mesenchymal stem cell
mTOR: Mammalian target of rapamycin
mTORC2: Mammalian target of rapamycin complex 2
MV: Measles virus
nAChR: Nicotinic acetylcholine receptor
NADPH: Nicotinamide adenine dinucleotide phosphate
NDV: Newcastle disease virus
NEAT1: Nuclear enriched abundant transcript 1
NF1: Neurofibromin 1
NF-κB: Nuclear factor kappa-B
NHE1: Sodium/hydrogen exchanger 1
NK cells: Natural killer cells
NKG2D: Natural-killer group 2 member D
NLRP3: NOD-like receptor thermal protein domain associated protein 3
NOX2: NADPH oxidase 2
NPC-like: Neural progenitor-like
NSCL: Non-small-cell lung cancer
OLFML3: Olfactomedin-like 3
OPC-like: Oligodendrocyte progenitor-like
OPN: Osteopontin
OS: Overall survival
OSM: Oncostatin M
OSMR: Oncostatin M receptor
OV: Oncolytic virus
PARP: Poly ADP-ribose polymerase
PB: Peripheral blood
PBMCs: Peripheral blood mononuclear cells
PD-1: Programmed cell death protein 1
PD-L1: Programmed cell death 1 ligand 1
PDE5: Phosphodiesterase 5
PDGF: Platelet-derived growth factor
PDGFRA: Platelet-derived growth factor receptor alpha
PDH: Pyruvate dehydrogenase
PDT: Photodynamic therapy
PE38QQR: Pseudomonas aeruginosa exotoxin A
PET: Positron emission tomography
PFS: Progression-free survival
pGBM: Primary GBM
PGE2: Prostaglandin E2
pHGG: Pediatric high-grade glioma
PI3K: Phosphoinositide-3 kinase
PLIN2: Perilipin-2
PMN: Morphology of neutrophils
PMN-MDSCs: Polymorphonuclear myeloid-derived suppressor cells
PNT: Peroxynitrite
pp65: Phosphoprotein 65
PPARγ: Peroxisome proliferator-activated receptor γ
PRC2: Polycomb repressive complex 2
Prkar1a: CAMP-dependent protein kinase regulatory type I-α
PTEN: Protein tyrosine phosphatase
PTM: Post-translational modification
PTX3: Pentraxin 3
PUFAs: Polyunsaturated fatty acids
PV: Poliovirus
RAPA: Rapamycin
Rb: Retinoblastoma
RCC: Renal cell carcinoma
rGBM: Recurrent GBM
RHAMM: Receptor for hyaluronic acid-mediated motility
RNS: Reactive nitrogen species
RORC1: Receptor-related orphan receptor γ
RORα: Retinoic acid related-orphan receptor α
ROS: Reactive oxygen species
RT: Radiotherapy
RTK: Receptor tyrosine kinase
SCLC: Small cell Lung cancer
scRNA-seq: Single-cell RNA sequencing
SDF1α: Stromal cell-derived factor 1 alpha
SDT: Sonodynamic therapy
SEMA4F: Ssemaphorin 4F
SERPINE1: Serpin family E member 1
SFPQ: Splicing factor proline and glutamine-rich
SHH: Sonic hedgehog
SHIP-1: Phosphatidylinositol-3,4,5-trisphosphate 5-phosphatase 1
SIRPA: Signal regulatory protein alpha
SLC7A11: Solute carrier family 7 members 11
SLE: Systemic lupus erythematosus
SLIT2: Slit guidance ligand 2
SNHG: Small nucleolar RNA host genes
SOCS3: Suppressor of cytokine signaling 3
SSAO: Semi carbazide-sensitive amine oxidase
ST: Spatial transcriptomics
STAT: Signal transduction and transcription factor
STR: Subtotal resection
TAMs: Tumor-associated macrophages
TAMCs: Tumor-associated myeloid cells
TBK1: TANK binding kinase 1
TCA: Tricarboxylic acid
TCF: Transcription factor
TCGA: The Cancer Genome Atlas
Td: Tetanus/diphtheria
TF: Transcription factor
TGF: Transforming growth factor
THBS1: Thrombospondin 1
TIGIT: T cell immune receptor with Ig and ITIM domains
TIIClnc: Tumor-Infiltrating Immune Cells-related lncRNA screening framework
TIL: Tumor-infiltrating lymphocyte
TIM-3: T-cell immunoglobulin and mucin-domain containing 3
TIME: Tumor immune microenvironment
TLR: Toll-like receptor
TME: Tumor microenvironment
TMZ: Temozolomide
TNF: Tumor necrosis factor
TNFAIP8L2: TNF alpha-induced protein 8 like 2
TNFSF9: TNF superfamily member 9
TP53: Tumor protein P53
TPO: Thrombopoietin
TRAIL-R: Tumor necrosis factor-related apoptosis-inducing ligand receptor.
TRET: Telomerase reverse transcriptase
Tregs: Regulatory T cells
TTF: Tumor treating field
US: Ultrasound
VCAM: Vascular cell adhesion molecule
VCAN: Versican core protein
VEGF: Vascular endothelial growth factor
VISTA: V-domain Ig suppressor of T cell activation
VNN2: Vascular non-inflammatory molecule 2
WT: Wild-type
WT1: Wilms tumor protein
ZNF148: Zinc finger protein 148
